# Integrative analysis of human protein, function and disease networks

**DOI:** 10.1038/srep14344

**Published:** 2015-09-24

**Authors:** Wei Liu, Aiping Wu, Matteo Pellegrini, Xiaofan Wang

**Affiliations:** 1 Department of Automation, Shanghai Jiao Tong University, Shanghai, 200240, China; 2 Institute of Biophysics, Chinese Academy of Sciences, Beijing, 100080; 3 Center for Systems Medicine, Institute of Basic Medical Sciences, Chinese Academy of Medical Sciences & Peking Union Medical College, Beijing 100005; 4 Suzhou Institute of Systems Medicine, Suzhou, Jiangsu 215123, China; 5 Department of Molecular, Cell and Developmental Biology, University of California, Los Angeles, CA, 90055.

## Abstract

Protein-protein interaction (PPI) networks serve as a powerful tool for unraveling protein functions, disease-gene and disease-disease associations. However, a direct strategy for integrating protein interaction, protein function and diseases is still absent. Moreover, the interrelated relationships among these three levels are poorly understood. Here we present a novel systematic method to integrate protein interaction, function, and disease networks. We first identified topological modules in human protein interaction data using the network topological algorithm (NeTA) we previously developed. The resulting modules were then associated with functional terms using Gene Ontology to obtain functional modules. Finally, disease modules were constructed by associating the modules with OMIM and GWAS. We found that most topological modules have cohesive structure, significant pathway annotations and good modularity. Most functional modules (70.6%) fully cover corresponding topological modules, and most disease modules (88.5%) are fully covered by the corresponding functional modules. Furthermore, we identified several protein modules of interest that we describe in detail, which demonstrate the power of our integrative approach. This approach allows us to link genes, and pathways with their corresponding disorders, which may ultimately help us to improve the prevention, diagnosis and treatment of disease.

Network methods are powerful tools for unraveling protein functions, protein-pathway associations, disease-gene and disease-disease associations. However, these disparate types of networks are more often studied independently of each other. To date, there has been great progress in the study of protein interaction networks. Previous research on protein networks^1^,^2^,^3^,^4^,^5^,^6^,^7^,^8^,^9^ mainly focused on analyzing the associations between genes, functional modules, and pathways. Using these approaches, usually only a fraction of detected protein modules have good mapping to biological functions or pathway annotations. Similarly, previous studies of disease networks^10^,^11^,^12^,^13^,^14^,^15^,^16^,^17^,^18^,^19^,^20^,^21^,^22^,^23^,^24^ mainly focused on disease classification and the prediction of disease genes. Recently, several groups have studied human disease networks^25^,^26^, to shed light on the relationship between disease genes and disease networks, as well as disease gene modules and their functional analysis. These methods start from diseasome^27^, which is a bipartite gene-disease network, from which we can derive two different disease networks: disease-disease networks and disease gene networks. Disease networks may help us to understand phenotype associations between proteins and diseases. Thus, a direct strategy for integrating protein interactions, protein function and disease patterns is still absent, and the interrelated relationships among these three levels have been poorly investigated.

To better understand the relationships between these three network types, we present a multi-network systematic analysis method. Using our approach, protein modules are determined directly from topological modules using the network topological algorithm we previously developed (NeTA^28^). Traditionally, a protein module is defined as a group of proteins that carry out similar functions. These functions are associated with the same pathway, and could be associated with a particular disease. Here we focus on three distinct protein modules: topological, functional and disease modules^25^,^26^. Topological modules represent a locally dense structure in protein-protein interaction (PPI) networks; function modules represent the aggregation of proteins of related function in a function network; disease modules represents a group of proteins that share a common disease phenotype within a disease network. Though the three types of modules are derived from three different types of networks, they can be closely interrelated and highly overlapping^25^.

Starting with the protein interaction dataset from Hippie^29^, we identified 136 topological modules with NeTA, 136 corresponding functional modules (annotated using Gene Ontology^30^), and 139 disease modules annotated using OMIM^31^ and GWAS^32^. To our surprise, most functional modules (70.6%) are highly consistent with the corresponding topological modules, and most disease modules (88.5%) are fully covered by the corresponding functional modules, and have significant pathway annotations. By systematically integrating the three levels of networks and protein modules, we found that our multi-level method for biological interpretation has distinct advantages over approaches that only consider subsets of data and annotations. Many interesting modules are found that could not be easily discovered by only one data type. For example, we identified several protein interaction modules that allowed us to connect inflammatory responses to Alzheimer’s disease, suggesting that this pathology may have a strong inflammatory component. Moreover, in many modules, we found that a subset of genes is associated with specific functions or diseases, allowing us to identify genes and pathways with their corresponding disorders. The approach we present here not only provides an avenue for network integration, but also promises to shed light on the prevention, diagnosis and treatment of complex diseases.

## Results

### The integrated multi-networks mapping method

**Figure 1:**
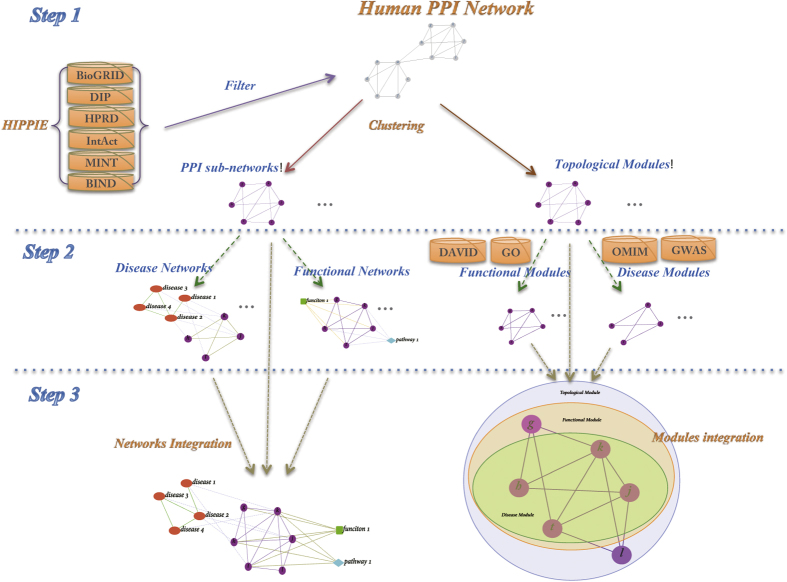
The schematic of multi-networks mapping method. There are three steps in multi-networks mapping method, including clustering, mapping and integrative analysis.

Figure 1 shows a schematic of our overall approach, the framework of the integrated multi-networks mapping method, which consisted of three steps. First we determined the topological modules from a human PPI network. Next, we annotated all topological modules using Gene Ontology (GO), to obtain functional modules. Finally, we included OMIM and GWAS data to obtain disease modules. Thus, three levels of networks were constructed and modules were identified at each level, including a protein network and its topological modules, a function network and its functional modules, and a disease network and its disease modules. Finally, we integrate the three types of networks and modules, to discover modules that have coherent function and disease interpretation, leading to new associations that are not evident when analyzing only a single type of network.

### Protein Networks and Topological Modules

**Figure 2:**
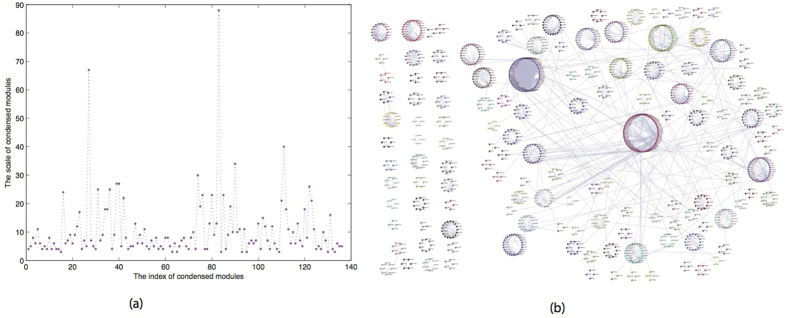
The constructed human PPI network. (**a**) The size of condensed modules of PPI run from 3 to 88. (**b**) The 136 detected topological modules and corresponding PPI network. Different color denotes different topological modules.

The human PPI network was constructed based on the HIPPIE^29^ and IRefWeb^33^ databases, which results in a network of 2484 direct physical interactions among 1830 proteins. We detected 136 large modules and 185 small modules (most of which only contain two proteins) by applying the network topology algorithm NeTA^28^. Here we analyze the 136 larger topological modules (as shown in Supplementary Table 1). This PPI network contains 1390 proteins, and 2228 interactions (Fig. 2b), which results in 76% of the proteins being associated with 89.7% of the interactions of the PPI network. As Fig. 2a shows, the size of larger modules runs from 3 to 88. In Fig. 2b, different colors represent different modules, and we can clearly see that this network has a modular structure. The modularity Q^34^ is 0.91385, which means these modules have significantly more community structure than a random zero-model.

### Function Networks and Functional Modules

**Figure 3:**
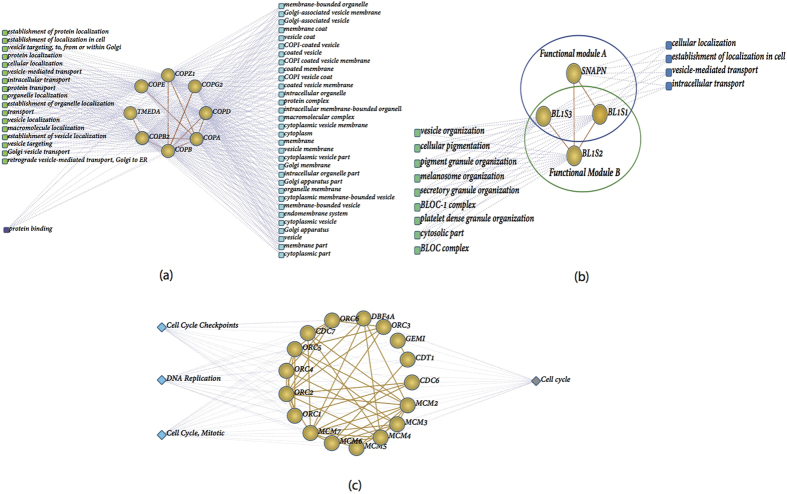
Function network. (**a**) The protein-function network of topological module 3. (**b**) The protein-function network of topological module 9. Round denotes proteins, and rectangles denote functions. Green denotes BP, cyan denotes CC, and deep purple denotes MF. (**c**) The protein-Pathway network of topological module 23. Round denotes proteins, and diamond denotes pathway. Orange denotes KEGG pathway, and light green denotes Reactome pathway.

To build a function network, we mapped each topological module into Bingo^35^, and analyzed the GO enrichments of each module at three levels of Gene Ontology (GO) slim, using annotation from Biological Process, Cellular Component, and Molecular Function ontologies^30^. A functional module was defined as a group of genes in a topological module that is associated with a specific GO term. In total, we found 136 functional modules (as shown in Supplementary Table 2) with at least three proteins. If we don’t consider the unannotated proteins (there is only one protein in our network that has no annotations in Bingo), we found that 96 (70.6%) of our topological modules are fully covered by functional modules (i.e. all the proteins map to the same function term). For example, topological module 3 consists of 8 proteins, COPA, COPB, COPD, COPE, COPB2, COPZ1, COPG2, and TMEDA (Fig. 3a). All eight proteins share the same BP function “Golgi vesicle transport” (p-value is 2.4E-16), as well as the same CC function “cytoplasmic vesicle membrane” (p-value is 9.71E-15). In general, all other modules are covered by at most two function modules. An example of this is topological module 9, which has four genes: BL1S1, BL1S2, BL1S3 and SNAPN (Fig. 3b), that are associated with two functional modules: BL1S1, BL1S2, BL1S3 (“cellular pigmentation”, p-value is 4.01E-7) and BL1S1, BL1S3 and SNAPN (“vesicle-mediated transport”, p-value is 3.64E-3).

Furthermore, each topological module was annotated using the DAVID^36^,^37^ online analysis tool to identify pathway enrichment (see Methods), and construct protein-pathway networks. We found 88 topological modules (as shown in Supplementary Table 4) significantly associated with a pathway, and pathway genes are closely related with corresponding functional module genes. For example, as Fig. 3c shows, topological module 23 has 17 proteins, all of which are annotated as “DNA Replication pathway”(p-value is 1.09E-25), as well as “nucleoplasm” (p-value is 3.06E-20).

### Disease Networks and Disease Modules

**Figure 4:**
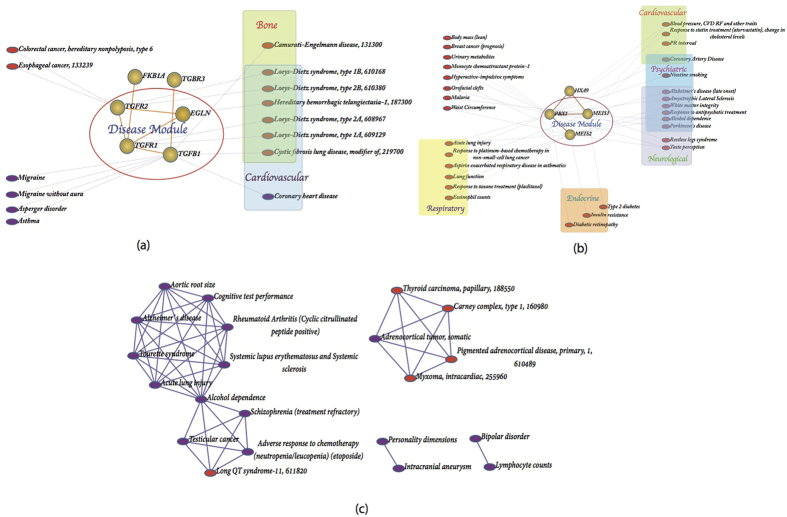
Disease network. (**a**) The disease-gene network of topological module 6. (**b**) The disease-gene network of topological module 11. The Round denotes proteins, and ellipse denotes diseases. Red denotes OMIM diseases, and purple denotes GWAS diseases. (**c**) The Disease-disease network of topological module 33. Nodes denote diseases and link between two diseases denote they share at least one disease gene.

To build the relationship between proteins and diseases, we mapped each topological module to the OMIM^31^ and GWAS^32^ databases. In total, 109 topological modules have disease genes, and 139 significant disease modules (as shown in Supplementary Table 3) were identified. One topological module may corresponds to one or more than one disease module. For example, topological module 6 has six genes, of which EGLN, TGFB1, TGFR1, and TGFR2 are disease genes associated with Bone and Cardiovascular diseases, which we therefore label as a disease module (Fig. 4a). Another example, topological module 11, with 4 genes, contains MEIS1, MEIS2 and PBX1, which are disease genes associated with Cardiovascular, Neurological, Psychiatric, Endocrine and Respiratory diseases, and these were also defined as a disease module (Fig. 4b).

The study of associations between diseases is also potentially interesting, as it could help understand relationships between complex syndromes. We constructed a disease-disease network for each topological module. Nodes are diseases that are associated with one gene or multiple genes in the topological module, and edges between two diseases denote that they share at least one disease gene. Closely related diseases may be associated with complex syndromes. For example, Fig. 4c shows the disease-disease network of topological module 33: Thyroid carcinoma (papillary), carney complex (type 1), Adrenocortical tumor (somatic), Pigmented adrenocortical disease (primary) and Myxoma (intracardiac), which are all cancers, and besides Myxoma, are also endocrine pathologies.

### Integrative Analysis

Considering protein interaction, function, and disease networks independently significantly limits our ability to carry out a systematic study of the data. As a result, we integrated protein, function and disease networks, in order to annotate protein modules according to their function and disease associations, to gain a systematic view of these relationships. In addition, to view the relationship between different types of modules, we also integrated topological modules with functional and disease modules. If a disease module is highly overlapping (over half of proteins) with a functional module, then we defined its corresponding topological module as a non-trivial protein module; if a disease module is highly overlapping (over half of proteins) with a pathway module, then we defined its corresponding topological module as a significant protein module. Using this integrative analysis, we identified 69 non-trivial protein modules, and 47 significant protein modules in our PPI network. We discuss a few examples below.

**Figure 5:**
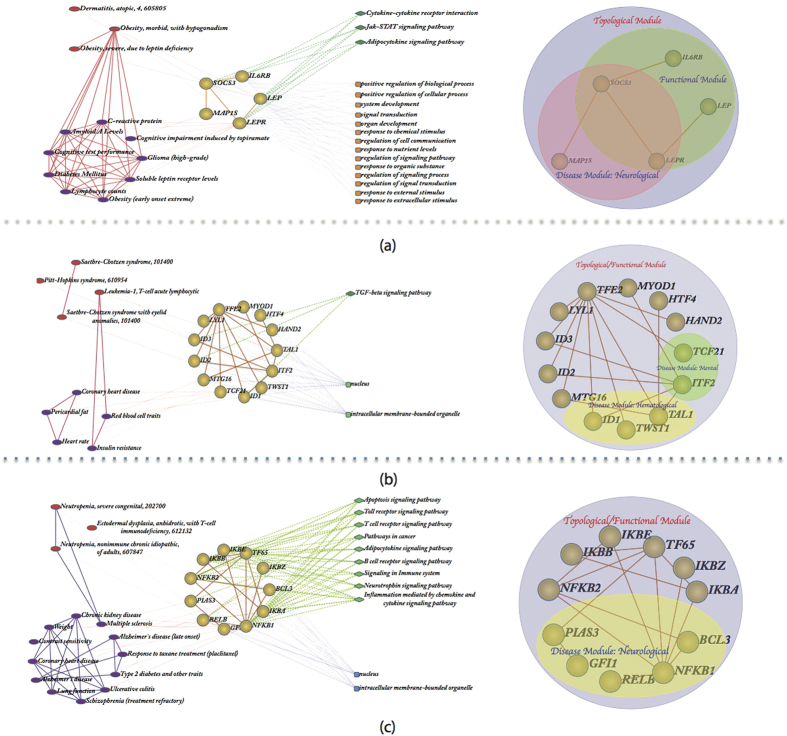
Integrative analysis of networks and non-trivial protein modules. (**a**) The integrative analysis of networks and modules of topological module 55. (**b**) The integrative analysis of networks and modules of topological module 82. (**c**) The integrative analysis of networks and modules of topological module 113. On the left is the integration of protein networks, function network and disease network, and on the right is the integration of corresponding topological module, functional module and disease module.

Figure 5a shows an intriguing non-trivial protein module (Topological module 55) that connects leptin and the leptin receptor to the inflammatory cytokine receptor IL6RB. There is extensive literature implicating leptin to obesity and diabetes^38^. However, this module shows us that these disorders are also associated with inflammation (through IL6). There is increasing recognition that many metabolic disorders, such as diabetes, are also associated with higher levels of inflammation^39^. Thus this module suggests that anti-inflammatory treatments could be coupled with weight loss regimes to address metabolic disorders.

Figure 5b shows another non-trivial protein module (Topological module 82) with a number of factors that likely play a significant role in hematopoietic development. Specifically, Tal1 is a master regulator of T cell development, and inhibits the production of cardiac cells^40^. It is therefore interesting to see that several of the genes in this module are associated not only with T cell development, but also with heart disease and heart rate.

Figure 5c describes a complex of proteins associated with NfkB (Topological module 113), a master regulator of inflammatory responses. One interesting observation is that several genes in this module are associated with Alzheimer’s disease. This is of interest, as there is growing recognition that Alzheimer’s disease may be associated with inflammation, and its risk is elevated by metabolic disorders, such as diabetes^41^. Thus this non-trivial protein module allows us to make the critical connection between these two important disorders, and the basal inflammatory responses of cells.

**Figure 6:**
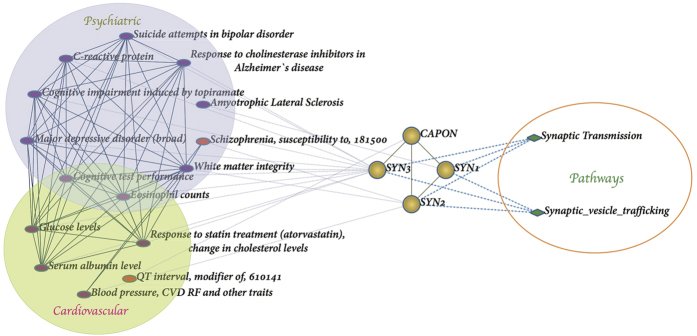
Significant protein modules. The Disease network and protein-pathway network of topological module 24. Round denotes proteins, diamond denote pathway and ellipse denotes diseases.

Figure 6 shows a significant protein module (Topological module 24) with four genes, SYN1, SYN2, SYN3 and CAPON. Of these SYN1, SYN2, and SYN3 are all associated with psychiatric disease, and the synaptic transmission and synaptic vesicle trafficking pathway.

In addition, topological module 3 and 51 are also interesting non-trivial protein modules. All the proteins of module 3 are involved in Golgi vesicle transport, and most are also involved in the membrane trafficking pathway, and associated with Alzheimer’s disease. All the proteins of module 51 are associated with translation initiation factor activity, and in the Metabolism of proteins pathway, and most are also associated with liver disease.

### Comparison with existing methods

In recent years, a number of methods have been developed to identify functional modules^1^,^2^,^3^,^4^,^5^,^6^,^7^,^8^,^9^ and disease modules^10^,^11^,^12^,^13^,^14^,^15^,^16^,^17^,^18^,^19^,^20^,^21^,^22^,^23^,^24^ in PPI networks. Most methods to identify disease modules are disease protein prioritization methods. To evaluate the relative performance of our method, we compare our results with two representative methods that can identify functional and disease modules. One is the Markov Cluster Algorithm (MCL)^42^, which is based on random flow (We use the default settings, inflation parameter r = 2) and the other is random walker (RW)^43^ that wanders from node to node along the links of the network. After every move the walker is reset to a randomly chosen seed gene with a given probability r (we use r = 0.4).

**Figure 7:**
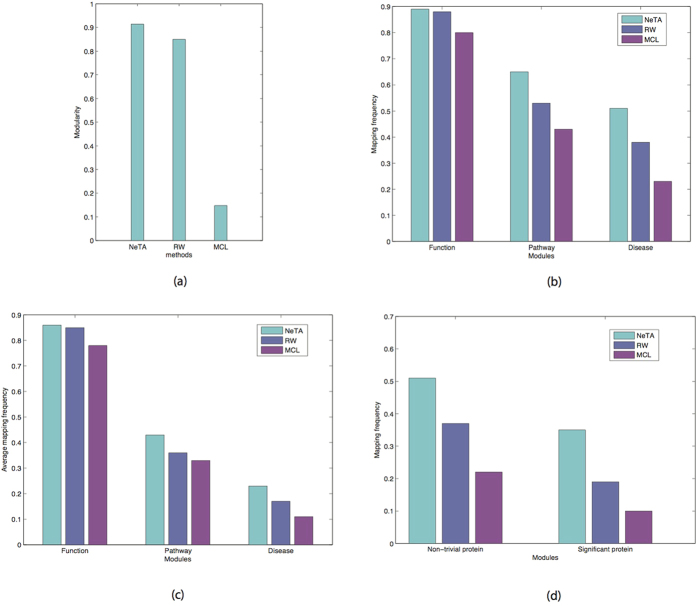
Performance evaluation of NeTA. (**a**) The modularity of three representative clustering algorithms. (**b**) We mapped the detected topological modules to function, pathway and disease database respectively, and computed the mapping frequency for each algorithm. (**c**) The average mapping frequency of all detected modules are counted for the three algorithms. (**d**) The mapped frequency of detected non-trivial protein modules and significant protein modules.

Figure 7(a) shows the modularity results from NeTA, MCL and RW, which can qualify the clustering quality of the topological modules. NeTA performs better than the other two methods. Figure 7(b) shows the mapping frequency of the three methods based on the topological modules that were identified. As expected, among the three kinds of modules, no matter what method was used, we identify more functional than disease modules. NeTA identified more functional modules than MCL and RW, and also identified more pathway and disease modules. Figure 7(c) shows the average mapping frequency of the three methods based on topological modules that were identified. Fore each method, we count each functional/disease module mapping frequency, and take the mean value, which measures the mapping accuracy of each method. We can see that the mapping accuracy of NeTA is higher than other two methods. Figure 7(d) describes the mapping frequency of non-trivial protein modules and significant protein modules. Again, NeTA finds more non-trivial and significant protein modules. Overall, we find that our method performs better than the other algorithms for all kinds of protein modules.

### Systematic evaluation analysis

To systematically evaluate the power of our method to infer function-disease associations, we constructed a benchmark network based on the OMIM^31^ and MIPS human complex database^44^. We filtered human complex PPI with disease genes from OMIM, and constructed a network with 1460 proteins and 4107 protein–protein interactions. In this setting, there is at least one disease gene in each interaction. We use this as a benchmark network, to identify disease, protein complex and disease-complex modules, and compare our results with the MCL and RW algorithms.

**Figure 8:**
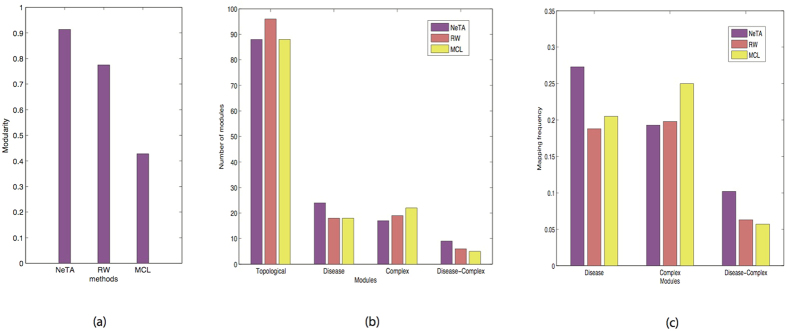
Systematic evaluation of NeTA based on a benchmark network. (**a**) The modularity of three clustering algorithms. (**b**) We mapped the detected topological modules to OMIM disease database and MIPS human complex database respectively, and counted the mapped number of disease modules, complex modules and disease-complex modules respectively, and compared them with the other two algorithms. (**c**) The mapped frequency of disease modules, complex modules and disease-complex modules respectively, in comparison with the other two algorithms.

Figure 8(a) shows the resulting modularity of NeTA, MCL and RW against this network. Among them, NeTA has the highest modularity, which shows it can obtain better module structure than the other two methods. Figure 8(b) shows the number of different modules identified by the three methods. RW identified the most topological modules, and MCL identified the most complex modules, and NeTA identified the most disease modules and disease-complex modules. Figure 8(c) shows the mapping frequency of different modules identified by the three methods. Disease and protein complex modules can only map to approximately 20% of topological modules, and even fewer disease-complex modules. Overall, we find that our method performs competitively with the other algorithms.

## Discussion

Protein interaction, function and disease networks can be clustered into cohesive groups. Accordingly, these cohesive groups can be defined as topological, functional and disease modules. Most previously published approaches that analyze these datasets only focus on a subset of the three levels. For example, most work on PPI networks only focus on topological modules and their corresponding functional modules^1^,^2^,^3^,^4^,^5^,^6^,^7^,^8^,^9^. Other approaches analyze pathway enrichment of modules. Similarly, most of the work on disease networks focuses on disease genes and their classification^10^,^11^,^12^,^13^,^14^,^15^,^16^,^17^,^18^,^19^,^20^,^21^,^22^,^23^,^24^. As a result, an integrative analysis of all three levels of modules and networks has yet to be performed.

Here we present a systematic method for combining protein interactions, functions and disease networks, resulting in an integrative analysis that yields topological, functional and disease modules. Other integrative approaches start from Diseasome^27^ to detect disease modules, and then identify functional and topological modules based on these. In contrast, we start from a human PPI network and detect 136 topological modules (as shown in Supplementary Table 1) using NeTA. We then annotate these topological modules using GO, OMIM and GWAS, and find corresponding functional and disease modules, leading to the construction of networks for each of the three levels. To visualize the associations among the three levels of modules and networks, we integrated the three levels together, and found that they generate new insights into protein network analysis. This approach allowed us to identify many interesting modules, which can’t be fully annotated only using a single type of data. For example, we identified several protein interaction modules that allowed us to connect inflammatory responses to Alzheimer’s disease, suggesting that this pathology may have a strong inflammatory component.

In topological module 3, which includes eight proteins, we found that all the proteins are involved in Golgi vesicle transport, and that COPA, COPB1, COPB2, COPD, COPE, COPG2 and COPZ1 belong to an octamer protein complex^45^,^46^,^47^. In addition, COPA, COPB1, COPB2, COPD, COPE and COPZ1 are involved in the membrane trafficking pathway, and COPA, COPB1, COPB2, TMED10 and COPG2 are associated with Alzheimer’s disease^45^,^46^,^47^. Moreover, COPD is associated with increased risk for Mild Cognitive impairment, the earliest phase of Alzheimer’s disease, and COPZ1 is involved in intracellular trafficking^45^,^46^,^47^. Impairment of intracellular trafficking has been implicated in the pathogenesis of Alzheimer’s disease, so COPZ1 may be associated with Alzheimer’s disease. COPE is associated with depressive disorder, which is similar to the later phase of Alzheimer’s disease, suggesting that COPE may also be associated with Alzheimer’s disease.

Topological module 5 includes 11 genes, which are all located in the membrane. DMD, DTNA, NOS1, SNTA1, MAST2 and VAC14 are Type 2 diabetes disease genes^45^,^46^,^47^, SCN5A is a diabetes mellitus disease gene, SNTB2 and UTRN are type 1 diabetes disease genes^46^,^47^; SNTB1 controls glucose levels, and could be a potential diabetes disease gene. MAST1 is an important paralog of MAST2^45^, and phosphorylation of DMD or UTRN may modulate their affinities for associated proteins, and thus may also be associated with diabetes mellitus.

Topological Module 51 includes 11 genes, which are components of the eukaryotic translation initiation factor 3 (eIF-3) complex, which is required for several steps in the initiation of protein synthesis^45^. All these genes are related with translation initiation factor activity, and in the Metabolism of proteins pathway. In fact, the eIF-3 complex is composed of 13 subunits, and EIF3J and EIF3M are not included in this module^45^. The most interesting observation is that all these genes are associated with liver diseases: EIF3A, EIF3B, EIF3C, EIF3D and EIF3G are all associated with Liver Failure^47^, Acute Hepatitis; EIF3E, EIF3F and EIF3K are associated with Liver Neoplasms^47^; EIF3H is associated with Carcinoma Hepatocellular^47^; EIF3I is associated with clonorchiasis^47^. Furthermore, EIF3L has a lower level of expression in liver cancer^45^,^46^,^47^. Therefore, the eIF-3 complex may be associated with liver disease as well.

As these examples illustrate, our work has the potential to inform the prevention, diagnosis and treatment of disease. It is often difficult to accurately identify potential gene targets based on GWAS, even though many GWAS variants are strongly associated with diseases. Although GWAS to protein associations affect the number of disease modules we can identify, we do not expect that these uncertainties significantly change the analysis results we obtained. In conclusion, our integrative analyses are still far from providing important therapeutic breakthroughs, which require substantial follow-up investigation. Nonetheless, they provide a wealth of hypothesis that could lead to clinical improvements in the future. To make these hypotheses more robust, in subsequent work we intend to improve the method of noise reduction and data integration, split bigger modules into smaller ones, and integrate more levels of data together to improve our system level understanding of these complex diseases.

## Methods

### Data source

HIPPIE^29^ is a human PPI database, and currently contains more than 156,000 interactions of ~14,500 human proteins. It integrates multiple major expert-curated experimental PPI databases, and all interactions have an associated normalized confidence score. Here we selected six public human PPI databases: BioGrid^48^, DIP^49^, HPRD^50^, IntAct^51^, MINT^52^ and BIND^53^ as our data sources based on the HIPPIE database, and identified high-confidence interactions based on the HIPPIE scoring system. To obtain more reliable interactions, we only keep those that are found in at least two pubic databases and are classified as high-confidence interactions.

### Network Construction

#### Protein Network

We extracted high-confidence interactions from the HIPPIE database, and took direct physical interactions that cross multiple species based on the IRefWeb^33^ database to construct the final human PPI network. IRefWeb is a web interface to protein interaction data consolidated from 10 public databases. It can automatically crop the PPI dataset to produce a subset of higher-quality interactions, which aids the generation of more meaningful organism-specific interaction networks. In this network a node denotes a protein, and a link represents a protein-protein interaction.

#### Function Network

There are two kinds of function networks: one is a Protein-function network, and the other is a protein-pathway network. Protein-function networks are obtained by connecting proteins of each topological module (defined below) with corresponding GO biological processes, cellular localizations and molecular functions. In what follows we only used the third level under of GO slim terms^30^. In these networks nodes are proteins or GO terms. Edges are drawn between a protein and a term when a significant association between them exists (based on a hypergeometroc test P value between the functional modules and protein module). Protein-pathway networks are constructed by connecting proteins of each topological module with corresponding pathway annotations (pathway sources see below).

#### Disease Network

By mapping each topological module into the OMIM and GWAS database, we constructed two types of disease networks: Disease-gene networks and disease-disease networks. Disease-gene networks connect the genes in each topological module with their associated diseases. Disease-disease networks connect pairs of diseases if they share at least one disease gene.

### Protein Module Detection

#### Topological Modules

High aggregation is an essential characteristic of biological networks, and it reflects high modularization of gene networks. The network we use was first clustered into different sizes of topological modules before further analysis. Accurately identifying topological modules of a biological network is still challenging. Here we detected topological modules based on a network topological algorithm NeTA^28^ (NeTA can detect sparse and small modules, and is competitive with other methods^28^), and we only consider those topological modules that contain at least three proteins.

#### Functional Modules

To evaluate the biological significance of these topological modules, we analyzed Gene Ontology)^30^ enrichment of each topological module with the Bingo^35^ plugin in Cytoscape^54^ with a threshold P-value < 0.05 based on the Hypergeometric test, and corrected by the Benjamini & Hochberg False Discovery Rate (FDR). Bingo generates hierarchical functional annotations based on GO slim. To obtain coherent functional modules, we only chose functions in the third level of GO slim^30^. We consider a group of proteins in a single topological module as a functional module if and only if at least one function can cover all these proteins.

#### Disease Modules

Online Mendelian Inheritance in Man (OMIM)^31^ is a comprehensive, authoritative compendium of human genes and genetic phenotypes. Genome-wide association studies (GWAS^32^) examine common genetic variants in populations to see if they are associated with a trait. GWASdb^55^ is a database that combines collections of GVs from GWAS together with their functional annotations and disease classifications. MalaCards^56^ is an integrated searchable database of human maladies and their annotations, modeled on the architecture and richness of the popular GeneCards database of human genes. We detected disease modules based on known disease-gene associations extracted from OMIM and GWASdb and the disease classification of MalaCards online annotations. If more than two proteins have associations with the same disease type within certain topological module, then we take these proteins as a disease module. Here we classify diseases into 15 kind of phenotypes: Neurological, Ophthamological, Cardiovascular, Bone, Dermatological, Endocrine, Metabolic, Cancer, Immunological, Psychiatric, Hematological, Renal, Respiratory, Ear, Nose, Throat and Gastrointestinal by integrate MalaCards database and Barabási *et al*. method^27^.

### Pathway enrichment analysis

Information on the biological pathways that the module-related genes are involved in for each topological module was retrieved from DAVID^36^,^37^ online analytical tools. We set a corrected P-value <0.05 as the threshold used for enrichment analysis of pathways. The pathway databases we used are KEGG^57^ and REACTOME^58^, PANTHER^59^ and BIOCARTA^60^.

### Systematic Analysis Method

Here we use a systematic analysis method to discover significant modules. The specific steps are shown in Fig. 1. First we integrate 6 different human PPI databases; second, we integrate the HIPPIE database with IRefWeb database to obtain human protein “interactome” network; third, we divided the network into PPI sub-networks (topological modules) based on NeTA algorithm; fourth, construct corresponding function networks (based on detected functional modules) and disease networks (based on detected disease modules); lastly, to view the relationship of different types of modules more clearly, we integrate topological modules, functional modules and disease modules together, to generate an integrative analysis of different network levels, including protein, function and disease networks. We annotated the proteins within each module with the third level of GO slim.

## Additional Information

**How to cite this article**: Liu, W. *et al.* Integrative analysis of human protein, function and disease networks. *Sci. Rep.* **5**, 14344; doi: 10.1038/srep14344 (2015).

## Supplementary Material

Supplementary Information
