# Supplementary material for: Integrative analysis of human protein, function and disease networks
Source: Sci Rep. 2015 Sep 24;5:14344. doi: 10.1038/srep14344 (PMC4585831; doi:10.1038/srep14344)
Supplement: Supplementary Information [file srep14344-s1.pdf]

Supplemental Information: Integrative analysis of human protein, function and disease networks

Wei Liu, Aiping Wu, Matteo Pellegrini & Xiaofan Wang

Supplementary Tables

Supplementary table 1: List of genes of topological modules

T_Modules	Gene_name	T_Modules	Gene_name	T_Modules	Gene_name
Module 1	ACTG	Module 47	CLD4	Module 90	ACTN2
	COF2		CLD3		ADRB1
	CAP1		CLD7		KCC2A
	DEST		CXA1		CDK5
Module 2	RHOA		ZO1		DLG1
	RND3		CLD5		DLG2
	GDIR1		CLD8		DLG3
	ROCK1		CLD6		DLG4
	RTKN		CLD2		ERBB4
Module 3	COPD		CLD1		GRIK2
	COPA		ZO2		NMDZ1
	COPB		ZO3		NMDE1
	COPB2		OCLN		NMDE2
	TMEDA	Module 48	HLTF		NMDE3
	COPE		UBE2N		NMDE4
	COPZ1		UB2V1		IL16
	COPG2		UB2V2		KCNA1
Module 4	CRYAA		ZNRF2		KCNA4
	CRYAB		TM189		IRK2
	HSPB1	Module 49	ATX3		IRK4
	HSPB2		UFD1		IRK10
	HSPB8		TERA		IRK15
	HSPB6		UBE4B		MAP1A
Module 5	DMD		UBXN1		FZD7
	DTNA		ANKZ1		SYGP1
	NOS1		NPL4		CD5R1
	SCN5A		NSF1C		DLGP2
	SNTA1		SYVN1		DLGP1
	SNTB1	Module 50	RAB3A		CRIPT
	SNTB2		RAB3B		LDB3
	UTRO		1433F		NLGN1
	MAST1		RIMS2		SI1L1
	MAST2		RP3A		SEM4C
	VAC14		RIMS1		LIN7B

Module 6	EGLN	Module 51	EIF3E	Module 94	CREB1
	FKB1A		EIF3A		CBP
	TGFB1		EIF3B		EP300
	TGFR1		EIF3C		GATA1
	TGFR2		EIF3D		HIF1A
	TGBR3		EIF3F		MED1
Module 7	ERCC2		EIF3G		RARG
	ERCC3		EIF3H		VHL
	TF2H2		EIF3I		NCOA3
	TF2H3		EIF3K		HDAC4
Module 8	IF4A1		EIF3L		CITE2
	IF4A2	Module 52	TNR6	Module 95	EFNB2
	IF4E		CASP8		EPHB2
	4EBP2		TRADD		YES
	IF4G1		FADD	Module 96	ANDR
Module 9	BL1S1		CFLAR		ESR1
	SNAPN	Module 53	5HT2C		PO2F1
	BL1S2		SSR3		PO4F1
	BL1S3		MPDZ		PO4F2
Module 10	CSN1		PKHA2		NCOA2
	COT1	Module 54	ADA2B	Module 97	CTBP2
	CSN3		ADA2C		EZH1
	CSN2		EI2BA		EZH2
	CSN8		EI2BD		RBBP4
	CSN6		EI2BG		EED
	CSN5		EI2BB		SUZ12
	CSN4		EI2BE		FOG1
Module 11	HXA9	Module 55	IL6RB	Module 98	FGF1
	MEIS1		LEP		FGF2
	MEIS2		LEPR		FGFR1
	PBX1		SOCS3		FGFR2
Module 12	GLI1		MAP1S		GRB14
	GLI3	Module 56	CFTR		FRS2
	SKI		MOES	Module 99	GDIB
	ZIC1		PGFRB		RAB8A
	ZIC2		NHRF3		MYO5B
	ZIC3		PLCB3		RAB5A
Module 13	GNAO		PTEN		RB11A
	RGS20		NHRF2		RABE1
	RGS19		NHRF1		OPTN
	RIC8A	Module 57	NRX3A	Module 100	GNAI2
Module 14	DPOE1		NLGN1		GNAI3
	DPOE2		NLGN3		GBB1
	DPOE3		NLGN2		GBB2

	CHRC1	Module 58	PLXB1		GBG3
Module 15	T2EA		PLXB3		GBG4
	T2EB		ARHGB		GBG5
	TF2H1		ARHGC		GBG7
Module 16	CCNC		PLXB2		GBG10
	CDK7	Module 59	NFE2		GBG11
	CDK8		ASPP2		GBG1
	CSTF2		YAP1		NUCB1
	CSTF3		WBP1		GBB4
	TF2B	Module 60	ATG12	Module 101	GRB10
	NEDD4		ATG7		IGF1R
	RPB1		GBRAP		ARHGC
	RPB2		GBRL2		PERQ1
	RPB3		ATG4B	Module 102	GRIA1
	RPB4		GBRL1		GRIA2
	RPAB1		ATG3		GRIA3
	RPAB2		MLP3B		GRIA4
	RPB7	Module 61	ACTN1		GRIK1
	RPAB3		DRD3		GRM3
	RPB11		PDIA3		GRM7
	RPAB4		GTR1		NSF
	RPAB5		RGS19		SDC2
	SPT6H		GIPC1		SDCB1
	TCEA1		KIF1B		SC6A3
	UBA1		CLIC6		ULK1
	WBP2	Module 62	U2AF1		PICK1
	RNF11		PRP4		GRIP1
	PMEPA		PRPF3		GRAP1
Module 17	AAPK1		U2AF2	Module 103	AHR
	AAPK2		PRP31		ARNT
	AAKB1	Module 63	PVRL1		FKBP4
	AAKB2		PVRL2		GNA12
	AAKG1		PVRL3		GCR
	AAKG2	Module 64	ERF1		HS90A
Module 18	RARA		ERF3A		PPP5
	RXRA		PCBP1		SIM1
	THA		ERF3B		SIM2
	THB		PABP1		AIP
	NRIP1		PAN3		CDC37
	TIF1A	Module 65	AP2A2		CHRD1
	MED25		EPS15	Module 104	H2AX
Module 19	FINC		EPN1		H33
	ITA6	Module 66	POLH		H33
	ITA2		MD2L2		SSRP1

	ITA3		POLI		KAT5
	ITA5		POLK		SP16H
	ITA7		REV1		ASF1B
	ITB1	Module 67	LSM6	Module 105	DCTN1
	NDKA		LSM5		HD
	NDK3		LSM4		SH3G3
Module 20	FBLN2		LSM1		HAP1
	PGBM		LSM3	Module 106	ANXA6
	LAMB1		LSM7		RASH
	LAMC1		LSM2		RASK
	NID1	Module 68	RAD17		PK3CD
	NID2		RFC1		PK3CG
Module 21	MAD1		RFC2		RGL2
	MAX		RFC3		GNDS
	MXI1		RFC4		RASA1
	MYC		RFC5		SHOC2
	MYCL1		CTF18		RGL1
	MYCN		DCC1		PI3R5
	SMC3	Module 69	UBF1		RASF5
	MAD4		RPAC1	Module 107	BTG1
	MAD3		RPA1		FBRL
Module 22	SMN		RPAC2		ANM1
	SMN		RPA49		ILF3
	RSMB	Module 70	CXAR		BTG2
	SMD1		NUMB		HNRPR
	SMD2		LNX1	Module 108	IGBP1
	SMD3		LNX2		PP4C
	RUXE	Module 71	ABL1		PPP6
	RUXF		EPS8		TIPRL
	RUXG		PTN12	Module 109	ILK
	GEMI2		PPIP1		LIMS1
	WBP4		ABI1		PARVA
	DDX20		VINEX	Module 110	CADH2
Module 23	CDC6		PTN18		EGF
	MCM2		ENAH		EGFR
	MCM3	Module 72	AP1B1		GHR
	MCM4		AP2B1		INGR2
	MCM5		AP1G1		INSR
	MCM6		ATM		IRS1
	MCM7		AP1S1		JAK2
	ORC1		AP1S2		LYN
	ORC2		AP1G2		PDE4A
	ORC4		AP1M1		PTN1
	ORC5		EPN4		PTN12

	CDC7		AP1M2		SHC1
	DBF4A	Module 73	AES		STA5A
	ORC6		HMGB1		STA5B
	ORC3		SIX6		SOCS1
	GEMI		SIX3		IRS2
	CDT1	Module 74	AKT1		DOK2
Module 24	SYN1		ARRB1		SH2B2
	SYN2		ARRB2		SH2B1
	SYN3		ATF4		SHCBP
	CAPON		CCNG1	Module 111	ACTN1
Module 25	DLX2		CHD3		ATF3
	MEOX1		KC1D		DACH1
	MEOX2		FKBP3		DAXX
	MSX1		FOXO1		DTX1
	PAX1		HMGB1		EF2
	PAX3		GRP75		FOS
	LHX2		MDM2		FOSL2
Module 26	IRF5		M3K5		JUN
	IRF7		MYB		JUNB
	MYD88		PEPL		JUND
	TLR4		PKN2		RSSA
	LY96		PRKDC		PML
Module 27	CCNA2		MK10		MK08
	CCNB1		RL11		MK09
	CCNE1		STK4		MK10
	CCNH		P53		MP2K7
	CDC20		VIME		RAGP1
	CDK2		TOPRS		RL3
	CDKN3		HIPK2		RS3
	CKS1		PIAS4		RS21
	DNMT1		NLK		SUMO3
	FLNA		TRIB3		SUMO2
	FLNB		SIN1		TOP1
	HDAC1		T53I1		TOP2A
	HES1		RICTR		TOP2B
	MARK3	Module 75	AMPH		UBC9
	MAT1		BIN1		SUMO1
	GA45B		ARF1		FOSL1
	NP1L4		CLH1		C8AP2
	RBBP7		DYN1		SAE2
	RBL1		HIP1		SAE1
	BRD2		SH3G1		RASF1
	SUV91		SH3G2		IKZF3
	TAF1		CLH2		JIP3

	TAF2		SNX4		CBLC
	TLE1		ADAM9		KLC2
	TLE2		SYNJ1		ITCH
	1433Z		PDC6I		TRI63
	CAF1B		PDCD6		DUS19
	HIST1H4I		EPN2		ATR
	H2B2E		GGA3		GA45A
	HIST1H3A		GGA1		FEN1
	HIST1H3D		SHLB1		MSH6
	HIST1H3C		PACS1		DNLI1
	HIST1H3E		APC		MSH2
	HIST1H3I		CADH5		MSH3
	HIST1H3G		CTBP1		PCNA
	HIST1H3J		CTNA1	Module 112	DPOD1
	HIST1H3H		CTNB1		DPOD2
	HIST1H3B		DSC1		POLH
	HIST1H4A		DSG1		UBE2A
	HIST1H4D		EVI1		UBE2B
	HIST1H4F		GSK3B		GA45G
	HIST1H4K		HIC1		APEX2
	HIST1H4J		PLAK		RAD18
	HIST1H4C	Module 76	PSN1		DPOD4
	HIST1H4H		PSN2		SETD8
	HIST1H4B		PTPRB		BCL3
	HIST1H4E		TF7L2		GFI1
	HIST1H4L		ZEB1		NFKB1
	HIST2H4A		ICAM5		NFKB2
	SMCA5		RUVB2		IKBA
	HAT1		MARE1	Module 113	IKBB
	CCNA1		KIFA3		IKBE
	H31		APH1A		TF65
	CCNB2		LEF1		RELB
	CCNE2		CNBP1		PIAS3
	TIF1B		BMAL1		IKBZ
	CBX1	Module 77	EPAS1		JAG1
	TLK2		VHL		SUH
	CBX3		CLOCK	Module 114	NOTC1
	CBX5		BAG1		SNW1
	BRD4	Module 78	HSP71		DLL1
	ASF1A		HSP71		SYF1
	REM1		AIFM1		COF1
	TBL1R		CCND1	Module 115	G3P
	CCNB3	Module 79	CCND2		LIS1
	HIST4H4		CCND3		PA1B2

	HIST2H4B		CDK4		PA1B3
Module 28	CDX2		CDK6		PGK1
	GFI1		CDN1A		UBA1
	MITF		CDN1B		HS105
	PAX6		CDN2D		DISC1
	PIAS3		CEBPB		NDEL1
	RX		ANM2	Module 116	PRKN2
	VSX2		RB		RBP2
Module 29	ARPC5		HMGA2		UB2G1
	ARPC4		RBBP9		UB2L3
	ARPC3	Module 80	BAD		SNCAP
	ARC1B		BAK		ARI2
	ARPC2		BAX	Module 117	ADA2A
Module 30	SLU7		BCL2		DRD2
	PRP8		B2LA1		NEK2
	U520		B2CL1		PP1A
	PRP6		B2CL2		PP1G
Module 31	DKC1		BID		IPP2
	TF2AA		BIK		PP1R8
	T2AG		GRID2		RGS2
	TF2H4		MCL1		STAU1
	TF3B		PDPK1		ASPP2
	PIN1		PK3C3		WBP11
	SPT4H		KAPCA		NEB1
	SPT5H		PTN4		NEB2
	TAF5		BECN1	Module 118	TLX1
	TAF6		B2L11		PP2AA
	TAF10		SIVA		PP2AB
	TAF11		1433T		2AAA
	TAF12		BBC3		2AAB
	TAF13		GOPC		2ABA
	TBP		BMF		2A5A
	SYMPK		DYL2	Module 119	M3K3
	RUVB1	Module 81	CAC1C		MP2K5
	EDF1		CALM		SQSTM
	TAF1C		CALM		SH22A
	TAF1B		CALM		M3K2
	TAF1A		GRB7	Module 120	ADRB3
	BTAF1		KCNN2		CRK
	SSU72		KCNQ2		DCC
	TAF9B		KCC1A		DOCK1
	TAF8		KKCC2		DOCK3
Module 32	RCAN1	Module 82	MTG16		FAK2
	PP2BA		ID1		ITB5

	CANB1		ID2		LCK
	CABP1		ID3		NEO1
	RCAN2		LYL1		NPHP1
	CABP2		MYOD1		FAK1
	CABP5		TAL1		PAXI
	KAP0		ITF2		SRC
	KAP2		TFE2		TGFI1
	AKAP4		HTF4		VINC
	AKAP5		TCF21		ADA15
Module 33	AKAP9		TWST1		BCAR1
	AKAP3		HAND2		3BP1
	AKAP2		BCR		ATR
	SP17		BTK		BLM
	ROP1A		CAV3		BRCA2
	DUS1		CBL	Module 121	RAD51
	DUS4		CBLB		RAD52
	DUS6		CD3E		RFA1
	ELK1		MPIP3		RFA2
	MKNK2		CRKL		R51A1
	MBP		CSF1R		NSF
	M3K1		DAG1		PLD1
	RASN		DYN2		SNP25
Module 34	MK01		DOK1		STX1A
	MK03		SRC8		STX3
	MP2K1		EPOR		STX4
	MP2K2		ERBB2		STX5
	PTPRR		ERBB3		STXB1
	RAF1	Module 83	FGR		STXB2
	KS6A1		VGFR1		STXB3
	MKNK1		FYB		VAMP1
	KS6A4		FYN		VAMP2
	PTN5		GAB1	Module 122	SYT1
	PSA1		GRB2		STX7
	PSA2		RPGF1		SNP23
	PSA3		HCK		SNAA
	PSA4		HCLS1		VAMP3
	PSA5		HNRPK		SNP29
Module 35	PSA6		SHIP1		GOSR1
	PSA7		IRS1		CPLX1
	PSB1		ITK		EHD1
	PSB2		KIT		STX12
	PSB3		LAMA2		EHD3
	PSB4		LAMA5		STX1B
	PSB5		LCP2		TRIM9

	PSB6		SH21A		STXB5
	PSB7		TAU		BIRC2
	PSB8		NCK1		BIRC3
	PSB9		NPHN		CD27
	PSB10		NTF3		TNR5
	PRP19		NTRK2		TNR9
	PRS4		NTRK3		IRAK1
	PRS7		CNRG		TNR3
	PRS6A		PK3CA		TNAP3
	PRS6B		P85A		TRAF1
	PRS8		PLCG1		TRAF2
	PRS10		PLK1	Module 123	TRAF3
	PSMD1		PTN6		TRAF5
	PSMD2		PTN11		TRAF6
	PSMD4		PTPRA		TNR4
	PSD7		RET		TNR11
	PSMD8		ITSN1		TBKB1
	PSMD9		SLAF1		TANK
Module 36	PSD10		SOS1		CIKS
	PSD11		SOS2		TBK1
	PSD12		STA5A		PELI2
	PSD13		KSYK		MAVS
	RD23A		TPD53		CADH1
	RD23B		VAV		RNF4
	XPC		WASP		SIAH1
	UBP14		WIPF1		UB2D2
	PSMD6		1433B		UB2D3
	PSDE		1433E	Module 124	APC10
	ADRM1		PODO		PUF60
	UCHL5		P55G		ANC2
	PAAF1		SYNJ2		APC11
	RAB4A		SKAP2		OTUB1
Module 37	KIF3B		WASL		RNF25
	CD2AP		HIP1R		AFAD
	RUFY1		CIP4	Module 125	SRBS2
	MYO5A		GRAP2		DLGP4
	RAB3A		M4K4		JAM1
	RB27A		GAB2		DC1I1
	RB27B		TM1L1		GLRB
Module 38	SYTL2		ARP3	Module 126	DYLT1
	MELPH		SF3B4		DYL1
	SYTL1		KHDR1		GEPH
	SYTL4		FRS2	Module 127	PPARG
	SYTL5		SF3B2		HDAC3

	RCC1		M4K1		NCOR2
	XPO2		AATF		HDAC5
	SYDC		LAT		PAK2
	EF1A1		BLNK		SPTA2
	IF5A1		SH3K1		SPTB2
	IMA1		ITSN2		SSR2
	IMB1		ERRFI	Module 128	ARHG7
	IMA2		DOK5		ARHG6
	IMA4		EP15R		SHAN2
	TNPO1		SHKB1		GIT1
	IPO5		BCAP		SHAN1
	RAN		CD2		SHRPN
	RANG	Module 84	CD48		TS101
Module 39	RBP2		CD2B2		STAM1
	RAGP1		RHG01		UBP8
	RL7		GDIR1	Module 129	HGS
	UB2E1		GDIR3		STAM2
	XPO1		C1QBP		STABP
	AIMP2		CDC42		VPS28
	NTF2		PAK1		KC1E
	UB2E3		PAK3	Module 130	DVL2
	IPO7		KPCI		BAG3
	NUP50		KPCZ		CLUS
	IMA7		RAC1		GNA12
	MOG1		1433G		HSP74
	IPO11	Module 85	MRCKA		HSP7C
	XPO5		WASF1		HS90A
	BMI1		ARHG6		HS90B
	BTF3		WASF2		PPID
	CSK21		BAIP2	Module 131	PPP5
	CSK22		IQGA2		PRIO
	CSK2B		PAR6A		SGTA
	E2F6		PARD3		SIM1
	IF2A		RHOJ		AIP
	IF2G		PAR6G		BAG2
Module 40	IF5		PAR6B		STIP1
	FHL1		BORG3		CDC37
	RING1		IKKA		CHRD1
	RING2	Module 86	IKKB		TNFL6
	LA		NEMO	Module 132	PACN2
	PCGF2		KEAP1		PACN3
	UBP7		ATF2		PACN1
	CBX4	Module 87	MK14	Module 133	MPRI
	IF2B		MK11		SORL

	AKA12		MP2K4		GGA2
	PCGF3		MAPK3		HDAC2
	RYBP		MKNK1		SAP30
	CBX7		KS6A5	Module 134	NCOR1
	CBX8		MAPK2		SIN3B
	LMBL2		JDP2		SIN3A
	PCGF6		APBA2		SDS3
	PCGF5		APBB1		VPS4B
	CBX2		APLP1		CHM2A
	PCGF1		APLP2	Module 135	CHMP3
	RS2		A4		CHMP6
	RS3		DAB1		CHM4B
Module 41	RS4X		KINH		AVR2B
	RS11		KLC1		REPS2
	ANM3		LDLR	Module 136	GDF11
	UB2R1	Module 88	LRP1		RBP1
	CKS1		M3K11		SYJ2B
	NEDD8		VLDLR		
	SKP1		M3K12		
	SKP2		LRP8		
	CUL5		JIP1		
	CUL3		APBA3		
	CUL1		SNX17		
	ULA1		APBB3		
	UBA3		JIP2		
Module 42	UBC12		APBA1		
	RBX2		AT2B4		
	RBX1		CAC1B		
	SUGT1		IRK12		
	FBXL6	Module 89	SDC2		
	DCNL1		CSKP		
	KCTD5		LIN7A		
	FBXW5		LIN7C		
	FBXW7		CP070		
	FBXL8		CNTP4		
	KDM2B		DVL1		
	SENP8		DVL3		
	MECP2		SMAD1		
	SMCA2		SMAD3		
	SMCA4	Module 91	SMAD4		
Module 43	SNF5		SMAD6		
	SMRC1		SMAD7		
	SMRC2		LAP2		
	ARI1A		SMUF1		

	ARI1B		KLH12		
Module 44	JAK1	Module 92	KLF6		
	KPCD		E2F1		
	STAT3		NECD		
	NDUAD		NFYA		
Module 45	PPM1B		NFYB		
	MP2K6		NFYC		
	M3K7		TNR16		
	TAB1		SP1		
	TAB2		TFDP1		
Module 46	ELOC		TFDP2		
	ELOB		MAGG1		
	CUL2	Module 93	EFNB1		
	SOCS1		PTN13		
	ZER1		NCK2		

T_Modules denotes index of Topological modules.

Supplementary table 2: Summary of GO function enrichment analysis of topological modules.

T_Module	Item Description	corr p-value	hit	total	GO_Category
Module 1	non-membrane-bounded organelle	5.34E-03	4	4	CC
Module 2	signaling pathway	1.08E-03	5	5	BP
	signal transmission	1.08E-03	5	5	BP
	signaling process	1.08E-03	5	5	BP
	regulation of biological process	4.63E-02	5	5	BP
Module 3	vesicle-mediated transport	2.90E-11	8	8	BP
	establishment of protein localization	2.01E-10	8	8	BP
	establishment of localization in cell	5.03E-10	8	8	BP
	cellular localization	1.21E-09	8	8	BP
	macromolecule localization	3.26E-09	8	8	BP
	transport	2.20E-06	8	8	BP
	establishment of localization	2.36E-06	8	8	BP
	cytoplasmic vesicle	3.15E-11	8	8	CC
	vesicle	4.17E-11	8	8	CC
	endomembrane system	1.02E-08	8	8	CC
	organelle membrane	8.20E-08	8	8	CC
	intracellular organelle part	1.38E-04	8	8	CC
	organelle part	1.48E-04	8	8	CC
	membrane part	8.17E-04	8	8	CC
	membrane	3.10E-03	8	8	CC
	membrane-bounded organelle	8.49E-03	8	8	CC
	intracellular organelle	1.76E-02	8	8	CC
	protein binding	3.22E-02	8	8	MF
Module 4	response to abiotic stimulus	3.44E-08	6	6	BP

	response to stress	1.24E-04	6	6	BP
Module 5	membrane	1.06E-03	11	11	CC
	protein binding	1.33E-02	11	11	MF
Module 6	anatomical structure morphogenesis	2.40E-05	6	6	BP
	signaling pathway	1.71E-04	6	6	BP
	anatomical structure development	4.50E-04	6	6	BP
	multicellular organismal development	7.00E-04	6	6	BP
	regulation of biological process	1.78E-02	6	6	BP
	protein binding	3.22E-02	6	6	MF
Module 7	protein complex biogenesis	1.79E-05	4	4	BP
	macromolecular complex subunit organization	5.81E-05	4	4	BP
	cellular component assembly	1.32E-04	4	4	BP
	cellular response to stimulus	1.70E-04	4	4	BP
	catabolic process	1.78E-04	4	4	BP
	response to stress	1.32E-03	4	4	BP
	biosynthetic process	1.33E-03	4	4	BP
	nitrogen compound metabolic process	2.67E-03	4	4	BP
	macromolecule metabolic process	1.56E-02	4	4	BP
	cellular metabolic process	2.81E-02	4	4	BP
	primary metabolic process	3.31E-02	4	4	BP
	organelle lumen	3.12E-04	4	4	CC
	protein complex	1.23E-03	4	4	CC
	intracellular organelle part	1.24E-02	4	4	CC
	organelle part	1.24E-02	4	4	CC
Module 8	protein complex	2.76E-03	4	4	CC
	nucleic acid binding	2.11E-02	4	5	MF
Module 9	cellular pigmentation	4.01E-07	3	4	BP
	vesicle-mediated transport	3.64E-03	3	4	BP
	establishment of localization in cell	7.83E-03	3	4	BP
	cellular localization	9.80E-03	3	4	BP
	organelle organization	2.02E-02	3	4	BP
Module 10	membrane-bounded organelle	9.68E-03	8	8	CC
	intracellular organelle	1.77E-02	8	8	CC
Module 11	multicellular organismal development	2.85E-02	4	4	BP
	transcription factor activity	1.21E-04	4	4	MF
	nucleic acid binding	5.56E-03	4	4	MF
Module 12	anatomical structure morphogenesis	1.67E-05	6	6	BP
	anatomical structure development	5.25E-04	6	6	BP
	multicellular organismal development	8.19E-04	6	6	BP
	ion binding	1.16E-03	6	6	MF
	protein binding	3.85E-02	6	6	MF
Module 13	signal transducer activity	4.15E-02	3	4	MF
Module 14	transferase activity	4.78E-04	4	4	MF

	nucleic acid binding	5.33E-03	4	4	MF
Module 15	protein complex biogenesis	3.98E-04	3	3	BP
	macromolecular complex subunit organization	9.68E-04	3	3	BP
	cellular component assembly	1.79E-03	3	3	BP
	biosynthetic process	8.10E-03	3	3	BP
	nitrogen compound metabolic process	1.35E-02	3	3	BP
	macromolecule metabolic process	4.74E-02	3	3	BP
	organelle lumen	3.33E-03	3	3	CC
	RNA polymerase II transcription factor activity	4.70E-05	3	3	MF
Module 16	cellular metabolic process	1.36E-06	21	24	BP
	primary metabolic process	4.07E-06	21	24	BP
	intracellular organelle	9.92E-04	21	24	CC
	intracellular part	1.72E-02	21	24	CC
	intracellular	2.85E-02	21	24	CC
Module 17	small molecule metabolic process	9.29E-06	6	6	BP
	biosynthetic process	4.07E-05	6	6	BP
	signal transmission	1.02E-04	6	6	BP
	signaling process	1.02E-04	6	6	BP
	cellular metabolic process	6.06E-03	6	6	BP
	primary metabolic process	8.20E-03	6	6	BP
	regulation of biological process	2.32E-02	6	6	BP
	protein binding	2.74E-02	6	6	MF
Module 18	regulation of biological process	1.76E-02	7	7	BP
Module 19	protein complex	1.02E-04	8	9	CC
	cell adhesion	1.57E-06	7	9	BP
	anatomical structure development	2.25E-03	7	9	BP
	multicellular organismal development	4.26E-03	7	9	BP
	protein binding	3.08E-02	8	9	MF
Module 20	extracellular matrix	5.46E-10	6	6	CC
	extracellular region part	2.61E-07	6	6	CC
Module 21	membrane-bounded organelle	3.27E-02	9	9	CC
	intracellular organelle	3.96E-02	9	9	CC
Module 22	nitrogen compound metabolic process	3.65E-09	11	11	BP
	macromolecule metabolic process	2.51E-06	11	11	BP
	cellular metabolic process	2.63E-05	11	11	BP
	primary metabolic process	4.81E-05	11	11	BP
	ribonucleoprotein complex	2.40E-16	11	11	CC
	organelle lumen	5.58E-11	11	11	CC
	intracellular organelle part	3.70E-06	11	11	CC
	organelle part	4.00E-06	11	11	CC
	membrane-bounded organelle	1.01E-03	11	11	CC
	intracellular organelle	3.14E-03	11	11	CC
	intracellular part	1.68E-02	11	11	CC

	intracellular	2.29E-02	11	11	CC
	protein binding	1.13E-02	11	11	MF
Module 23	organelle lumen	1.50E-16	17	17	CC
	intracellular organelle part	4.17E-09	17	17	CC
	organelle part	4.84E-09	17	17	CC
	membrane-bounded organelle	3.33E-05	17	17	CC
	intracellular organelle	1.78E-04	17	17	CC
	intracellular part	2.35E-03	17	17	CC
	intracellular	3.75E-03	17	17	CC
	protein binding	4.97E-05	17	17	MF
Module 24	signal release	9.79E-07	3	3	BP
	generation of a signal involved in cell-cell signaling	9.79E-07	3	3	BP
	secretion by cell	1.28E-05	3	3	BP
	cell-cell signaling	1.79E-04	3	3	BP
	cell communication	4.31E-04	3	3	BP
	establishment of localization in cell	4.77E-04	3	3	BP
	cellular localization	6.35E-04	3	3	BP
	system process	2.02E-03	3	3	BP
	regulation of biological quality	2.03E-03	3	3	BP
	signal transmission	4.95E-03	3	3	BP
	signaling process	4.95E-03	3	3	BP
	transport	7.90E-03	3	3	BP
	establishment of localization	7.90E-03	3	3	BP
	synaptic vesicle	6.25E-06	3	3	CC
	synapse part	5.38E-05	3	3	CC
	cytoplasmic vesicle	3.18E-04	3	3	CC
	vesicle	3.30E-04	3	3	CC
	nucleoside binding	1.42E-02	3	4	MF
Module 25	regulation of biological process	1.57E-02	7	7	BP
	membrane-bounded organelle	3.30E-02	7	7	CC
	intracellular organelle	4.30E-02	7	7	CC
	nucleic acid binding	6.21E-05	7	7	MF
Module 26	regulation of biological process	4.03E-02	5	5	BP
Module 27	intracellular organelle	1.42E-07	41	45	CC
	intracellular part	3.73E-06	42	45	CC
	intracellular	1.10E-05	42	45	CC
	cell part	4.75E-02	43	45	CC
	protein binding	5.59E-08	42	45	MF
Module 28	regulation of biological process	1.75E-02	7	7	BP
	nucleic acid binding	2.13E-04	7	7	MF
Module 29	non-membrane-bounded organelle	3.70E-04	5	5	CC
	protein complex	4.54E-04	5	5	CC
	intracellular organelle part	6.98E-03	5	5	CC

	organelle part	6.98E-03	5	5	CC
Module 30	nitrogen compound metabolic process	2.05E-03	4	4	BP
	macromolecule metabolic process	1.59E-02	4	4	BP
	cellular metabolic process	3.56E-02	4	4	BP
	primary metabolic process	4.35E-02	4	4	BP
	ribonucleoprotein complex	6.38E-06	4	4	CC
	intracellular organelle part	1.75E-02	4	4	CC
	organelle part	1.75E-02	4	4	CC
Module 31	membrane-bounded organelle	1.76E-07	25	25	CC
	intracellular organelle	2.46E-06	25	25	CC
	intracellular part	1.18E-04	25	25	CC
	intracellular	2.51E-04	25	25	CC
Module 32	signal transmission	7.09E-03	5	7	BP
	signaling process	7.09E-03	5	7	BP
	ion binding	4.58E-02	5	7	MF
Module 33	signal transmission	5.21E-04	7	8	BP
	signaling process	5.21E-04	7	8	BP
Module 34	regulation of biological process	8.75E-05	16	18	BP
	protein binding	1.51E-03	16	18	MF
Module 35	macromolecule metabolic process	3.32E-10	18	18	BP
	cellular metabolic process	1.53E-08	18	18	BP
	primary metabolic process	4.30E-08	18	18	BP
	regulation of biological process	1.97E-06	18	18	BP
	protein complex	4.15E-14	18	18	CC
	membrane-bounded organelle	2.54E-05	18	18	CC
	intracellular organelle	1.54E-04	18	18	CC
	intracellular part	2.50E-03	18	18	CC
	intracellular	4.10E-03	18	18	CC
Module 36	intracellular part	3.41E-04	25	25	CC
	intracellular	6.48E-04	25	25	CC
	protein binding	3.53E-06	25	25	MF
Module 38	transport	3.04E-06	9	9	BP
	establishment of localization	3.17E-06	9	9	BP
Module 39	intracellular part	1.18E-04	27	27	CC
	intracellular	2.30E-04	27	27	CC
Module 40	intracellular part	2.20E-04	25	25	CC
	intracellular	4.03E-04	25	25	CC
	protein binding	1.94E-05	26	27	MF
Module 41	biosynthetic process	1.47E-02	4	5	BP
	ribonucleoprotein complex	2.64E-05	4	5	CC
	non-membrane-bounded organelle	9.19E-03	4	5	CC
	structural constituent of ribosome	2.25E-06	4	5	MF
	nucleic acid binding	2.93E-02	4	5	MF
	macromolecule metabolic process	4.96E-02	4	5	BP

Module 42	protein binding	1.16E-04	20	22	MF
Module 43	regulation of biological process	4.01E-03	8	8	BP
	intracellular organelle part	2.40E-04	8	8	CC
	organelle part	2.40E-04	8	8	CC
	membrane-bounded organelle	9.17E-03	8	8	CC
	intracellular organelle	1.59E-02	8	8	CC
	intracellular part	4.85E-02	8	8	CC
	protein binding	1.60E-02	8	8	MF
Module 44	regulation of biological process	5.11E-02	4	4	BP
Module 45	protein binding	4.45E-02	5	5	MF
Module 47	membrane part	1.76E-05	13	13	CC
	membrane	1.52E-04	13	13	CC
	protein binding	7.01E-03	13	13	MF
Module 48	ligase activity	5.44E-07	5	5	MF
Module 49	macromolecule metabolic process	1.31E-03	7	7	BP
	cellular metabolic process	4.96E-03	7	7	BP
	primary metabolic process	7.13E-03	7	7	BP
	protein binding	5.41E-02	8	9	MF
Module 50	establishment of protein localization	2.24E-06	6	6	BP
	macromolecule localization	1.04E-05	6	6	BP
	transport	5.89E-04	6	6	BP
	establishment of localization	5.89E-04	6	6	BP
Module 51	translational initiation	9.05E-27	10	10	BP
	biosynthetic process	9.29E-09	10	10	BP
	macromolecule metabolic process	1.58E-05	10	10	BP
	cellular metabolic process	1.20E-04	10	10	BP
	primary metabolic process	2.01E-04	10	10	BP
	protein complex	9.76E-08	10	10	CC
	protein binding	7.00E-03	10	10	MF
Module 52	regulation of biological process	4.84E-02	5	5	BP
Module 54	cellular developmental process	2.71E-06	7	7	BP
	anatomical structure development	2.97E-05	7	7	BP
	multicellular organismal development	5.88E-05	7	7	BP
	macromolecule metabolic process	3.53E-04	7	7	BP
	cellular metabolic process	1.35E-03	7	7	BP
	primary metabolic process	1.99E-03	7	7	BP
	regulation of biological process	7.34E-03	7	7	BP
	protein binding	3.83E-02	7	7	MF
Module 55	protein binding	3.94E-02	5	5	MF
Module 56	protein binding	2.72E-02	8	8	MF
Module 57	extracellular structure organization	4.04E-07	4	4	BP
	cell adhesion	7.41E-05	4	4	BP
	system process	9.65E-04	4	4	BP
Module 58	regulation of biological process	4.98E-02	5	5	BP

	protein binding	4.73E-02	5	5	MF
Module 60	protein binding	1.90E-02	8	8	MF
Module 61	protein binding	3.54E-02	8	8	MF
Module 62	nitrogen compound metabolic process	2.55E-04	5	5	BP
	macromolecule metabolic process	4.53E-03	5	5	BP
	cellular metabolic process	1.18E-02	5	5	BP
	primary metabolic process	1.49E-02	5	5	BP
	ribonucleoprotein complex	3.13E-07	5	5	CC
	intracellular organelle part	3.80E-03	5	5	CC
	organelle part	3.88E-03	5	5	CC
	membrane-bounded organelle	4.48E-02	5	5	CC
Module 63	cell adhesion	9.21E-04	3	3	BP
	reproductive process	1.24E-03	3	3	BP
	anatomical structure development	1.35E-02	3	3	BP
Module 64	macromolecule metabolic process	4.20E-03	6	6	BP
	cellular metabolic process	1.16E-02	6	6	BP
	primary metabolic process	1.54E-02	6	6	BP
Module 65	cellular membrane organization	5.33E-04	3	3	BP
	membrane organization	5.33E-04	3	3	BP
	vesicle-mediated transport	1.30E-03	3	3	BP
	transport	2.20E-02	3	3	BP
	establishment of localization	2.20E-02	3	3	BP
	endomembrane system	9.89E-03	3	3	CC
Module 66	cellular response to stimulus	1.32E-03	4	5	BP
	response to stress	5.40E-03	4	5	BP
	biosynthetic process	5.40E-03	4	5	BP
	nitrogen compound metabolic process	9.19E-03	4	5	BP
	macromolecule metabolic process	4.99E-02	4	5	BP
	transferase activity	1.40E-03	4	5	MF
	nucleic acid binding	1.54E-02	4	5	MF
	ion binding	2.14E-02	4	5	MF
Module 67	nitrogen compound metabolic process	7.57E-06	7	7	BP
	macromolecule metabolic process	4.01E-04	7	7	BP
	cellular metabolic process	1.70E-03	7	7	BP
	primary metabolic process	2.10E-03	7	7	BP
	ribonucleoprotein complex	4.70E-10	7	7	CC
	membrane-bounded organelle	1.90E-02	7	7	CC
	intracellular organelle	3.38E-02	7	7	CC
	nucleic acid binding	4.57E-05	7	7	MF
	protein binding	2.22E-02	7	7	MF
Module 68	biosynthetic process	5.66E-07	8	8	BP
	nitrogen compound metabolic process	2.26E-06	8	8	BP
	macromolecule metabolic process	2.03E-04	8	8	BP
	cellular metabolic process	1.05E-03	8	8	BP

	primary metabolic process	1.61E-03	8	8	BP
	membrane-bounded organelle	8.31E-03	8	8	CC
	intracellular organelle	1.54E-02	8	8	CC
Module 69	organelle lumen	3.44E-05	5	5	CC
	non-membrane-bounded organelle	1.44E-04	5	5	CC
	intracellular organelle part	4.05E-03	5	5	CC
	organelle part	4.07E-03	5	5	CC
	membrane-bounded organelle	4.84E-02	5	5	CC
	nucleic acid binding	1.17E-03	5	5	MF
Module 71	protein binding	3.51E-02	8	8	MF
Module 72	cytoplasmic vesicle	9.78E-14	10	10	CC
	vesicle	1.36E-13	10	10	CC
	membrane-bounded organelle	2.09E-03	10	10	MF
	intracellular organelle	5.90E-03	10	10	MF
	intracellular part	2.67E-02	10	10	MF
	intracellular	3.52E-02	10	10	MF
Module 73	anatomical structure development	9.88E-03	4	4	BP
	multicellular organismal development	1.18E-02	4	4	BP
	transcription factor activity	1.11E-02	3	4	MF
	nucleic acid binding	4.62E-02	3	4	MF
Module 74	regulation of biological process	9.39E-05	26	30	BP
	membrane-bounded organelle	1.52E-05	27	29	CC
	intracellular organelle	1.77E-05	28	29	CC
	intracellular part	7.30E-05	29	29	CC
	intracellular	1.70E-04	29	29	CC
	protein binding	8.28E-06	28	29	MF
Module 75	intracellular part	1.27E-02	18	19	CC
	intracellular	1.98E-02	18	19	CC
	protein binding	3.28E-03	18	19	MF
Module 76	protein binding	1.04E-05	23	23	MF
Module 77	protein complex	4.54E-03	4	4	CC
Module 79	regulation of biological process	3.93E-04	13	13	BP
	membrane-bounded organelle	1.86E-03	13	13	CC
	intracellular organelle	5.25E-03	13	13	CC
	intracellular part	2.05E-02	13	13	CC
	intracellular	2.18E-02	13	13	CC
	protein binding	1.37E-03	13	13	MF
Module 80	intracellular part	7.86E-04	23	23	CC
	intracellular	1.52E-03	23	23	CC
	protein binding	2.32E-05	23	23	MF
Module 81	signal transmission	3.96E-02	4	7	BP
	signaling process	3.96E-02	4	7	BP
Module 82	membrane-bounded organelle	2.05E-03	13	13	CC
	intracellular organelle	4.36E-03	13	13	CC

	intracellular part	2.61E-02	13	13	CC
	intracellular	3.51E-02	13	13	CC
Module 83	intracellular	2.06E-02	70	84	CC
	protein binding	9.27E-17	84	88	MF
Module 85	intracellular	1.84E-03	22	22	CC
	intracellular part	7.74E-03	21	22	CC
	protein binding	1.02E-03	21	23	MF
Module 86	regulation of biological process	5.23E-02	4	4	BP
Module 87	regulation of biological process	3.89E-03	9	9	BP
Module 88	protein binding	9.31E-05	19	19	MF
Module 89	membrane	3.06E-03	10	10	CC
	protein binding	4.47E-02	10	10	MF
Module 90	membrane	6.22E-07	30	34	CC
	protein binding	2.80E-09	33	34	MF
	cell part	1.57E-02	34	34	CC
Module 91	signaling pathway	3.98E-07	10	10	BP
	protein binding	3.73E-03	10	10	MF
Module 92	regulation of biological process	2.48E-03	11	11	BP
	membrane-bounded organelle	2.38E-03	11	11	CC
	intracellular organelle	6.47E-03	11	11	CC
	intracellular part	2.96E-02	11	11	CC
	intracellular	3.99E-02	11	11	CC
Module 94	regulation of biological process	1.40E-03	11	11	BP
	membrane-bounded organelle	2.66E-03	11	11	CC
	intracellular organelle	7.23E-03	11	11	CC
	intracellular part	2.99E-02	11	11	CC
	intracellular	3.96E-02	11	11	CC
	protein binding	2.19E-03	11	11	MF
Module 96	regulation of biological process	1.82E-02	6	6	BP
Module 97	regulation of biological process	1.86E-02	7	7	BP
	membrane-bounded organelle	1.57E-02	7	7	CC
	intracellular organelle	2.82E-02	7	7	CC
	protein binding	2.99E-02	7	7	MF
Module 98	regulation of biological process	2.18E-02	6	6	BP
	protein binding	4.63E-02	6	6	MF
Module 99	establishment of protein localization	7.13E-08	7	7	BP
	macromolecule localization	4.81E-07	7	7	BP
	transport	1.28E-04	7	7	BP
	establishment of localization	1.28E-04	7	7	BP
	protein binding	2.27E-02	7	7	MF
Module 100	signaling pathway	2.51E-09	12	13	BP
	membrane	4.90E-03	11	13	CC
	signal transducer activity	3.17E-09	12	13	MF
Module 101	signaling pathway	6.93E-03	4	4	BP

Module 102	regulation of biological process	2.04E-02	13	15	BP
	membrane	2.47E-03	13	15	CC
	membrane part	2.47E-03	12	15	CC
Module 103	protein binding	2.39E-02	11	12	MF
Module 104	non-membrane-bounded organelle	1.05E-04	6	6	CC
	intracellular organelle part	1.95E-03	6	6	CC
	organelle part	1.99E-03	6	6	CC
	membrane-bounded organelle	2.54E-02	6	6	CC
	intracellular organelle	4.55E-02	6	6	CC
Module 105	anatomical structure development	2.18E-02	4	4	BP
	multicellular organismal development	2.18E-02	4	4	BP
Module 106	regulation of biological process	1.61E-03	11	11	BP
Module 107	macromolecule metabolic process	3.76E-02	5	6	BP
Module 110	protein binding	1.08E-05	21	21	MF
Module 111	intracellular part	2.78E-06	40	40	CC
	intracellular	6.22E-06	40	40	CC
Module 112	cellular response to stimulus	8.05E-19	17	18	BP
	response to stress	1.08E-14	17	18	BP
	nitrogen compound metabolic process	2.71E-13	17	18	BP
	macromolecule metabolic process	3.57E-09	17	18	BP
	cellular metabolic process	9.46E-08	17	18	BP
	primary metabolic process	2.17E-07	17	18	BP
	membrane-bounded organelle	1.83E-04	17	18	CC
	intracellular organelle	8.96E-04	17	18	CC
	intracellular part	7.04E-03	17	18	CC
	intracellular	1.02E-02	17	18	CC
Module 113	regulation of biological process	1.02E-03	11	11	BP
	membrane-bounded organelle	4.37E-03	11	11	CC
	intracellular organelle	8.95E-03	11	11	CC
	intracellular part	4.24E-02	11	11	CC
	intracellular	4.93E-02	11	11	CC
Module 114	protein binding	2.28E-02	6	6	MF
Module 115	primary metabolic process	3.03E-02	7	10	BP
Module 116	cellular metabolic process	9.75E-03	6	6	BP
	primary metabolic process	1.11E-02	6	6	BP
Module 117	protein binding	2.11E-03	13	13	MF
Module 118	protein binding	3.00E-02	7	7	MF
Module 119	protein binding	4.78E-02	5	5	MF
Module 120	protein binding	2.05E-04	18	18	MF
Module 121	cellular response to stimulus	1.49E-08	8	8	BP
	response to stress	1.36E-06	8	8	BP
	nitrogen compound metabolic process	5.26E-06	8	8	BP
	macromolecule metabolic process	3.15E-04	8	8	BP
	cellular metabolic process	1.51E-03	8	8	BP

	primary metabolic process	2.19E-03	8	8	BP
	membrane-bounded organelle	9.45E-03	8	8	CC
	intracellular organelle	1.89E-02	8	8	CC
	nucleic acid binding	6.24E-05	8	8	MF
	protein binding	2.00E-02	8	8	MF
Module 122	protein binding	1.33E-06	26	26	MF
Module 123	protein binding	1.04E-04	20	20	MF
Module 124	macromolecule metabolic process	1.35E-05	11	11	BP
	primary metabolic process	1.87E-04	11	11	BP
	protein binding	5.95E-03	11	11	MF
Module 126	transport	3.59E-02	4	5	BP
	establishment of localization	3.59E-02	4	5	BP
	non-membrane-bounded organelle	1.18E-02	4	5	CC
	protein complex	1.27E-02	4	5	CC
Module 127	anatomical structure development	6.83E-03	4	4	BP
	multicellular organismal development	8.59E-03	4	4	BP
	transcription repressor activity	6.56E-06	4	4	MF
Module 128	protein binding	2.65E-02	9	10	MF
Module 129	membrane	1.00E-02	7	7	CC
	membrane-bounded organelle	2.19E-02	7	7	CC
	intracellular organelle	4.24E-02	7	7	CC
Module 131	protein binding	1.57E-03	15	16	MF
Module 132	cytoplasmic vesicle	3.11E-03	3	4	CC
	vesicle	3.11E-03	3	4	CC
	organelle organization	1.98E-02	3	4	BP
Module 133	vesicle-mediated transport	2.69E-03	3	3	BP
Module 134	regulation of biological process	3.52E-02	6	6	BP
Module 135	establishment of protein localization	1.60E-05	5	5	BP
	macromolecule localization	5.11E-05	5	5	BP
	transport	2.46E-03	5	5	BP
	establishment of localization	2.46E-03	5	5	BP
	organelle membrane	9.36E-05	5	5	CC
	intracellular organelle part	7.49E-03	5	5	CC
	organelle part	7.49E-03	5	5	CC
	membrane	4.49E-02	5	5	CC

T_Module denotes the index of topological modules.

Supplementary table 3: Summary of disease modules.

D_M	Gene_name	Disease_name	Disease_category	T_M
1	COPA	Alzheimer`s disease (age of onset)	Psychiatric	3
	COPB2	Schizophrenia(age at onset)	Psychiatric	3
	COPZ1	Platelet counts	Psychiatric	3
2	CRYAB	Myopathy, cardioskeletal, desmin-related, with cataract, 608810 (3)	Neurological	4
	HSPB1	Charcot-Marie-Tooth disease, axonal, type 2F, 606595 (3)	Neurological	4

	HSPB1	Neuropathy, distal hereditary motor, type IIB, 608634 (3)	Neurological	4
	HSPB8	Charcot-Marie-Tooth disease, axonal, type 2L, 608673 (3)	Neurological	4
	HSPB8	Neuropathy, distal hereditary motor, type IIA, 158590 (3)	Neurological	4
	SCN5A	Brugada syndrome 1, 601144	Cardiovascular	5
	SCN5A	Cardiomyopathy, dilated, 1E, 601154	Cardiovascular	5
	DMD	Cardiomyopathy, dilated, 3B, 302045	Cardiovascular	5
	SCN5A	Heart block, nonprogressive, 113900	Cardiovascular	5
	SCN5A	Heart block, progressive, type IA, 113900	Cardiovascular	5
	DTNA	Left ventricular noncompaction with congenital heart defects, 606617	Cardiovascular	5
	DTNA	Left ventricular noncompaction, familial isolated, 1, 604169	Cardiovascular	5
	SCN5A	Long QT syndrome-3, 603830	Cardiovascular	5
	SCN5A	Sick sinus syndrome 1, 608567	Cardiovascular	5
	SCN5A	Ventricular fibrillation, idiopathic, 603829	Cardiovascular	5
	SCN5A	Electrocardiographic conduction measures	Cardiovascular	5
	SCN5A	Electrocardiographic traits	Cardiovascular	5
	SNTB2	HDL particle features	Cardiovascular	5
	SCN5A	PR interval	Cardiovascular	5
	SCN5A	QRS duration	Cardiovascular	5
	SCN5A	QT interval	Cardiovascular	5
	DMD	Response to statin treatment (atorvastatin), change in cholesterol levels	Cardiovascular	5
3	SCN5A	Ventricular conduction	Cardiovascular	5
	SNTB1	Hypertension	Cardiovascular	5
	MAST2	Calcium levels	Cardiovascular	5
	DTNA	Coronary Artery Disease	Cardiovascular	5
	DMD	Coronary heart disease	Cardiovascular	5
	SNTB1	Glucose levels	Cardiovascular	5
	DMD	Type 2 diabetes	Endocrine	5
	DTNA	Type 2 diabetes	Endocrine	5
	NOS1	Type 2 diabetes	Endocrine	5
4	SNTA1	Type 2 diabetes	Endocrine	5
	MAST2	Type 2 diabetes	Endocrine	5
	VAC14	Type 2 diabetes	Endocrine	5
	SNTB1	Glucose levels	Endocrine	5
	EGLN	Hereditary hemorrhagic telangiectasia-1, 187300 (3)	Bone	6
	TGFR1	Loeys-Dietz syndrome, type 1A, 609129 (3)	Bone	6
	TGFR1	Loeys-Dietz syndrome, type 2A, 608967 (3)	Bone	6
5	TGFR2	Loeys-Dietz syndrome, type 1B, 610168 (3)	Bone	6
	TGFR2	Loeys-Dietz syndrome, type 2B, 610380 (3)	Bone	6
	TGFB1	Coronary heart disease	Bone	6
	MEIS1	Aspirin exacerbated respiratory disease in asthmatics	Respiratory	11
6	MEIS2	Acute lung injury	Respiratory	11
	MEIS2	Lung function (forced expiratory volume in 1 second)	Respiratory	11
	MEIS2	Lung function (forced vital capacity)	Respiratory	11
	PBX1	Acute lung injury	Respiratory	11

	MEIS2	Response to taxane treatment (placlitaxel)	Respiratory	11
	PBX1	Response to platinum-based chemotherapy in non-small-cell lung cancer	Respiratory	11
7	MEIS1	Restless legs syndrome	Neurological	11
	PBX1	Parkinson`s disease	Neurological	11
	MEIS2	Taste perception	Neurological	11
	PBX1	Taste perception	Neurological	11
	MEIS2	Response to antipsychotic treatment	Neurological	11
	MEIS2	White matter integrity	Neurological	11
	MEIS1	Alzheimer`s disease (late onset)	Neurological	11
	PBX1	Amyotrophic Lateral Sclerosis	Neurological	11
	PBX1	Body mass index	Neurological	11
	PBX1	Body mass (lean)	Neurological	11
8	MEIS1	Type 2 diabetes	Endocrine	11
	MEIS2	Insulin resistance	Endocrine	11
	PBX1	Diabetic retinopathy	Endocrine	11
9	MEIS1	PR interval	Cardiovascular	11
	PBX1	Response to statin treatment (atorvastatin), change in cholesterol levels	Cardiovascular	11
	PBX1	Blood pressure, CVD RF and other traits (body mass index (BMI), waist:hip ratio, renin activity in plasma, aldosterone concentration in plasma, BNP levels in plasma, alcohol consumption)	Cardiovascular	11
	MEIS1	Blood pressure, CVD RF and other traits (body mass index (BMI), waist:hip ratio, renin activity in plasma, aldosterone concentration in plasma, BNP levels in plasma, alcohol consumption)	Cardiovascular	11
	MEIS2	Eosinophil counts	Cardiovascular	11
	PBX1	Coronary Artery Disease	Cardiovascular	11
10	MEIS2	Response to antipsychotic treatment	Psychiatric	11
	MEIS2	White matter integrity	Psychiatric	11
	PBX1	Nicotine smoking	Psychiatric	11
	PBX1	Coronary Artery Disease	Psychiatric	11
	MEIS1	Alzheimer`s disease (late onset)	Psychiatric	11
	PBX1	Amyotrophic Lateral Sclerosis	Psychiatric	11
	MEIS2	Eosinophil counts	Psychiatric	11
	PBX1	Parkinson`s disease	Psychiatric	11
11	GLI3	Hypothalamic hamartomas, somatic, 241800	Neurological	12
	GLI1	Personality dimensions	Neurological	12
	SKI	Cognitive test performance	Neurological	12
	ZIC2	Holoprosencephaly-5, 609637	Neurological	12
	GLI3	Acrocallosal syndrome, 200990	Neurological	12
12	GLI3	Heart Failure	Cardiovascular	12
	SKI	Blood pressure, CVD RF and other traits	Cardiovascular	12
	ZIC3	Heterotaxy, X-linked visceral, 306955	Cardiovascular	12
	GLI3	Coronary heart disease	Cardiovascular	12
13	ZIC2	Holoprosencephaly-5, 609637	Endocrine	12
	ZIC3	Heterotaxy, X-linked visceral, 306955	Endocrine	12
	GLI3	Type 2 diabetes	Endocrine	12
	GLI3	Polycystic ovary syndrome	Endocrine	12

	CDK7	Alzheimer`s disease	Neurological	16
	CDK8	Narcolepsy	Neurological	16
	RNF11	Schizophrenia (cytomegalovirus infection interaction)	Neurological	16
14	TCEA1	Amyotrophic Lateral Sclerosis	Neurological	16
	UBA1	Spinal muscular atrophy, X-linked 2, infantile, 301830	Neurological	16
	NEDD4	Parkinson`s disease (motor and cognition)	Neurological	16
	NEDD4	Brain connectivity	Neurological	16
	CSTF3	Taste perception	Neurological	16
	WBP2	White matter hyperintensity burden	Neurological	16
	THA	Pituitary adenoma, nonfunctioning	Endocrine	18
	THB	Thyroid hormone resistance, 188570	Endocrine	18
15	TIF1A	Thyroid carcinoma, papillary, 188550	Endocrine	18
	THB	Thyroid hormone resistance, autosomal recessive, 274300	Endocrine	18
	THB	Thyroid hromone resistance, selective pituitary, 145650	Endocrine	18
	NRIP1	Type 1 diabetes	Endocrine	18
	RXRA	Urinary metabolites	Endocrine	18
	NRIP1	Major depressive disorder	Psychiatric	18
	RXRA	Amyotrophic Lateral Sclerosis	Psychiatric	18
	NRIP1	Cognitive performance	Psychiatric	18
	RXRA	Intelligence	Psychiatric	18
16	MED25	Alcohol and nictotine co-dependence	Psychiatric	18
	LAMC1	IgE levels in asthmatics	Respiratory	20
	NID1	Lung function	Respiratory	20
17	NID2	Lung function	Respiratory	20
	NID2	Pulmonary function	Respiratory	20
	LAMC1	Smoking cessation	Respiratory	20
	FBLN2	Schizophrenia	Neurological	20
18	FBLN2	Alzheimer`s disease	Neurological	20
	FBLN2	Partial epilepsies	Neurological	20
	LAMB1	Personality dimensions	Neurological	20
	MYCL1	Schizophrenia	Neurological	21
	MYCN	Microcephaly and digital abnormalities with normal intelligence, 602585	Neurological	21
19	SMC3	Cornelia de Lange syndrome 3, 610759	Neurological	21
	MXI1	Normalized brain volume	Neurological	21
	MXI1	Multiple myeloma	Neurological	21
	MXI1	Neurofibrosarcoma	Neurological	21
	MXI1	Multiple myeloma	Cancer	21
	MXI1	Neurofibrosarcoma	Cancer	21
20	MXI1	Prostate cancer, susceptibility to, 176807	Cancer	21
	MYC	Burkitt lymphoma, 113970	Cancer	21
	MYCL1	Response to taxane treatment (placlitaxel)	Cancer	21
21	MCM6	Depression (quantitative trait)	Psychiatric	23
	ORC4	Sudden cardiac arrest	Psychiatric	23
	MCM5	Coronary heart disease	Psychiatric	23

	MCM3	Alzheimer`s disease	Psychiatric	23
	ORC5	Amyotrophic Lateral Sclerosis	Psychiatric	23
	ORC4	Parkinson`s disease	Psychiatric	23
22	ORC4	Sudden cardiac arrest	Cardiovascular	23
	MCM5	Coronary heart disease	Cardiovascular	23
	MCM2	Health and aging, CVD and cancer age of onset	Cardiovascular	23
	CDC6	Birth weight	Cardiovascular	23
	MCM6	Lipid levels	Cardiovascular	23
23	CAPON	QT interval, modifier of, 610141	Cardiovascular	24
	SYN3	Response to statin treatment (atorvastatin), change in cholesterol levels	Cardiovascular	24
	SYN2	Blood pressure, CVD RF and other traits	Cardiovascular	24
	SYN3	Eosinophil counts	Cardiovascular	24
	SYN3	Glucose levels	Cardiovascular	24
	SYN3	Serum albumin level	Cardiovascular	24
	SYN3	C-reactive protein	Cardiovascular	24
24	SYN3	Eosinophil counts	Psychiatric	24
	SYN3	C-reactive protein	Psychiatric	24
	SYN3	Major depressive disorder (broad)	Psychiatric	24
	SYN3	Suicide attempts in bipolar disorder	Psychiatric	24
	SYN2	White matter integrity	Psychiatric	24
	SYN3	White matter integrity	Psychiatric	24
	SYN3	Response to cholinesterase inhibitors in Alzheimer`s disease	Psychiatric	24
	SYN1	Amyotrophic Lateral Sclerosis	Psychiatric	24
	SYN3	Cognitive impairment induced by topiramate	Psychiatric	24
	SYN3	Cognitive test performance	Psychiatric	24
	SYN2	Schizophrenia, susceptibility to, 181500	Psychiatric	24
25	CCNA2	Red blood cell traits	Hematological	27
	FLNB	Adverse response to chemotherapy (neutropenia/leucopenia) (carboplatin)	Hematological	27
	FLNB	Adverse response to chemotherapy (neutropenia/leucopenia) (paclitaxel + carboplatin)	Hematological	27
	BRD2	Adverse response to chemotherapy (neutropenia/leucopenia) (cisplatin)	Hematological	27
	HIST1H3B	Red blood cell traits	Hematological	27
	HIST1H4C	Red blood cell traits	Hematological	27
	HIST1H4B	Other erythrocyte phenotypes	Hematological	27
	HIST1H4B	Red blood cell traits	Hematological	27
	DNMT1	Atopy	Hematological	27
	DNMT1	Thiazide-induced adverse metabolic effects in hypertensive patients	Hematological	27
	TLE1	Blood Pressure and Arterial Stiffness	Hematological	27
	TLE1	Blood pressure, CVD RF and other traits	Hematological	27
	CDC20	Platelet counts	Hematological	27
	FLNA	Heterotopia, periventricular nodular, with frontometaphyseal dysplasia, 300049	Hematological	27
	FLNA	Heterotopia, periventricular, 300049	Hematological	27
	FLNB	Lymphocyte counts	Hematological	27
26	FLNB	Boomerang dysplasia, 112310	Bone	27
	FLNB	Larsen syndrome, 150250	Bone	27

	FLNB	Spondylocarpotarsal synostosis syndrome, 272460	Bone	27
	MARK3	Bone mineral density (spine)	Bone	27
	MARK3	Bone mineral density	Bone	27
	HIST1H3B	Uric acid levels	Bone	27
	HIST1H3B	Urate levels	Bone	27
	HIST1H4C	Urate levels	Bone	27
	HIST1H4B	Urate levels	Bone	27
	BRD2	Rheumatoid arthritis	Bone	27
	FLNA	Heterotopia, periventricular, ED variant, 300537	Bone	27
	FLNB	Atelosteogenesis, type III, 108721	Bone	27
	FLNB	Atelostogenesis, type I, 108720	Bone	27
	FLNA	Otopalatodigital syndrome, type I, 311300	Bone	27
	FLNA	Otopalatodigital syndrome, type II, 304120	Bone	27
	FLNA	Frontometaphyseal dysplasia, 304120	Bone	27
	FLNA	Melnick-Needles syndrome, 309350	Bone	27
	CDKN3	Electrocardiographic traits and heart rate variability	Cardiovascular	27
	TLE1	Aortic root size	Cardiovascular	27
	FLNB	Myocardial Infarction	Cardiovascular	27
	TLE1	Tonometry	Cardiovascular	27
	DNMT1	Thiazide-induced adverse metabolic effects in hypertensive patients	Cardiovascular	27
	TLE1	Blood Pressure and Arterial Stiffness	Cardiovascular	27
	TLE1	Blood pressure, CVD RF and other traits	Cardiovascular	27
	FLNA	Heterotopia, periventricular, ED variant, 300537	Cardiovascular	27
	CDC20	Platelet counts	Cardiovascular	27
	TLE2	ldl cholesterol	Cardiovascular	27
	TLE2	Cholesterol	Cardiovascular	27
27	FLNB	Lymphocyte counts	Cardiovascular	27
	CCNE1	Type 2 diabetes	Endocrine	27
	CDK2	Polycystic ovary syndrome	Endocrine	27
28	BRD2	Type 1 diabetes	Endocrine	27
	TLE1	Type 2 diabetes	Endocrine	27
	TLE2	Type 2 diabetes	Endocrine	27
	CCNH	Response to lithium treatment in bipolar disorder	Psychiatric	27
	FLNB	Suicide attempts in bipolar disorder	Psychiatric	27
29	FLNB	Eating disorders (purging via substances)	Psychiatric	27
	TAF2	Suicide attempts in bipolar disorder	Psychiatric	27
	HAT1	Suicide attempts in bipolar disorder	Psychiatric	27
	CBX1	Depression (quantitative trait)	Psychiatric	27
	TAF2	Tourette syndrome	Psychiatric	27
	CDK2	White matter integrity	Psychiatric	27
	CDC20	Platelet counts	Psychiatric	27
	TLE2	ldl cholesterol	Psychiatric	27
	TLE2	Amyotrophic Lateral Sclerosis	Psychiatric	27
	BRD2	Autism spectrum disorder, attention deficit-hyperactivity disorder, bipolar disorder, major depressive disorder, and schizophrenia (combined)	Psychiatric	27

	TLE2	Cholesterol	Psychiatric	27
	TLE2	Parkinson`s disease	Psychiatric	27
	DNMT1	Cognitive impairment induced by topiramate	Psychiatric	27
	CCNE1	Alcohol dependence	Psychiatric	27
	FLNB	Alcohol dependence	Psychiatric	27
	FLNB	Alcoholism	Psychiatric	27
30	FLNA	Heterotopia, periventricular nodular, with frontometaphyseal dysplasia, 300049	Neurological	27
	FLNA	Heterotopia, periventricular, 300049	Neurological	27
	FLNB	Atelosteogenesis, type III, 108721	Neurological	27
	FLNB	Atelostogenesis, type I, 108720	Neurological	27
	FLNA	Otopalatodigital syndrome, type I, 311300	Neurological	27
	FLNA	Otopalatodigital syndrome, type II, 304120	Neurological	27
	TAF1	Dystonia-Parkinsonism, X-linked, 314250	Neurological	27
	BRD2	Multiple sclerosis	Neurological	27
	TLE2	Parkinson`s disease	Neurological	27
	DNMT1	Cognitive impairment induced by topiramate	Neurological	27
	FLNA	Frontometaphyseal dysplasia, 304120	Neurological	27
	FLNA	Melnick-Needles syndrome, 309350	Neurological	27
	HIST1H4H	Taste perception	Neurological	27
	FLNA	Heterotopia, periventricular, ED variant, 300537	Neurological	27
	CDK2	White matter integrity	Neurological	27
	CDK2	White matter integrity	Neurological	27
	TLE2	Amyotrophic Lateral Sclerosis	Neurological	27
	BRD2	Autism spectrum disorder, attention deficit-hyperactivity disorder, bipolar disorder, major depressive disorder, and schizophrenia (combined)	Neurological	27
31	CDK2	Asthma	Respiratory	27
	CDK2	Pulmonary function	Respiratory	27
	TLE1	Acute lung injury	Respiratory	27
	FLNB	Lymphocyte counts	Respiratory	27
	BRD2	Rheumatoid arthritis	Respiratory	27
	TLE2	Lung adenocarcinoma	Respiratory	27
	REM1	Lung adenocarcinoma	Respiratory	27
32	MITF	Tietz syndrome, 103500	Ophthamological	28
	MITF	Waardenburg syndrome, type IIA, 193510	Ophthamological	28
	MITF	Waardenburg syndrome/ocular albinism, digenic, 103470	Ophthamological	28
	PAX6	Aniridia, 106210	Ophthamological	28
	PAX6	Cataract with late-onset corneal dystrophy, 604219	Ophthamological	28
	PAX6	Coloboma of optic nerve, 120430	Ophthamological	28
	PAX6	Coloboma, ocular, 120200	Ophthamological	28
	PAX6	Ectopia pupillae, 129750	Ophthamological	28
	PAX6	Foveal hyperplasia, 136520	Ophthamological	28
	PAX6	Keratitis, 148190	Ophthamological	28
	PAX6	Optic nerve hypoplasia, 165550	Ophthamological	28
	PAX6	Peters anomaly, 604229	Ophthamological	28
	PAX6	Myopia (severe)	Ophthamological	28

	RX	Microphthalmia, isolated 3, 611038	Ophthamological	28
	VSX2	Microphthalmia, isolated 2, 610093	Ophthamological	28
	VSX2	Microphthalmia, isolated, with coloboma 3, 610092	Ophthamological	28
	PIAS3	Contrast sensitivity	Neurological	28
	GFI1	Multiple sclerosis	Neurological	28
	PAX6	Peters anomaly, 604229	Neurological	28
	TAF1B	Adverse response to chemotherapy (neutropenia/leucopenia) (cisplatin)	Hematological	31
	TAF8	Adverse response to chemotherapy (neutropenia/leucopenia) (camptothecin)	Hematological	31
33	TAF8	Red blood cell traits	Hematological	31
	EDF1	Self-reported allergy	Hematological	31
	DKC1	Dyskeratosis congenita-1, 305000	Hematological	31
	DKC1	Hoyeraal-Hreidarsson syndrome, 300240	Hematological	31
	TAF1B	Lymphocyte counts	Hematological	31
	BTAF1	Bipolar disorder	Psychiatric	31
	BTAF1	Schizophrenia	Psychiatric	31
	TAF5	Brain structure	Psychiatric	31
	TAF1C	Autism	Psychiatric	31
	TAF11	Alcoholism (heaviness of drinking)	Psychiatric	31
	TAF1A	Alcohol dependence	Psychiatric	31
	TBP	Parkinson disease, 168600	Psychiatric	31
34	BTAF1	Personality dimensions	Psychiatric	31
	KAP0	Myxoma, intracardiac, 255960	Cardiovascular	33
35	AKAP9	Long QT syndrome-11, 611820	Cardiovascular	33
	AKAP2	Aortic root size	Cardiovascular	33
	AKAP5	Intracranial aneurysm	Cardiovascular	33
	AKAP3	Lymphocyte counts	Cardiovascular	33
	MBP	Type 2 diabetes	Endocrine	34
36	PTPRR	Insulin resistance	Endocrine	34
	MKNK1	Type 2 diabetes	Endocrine	34
	RAF1	Noonan syndrome 5, 611553	Endocrine	34
	RASN	Thyroid carcinoma, follicular, 188470	Endocrine	34
	MYO5A	Griscelli syndrome, type 1, 214450	Neurological	38
	RB27A	Griscelli syndrome, type 2, 607624	Neurological	38
37	MELPH	Griscelli syndrome, type 3, 609227	Neurological	38
	MYO5A	Schizophrenia	Neurological	38
	SYTL4	Amyotrophic Lateral Sclerosis	Neurological	38
	SYTL4	Cognitive impairment induced by topiramate	Neurological	38
	MYO5A	Prion diseases	Neurological	38
	RBP2	Type 2 diabetes	Endocrine	39
38	XPO1	Type 2 diabetes	Endocrine	39
	AIMP2	Type 2 diabetes	Endocrine	39
	IPO11	Type 2 diabetes	Endocrine	39
	XPO5	Type 2 diabetes	Endocrine	39
39	RING1	Coronary heart disease	Psychiatric	40

	PCGF3	Cognitive impairment induced by topiramate	Psychiatric	40
	PCGF3	Hemostatic factors and hematological phenotypes	Psychiatric	40
	PCGF3	Parkinson`s disease	Psychiatric	40
	PCGF3	Eating disorders	Psychiatric	40
	RYBP	Soluble levels of adhesion molecules	Psychiatric	40
	PCGF5	Platelet aggregation(pre- and post-aspirin)	Psychiatric	40
	CBX2	Schizophrenia	Psychiatric	40
40	FHL1	Myopathy, X-linked, with postural muscle atrophy, 300696	Neurological	40
	FHL1	Myopathy, reducing body, X-linked, severe early-onset, 300717	Neurological	40
	PCGF3	Cognitive impairment induced by topiramate	Neurological	40
	PCGF3	Hemostatic factors and hematological phenotypes	Neurological	40
	PCGF3	Parkinson`s disease	Neurological	40
	CBX7	Multiple myeloma	Neurological	40
	PCGF6	White matter hyperintensity burden	Neurological	40
	CBX2	Schizophrenia	Neurological	40
41	CUL5	Functional impairment in major depressive disorder, bipolar disorder and schizophrenia	Psychiatric	42
	UBA3	Coronary heart disease	Psychiatric	42
	FBXW7	Suicide attempts in bipolar disorder	Psychiatric	42
	KDM2B	Parkinson`s disease	Psychiatric	42
	SENP8	Metabolite levels (MHPG)	Psychiatric	42
42	ATX3	Machado-Joseph disease, 109150	Neurological	49
	TERA	Inclusion body myopathy with early-onset Paget disease and frontotemporal dementia, 167320	Neurological	49
	UBE4B	Alzheimer`s disease (late onset)	Neurological	49
43	RAB3B	Alzheimer`s disease	Psychiatric	50
	RIMS2	Heroin addiction	Psychiatric	50
	RIMS2	Asperger disorder	Psychiatric	50
	RIMS2	Suicide attempts in bipolar disorder	Psychiatric	50
	RIMS2	Cocaine dependence	Psychiatric	50
	RIMS2	Parkinson`s disease (interaction with coffee consumption)	Psychiatric	50
	RIMS1	Schizophrenia	Psychiatric	50
	RIMS1	Coronary Artery Disease	Psychiatric	50
	RIMS1	Coronary heart disease	Psychiatric	50
	RIMS1	Alzheimer`s disease	Psychiatric	50
	RIMS1	Response to statin therapy	Psychiatric	50
	RIMS1	Cognitive impairment induced by topiramate	Psychiatric	50
	RIMS1	Personality dimensions	Psychiatric	50
44	RIMS1	Lung function (forced expiratory volume in 1 second to forced vital capacity ratio)	Respiratory	50
	RIMS1	Asthma (childhood onset)	Respiratory	50
	RIMS2	Lung function (forced expiratory volume in 1 second)	Respiratory	50
	RIMS2	Lung function (forced vital capacity)	Respiratory	50
	RAB3B	Smoking behavior	Respiratory	50
45	EIF3H	Alcohol dependence	Psychiatric	51
	EIF3F	Depression (quantitative trait)	Psychiatric	51

	EIF3D	Schizophrenia	Psychiatric	51
	EIF3F	Alzheimer`s disease	Psychiatric	51
	EIF3A	Cognitive test performance	Psychiatric	51
	EIF3E	Prostate cancer	Cancer	51
46	EIF3K	Lung adenocarcinoma	Cancer	51
	EIF3E	Chemotherapeutic response (cytarabine, 5`deoxyfluorouridine, carboplatin, cisplatin), in blood cell lines	Cancer	51
	EIF3L	Oral cancers (chewing tobacco related)	Cancer	51
	EI2BA	Leukoencephalopathy with vanishing white matter, 603896	Neurological	54
	EI2BD	Leukoencephaly with vanishing white matter, 603896	Neurological	54
	EI2BD	Ovarioleukodystrophy, 603896	Neurological	54
47	EI2BG	Leukoencephalopathy with vanishing white matter, 603896	Neurological	54
	EI2BB	Leukoencephalopathy with vanishing white matter, 603896	Neurological	54
	EI2BB	Ovarioleukodystrophy, 603896	Neurological	54
	EI2BE	Leukoencephalopathy with vanishing white matter, 603896	Neurological	54
	EI2BE	Ovarioleukodystrophy, 603896	Neurological	54
	LEPR	Glioma (high-grade)	Neurological	55
48	LEPR	Cognitive test performance	Neurological	55
	MAP1S	Cognitive impairment induced by topiramate	Neurological	55
	SOCS3	Dermatitis, atopic, 4, 605805	Neurological	55
	PGFRB	Myeloproliferative disorder with eosinophilia, 131440	Cancer	56
	PTEN	Cowden disease, 158350	Cancer	56
	PTEN	Endometrial carcinoma	Cancer	56
	PTEN	Lhermitte-Duclos syndrome	Cancer	56
49	PTEN	Meningioma, 607174	Cancer	56
	PTEN	Oligodendroglioma, 137800	Cancer	56
	PTEN	Prostate cancer, 176807	Cancer	56
	CFTR	Paclitaxel sensitivity in NCI60 cancer cell lines	Cancer	56
	PGFRB	Myelomonocytic leukemia, chronic	Cancer	56
	PTEN	Thyroid carcinoma, follicular, 188470	Cancer	56
	PTEN	Proteus syndrome, 176920	Cancer	56
	ACTN1	Brain structure	Neurological	61
	DRD3	Alzheimer`s disease	Neurological	61
	KIF1B	Bulimia nervosa	Neurological	61
	GTR1	Dystonia-18, 612126 (3)	Neurological	61
50	KIF1B	Charcot-Marie-Tooth disease, type 2A1, 118210 (3)	Neurological	61
	KIF1B	Multiple sclerosis	Neurological	61
	PDIA3	Parkinson`s disease	Neurological	61
	CLIC6	Odorant perception	Neurological	61
	DRD3	Essential tremor, susceptibility to, 190300 (3)	Neurological	61
51	ACTN1	Suicide attempts in bipolar disorder	Psychiatric	61
	GIPC1	Suicide attempts in bipolar disorder	Psychiatric	61
	ACTN1	Coronary heart disease	Psychiatric	61
	PDIA3	Coronary heart disease	Psychiatric	61
	KIF1B	Mean platelet volume	Psychiatric	61

	ACTN1	Brain structure	Psychiatric	61
	DRD3	Alzheimer`s disease	Psychiatric	61
	KIF1B	Bulimia nervosa	Psychiatric	61
	KIF1B	Triglycerides	Psychiatric	61
	PDIA3	Parkinson`s disease	Psychiatric	61
	DRD3	Schizophrenia, susceptibility to, 181500 (3)	Psychiatric	61
52	RFC2	Glycosylated haemoglobin levels	Endocrine	68
	RFC4	Type 2 diabetes	Endocrine	68
	RFC5	Metabolite levels	Endocrine	68
53	ABI1	Schizophrenia	Psychiatric	71
	ENAH	Alcohol and nictotine co-dependence	Psychiatric	71
	ABL1	Response to amphetamines	Psychiatric	71
	ABL1	Coronary heart disease	Psychiatric	71
	ENAH	Parkinson`s disease	Psychiatric	71
	ABL1	Personality dimensions	Psychiatric	71
	ENAH	Personality dimensions	Psychiatric	71
54	ABI1	Acute lung injury	Respiratory	71
	ENAH	Asthma	Respiratory	71
	ABL1	Lymphocyte counts	Respiratory	71
55	EPS8	Type 2 diabetes	Endocrine	71
	ABI1	Type 2 diabetes	Endocrine	71
	ENAH	Type 2 diabetes	Endocrine	71
56	AP1S2	Mental retardation, X-linked 59, 300630	Psychiatric	72
	EPN4	Schizophrenia, susceptibility to, 181510	Psychiatric	72
	AP1B1	Alzheimer`s disease	Psychiatric	72
	AP2B1	Mean platelet volume	Psychiatric	72
	AP1G1	Cognitive test performance	Psychiatric	72
	AP1G2	Information processing speed	Psychiatric	72
	AP1G2	Sudden cardiac arrest	Psychiatric	72
	AP1M2	Lipoprotein-associated phospholipase A2 activity and mass	Psychiatric	72
57	AKT1	Breast cancer, somatic, 114480	Cancer	74
	AKT1	Colorectal cancer, somatic, 114500	Cancer	74
	AKT1	Ovarian cancer, somatic, 604370	Cancer	74
	MDM2	Accelerated tumor formation, susceptibility to	Cancer	74
	P53	Breast cancer, 114480	Cancer	74
	P53	Colorectal cancer, 114500	Cancer	74
	P53	Histiocytoma	Cancer	74
	P53	Multiple malignancy syndrome	Cancer	74
	STK4	Melanoma	Cancer	74
	P53	Osteosarcoma	Cancer	74
	P53	Adrenal cortical carcinoma, 202300	Cancer	74
	P53	Thyroid carcinoma	Cancer	74
	P53	Pancreatic cancer, 260350	Cancer	74
	MYB	T-cell acute lymphoblastic leukemia	Cancer	74

	P53	Hepatocellular carcinoma, 114550	Cancer	74
	FOXO1	Rhabdomyosarcoma, alveolar, 268220	Cancer	74
	P53	Li-Fraumeni syndrome, 151623	Cancer	74
	P53	Nasopharyngeal carcinoma, 161550	Cancer	74
	STK4	Neuroblastoma	Cancer	74
58	PKN2	Monocyte counts	Cardiovascular	74
	P53	Adrenal cortical carcinoma, 202300	Cardiovascular	74
	FOXO1	Atrial fibrillation	Cardiovascular	74
	ARRB1	Thiazide-induced adverse metabolic effects in hypertensive patients	Cardiovascular	74
	MYB	Blood cell counts and traits, in red and white blood cells	Cardiovascular	74
59	MK10	Epileptic encephalopathy, Lennox-Gastaut type, 606369	Neurological	74
	PRKDC	Elbow pain	Neurological	74
	CHD3	Information processing speed	Neurological	74
	STK4	Neuroblastoma	Neurological	74
	PIAS4	Word reading	Neurological	74
	TOPRS	Retinitis pigmentosa-31, 609923	Neurological	74
	FOXO1	Rhabdomyosarcoma, alveolar, 268220	Neurological	74
	P53	Li-Fraumeni syndrome, 151623	Neurological	74
60	AMPH	Alzheimer`s disease (late onset)	Psychiatric	75
	AMPH	Coronary heart disease	Psychiatric	75
	AMPH	Alzheimer`s disease	Psychiatric	75
	AMPH	Bipolar disorder	Psychiatric	75
	AMPH	Job-related exhaustion	Psychiatric	75
	BIN1	Alcohol dependence	Psychiatric	75
	BIN1	Alzheimer`s disease	Psychiatric	75
	BIN1	Alzheimer`s disease (late onset)	Psychiatric	75
	BIN1	White matter integrity	Psychiatric	75
	BIN1	Progranulin levels	Psychiatric	75
	ARF1	Suicide attempts in bipolar disorder	Psychiatric	75
	HIP1	Alcoholism (heaviness of drinking)	Psychiatric	75
	HIP1	Alcoholism (12-month weekly alcohol consumption)	Psychiatric	75
	HIP1	Prion diseases	Psychiatric	75
	SNX4	Suicide attempts in bipolar disorder	Psychiatric	75
	SNX4	Major depressive disorder (broad)	Psychiatric	75
	SYNJ1	Suicide attempts in bipolar disorder	Psychiatric	75
	PDCD6	Alcohol dependence	Psychiatric	75
	GGA3	Warfarin maintenance dose	Psychiatric	75
	PACS1	Personality dimensions	Psychiatric	75
61	PLAK	Arrhythmogenic right ventricular dysplasia, familial, 12, 611528	Cardiovascular	76
	APC	Stroke	Cardiovascular	76
	DSC1	Blood pressure (response to angiotensin II receptor blocker)	Cardiovascular	76
	DSC1	Blood pressure, CVD RF and other traits	Cardiovascular	76
	LEF1	Blood pressure, CVD RF and other traits	Cardiovascular	76
	PTPRB	Calcium levels	Cardiovascular	76

	PSN1	Cardiomyopathy, dilated, 1U	Cardiovascular	76
	PSN2	Cardiomyopathy, dilated, 1V	Cardiovascular	76
	ICAM5	Soluble ICAM-1	Cardiovascular	76
	GSK3B	HDL cholesterol	Cardiovascular	76
	APC	Adenomatous polyposis coli, 175100	Cardiovascular	76
	PLAK	Naxos disease, 601214	Cardiovascular	76
62	APC	Adenoma, periampullary	Cancer	76
	APC	Colorectal cancer, somatic, 114500	Cancer	76
	CTNB1	Colorectal cancer	Cancer	76
	CTNB1	Ovarian carcinoma, endometrioid type	Cancer	76
	PTPRB	Response to gemcitabine or arabinosylcytosin in blood cell lines	Cancer	76
	ZEB1	Colorectal cancer	Cancer	76
	ICAM5	Breast cancer and prostate cancer	Cancer	76
	LEF1	Chronic lymphocytic leukemia	Cancer	76
	CTNB1	Pilomatricoma, 132600	Cancer	76
	LEF1	Sebaceous tumors, somatic	Cancer	76
	APC	Gastric cancer, somatic, 137215	Cancer	76
	APC	Gardner syndrome	Cancer	76
	APC	Hepatoblastoma	Cancer	76
	CTNB1	Hepatoblastoma	Cancer	76
	CTNB1	Hepatocellular carcinoma, 114550	Cancer	76
	APC	Desmoid disease, hereditary, 135290	Cancer	76
	APC	Brain tumor-polyposis syndrome 2	Cancer	76
	APC	Adenomatous polyposis coli, 175100	Cancer	76
63	CLOCK	Prostate cancer	Cancer	77
	EPAS1	Renal cell carcinoma	Cancer	77
	CLOCK	Chemotherapeutic response (cytarabine, 5`deoxyfluorouridine, carboplatin, cisplatin), in blood cell lines	Cancer	77
	VHL	Pheochromocytoma, 171300	Cancer	77
	VHL	Renal cell carcinoma, somatic, 144700	Cancer	77
	VHL	Hemangioblastoma, cerebellar, somatic	Cancer	77
	VHL	von Hippel-Lindau syndrome, 193300	Cancer	77
64	CCND1	Leukemia/lymphoma, chronic B-cell, 151400	Cancer	79
	CCND1	von Hippel-Lindau disease, modification of, 193300	Cancer	79
	CCND1	Colorectal cancer, susceptibility to	Cancer	79
	CDK4	Melanoma, 609048	Cancer	79
	CDN1B	Multiple endocrine neoplasia, type IV, 610755	Cancer	79
	RB	Bladder cancer, 109800	Cancer	79
	RB	Retinoblastoma	Cancer	79
	HMGA2	Lipoma	Cancer	79
	HMGA2	Lipomatosis, multiple, 151900	Cancer	79
	HMGA2	Salivary adenoma	Cancer	79
	HMGA2	Uterine leiomyoma	Cancer	79
	HMGA2	Stature QTL 9, 611547	Cancer	79
	CCND2	Colorectal cancer	Cancer	79

	CCND1	Multiple myeloma (IgH translocation)	Cancer	79
	CCND1	Multiple myeloma (hyperdiploidy)	Cancer	79
	CDK6	Stature QTL 11, 612223	Cancer	79
65	GRID2	Suicide attempts in bipolar disorder	Psychiatric	80
	GRID2	Major depressive disorder (broad)	Psychiatric	80
	BECN1	Depression (quantitative trait)	Psychiatric	80
	GOPC	Major depressive disorder	Psychiatric	80
	BCL2	Cardiac Troponin-T levels	Psychiatric	80
	GRID2	Pericardial fat	Psychiatric	80
	BCL2	Coronary heart disease	Psychiatric	80
	BID	Coronary heart disease	Psychiatric	80
	GRID2	Coronary restenosis	Psychiatric	80
	GRID2	Coronary Artery Disease	Psychiatric	80
	BAD	Mean platelet volume	Psychiatric	80
	GRID2	Alzheimer`s disease	Psychiatric	80
	GRID2	Amyotrophic Lateral Sclerosis	Psychiatric	80
	GRID2	Cholesterol	Psychiatric	80
	BIK	Cognitive impairment induced by topiramate	Psychiatric	80
	GRID2	Information processing speed	Psychiatric	80
	GRID2	Job-related exhaustion	Psychiatric	80
	GRID2	Relative hand skill in reading disability	Psychiatric	80
	GRID2	Intelligence	Psychiatric	80
66	CAC1C	Brugada syndrome 3, 611875	Cardiovascular	81
	CAC1C	Timothy syndrome, 601005	Cardiovascular	81
	KCNN2	Blood pressure, CVD RF and other traits	Cardiovascular	81
	KCNN2	Coronary arterial lesions in patients with Kawasaki disease	Cardiovascular	81
	KCNQ2	Blood pressure, CVD RF and other traits	Cardiovascular	81
67	TWST1	Saethre-Chotzen syndrome with eyelid anomalies, 101400	Hematological	82
	TWST1	Saethre-Chotzen syndrome, 101400	Hematological	82
	ID1	Red blood cell traits	Hematological	82
	TAL1	Red blood cell traits	Hematological	82
68	ERBB2	Ovarian cancer, somatic, 604370	Cancer	83
	KIT	Germ cell tumors, 273300	Cancer	83
	KIT	Mast cell leukemia	Cancer	83
	PK3CA	Breast cancer, somatic, 114480	Cancer	83
	PK3CA	Colorectal cancer, somatic, 114500	Cancer	83
	PK3CA	Keratosis, seborrheic, somatic, 182000	Cancer	83
	PK3CA	Ovarian cancer, somatic, 604370	Cancer	83
	NTF3	Response to gemcitabine or arabinosylcytosin in blood cell lines	Cancer	83
	FYN	Breast cancer	Cancer	83
	LAMA2	Methotrexate clearance (acute lymphoblastic leukemia)	Cancer	83
	LAMA2	Prostate cancer	Cancer	83
	LAMA5	Colorectal cancer	Cancer	83
	WIPF1	Methotrexate clearance (acute lymphoblastic leukemia)	Cancer	83

	SYNJ2	Colorectal cancer	Cancer	83
	FYN	Endometrial cancer	Cancer	83
	BCR	Response to platinum-based chemotherapy in small-cell lung cancer	Cancer	83
	GRB2	Response to taxane treatment (placlitaxel)	Cancer	83
	ITSN1	Response to taxane treatment (placlitaxel)	Cancer	83
	LAMA2	Chemotherapeutic response (cytarabine, 5`deoxyfluorouridine, carboplatin, cisplatin), in blood cell lines	Cancer	83
	SOS1	Noonan syndrome 4, 610733	Cancer	83
	PTN11	Leukemia, juvenile myelomonocytic, 607785	Cancer	83
	CSF1R	Myeloid malignancy, predisposition to	Cancer	83
	BCR	Leukemia, acute lymphocytic	Cancer	83
	BCR	Leukemia, chronic myeloid, 608232	Cancer	83
	KIT	Leukemia, acute myeloid, 601626	Cancer	83
	PTN11	Noonan syndrome 1, 163950	Cancer	83
	RET	Pheochromocytoma, 171300	Cancer	83
	ERBB2	Gastric cancer, somatic, 137215	Cancer	83
	KIT	Gastrointestinal stromal tumor, somatic, 606764	Cancer	83
	PK3CA	Gastric cancer, somatic, 137215	Cancer	83
	RET	Medullary thyroid carcinoma, 155240	Cancer	83
	RET	Multiple endocrine neoplasia IIA, 171400	Cancer	83
	RET	Multiple endocrine neoplasia IIB, 162300	Cancer	83
	SH21A	Lymphoproliferative syndrome, X-linked, 308240	Cancer	83
	WASP	Wiskott-Aldrich syndrome, 301000	Cancer	83
	PK3CA	Hepatocellular carcinoma, somatic, 114550	Cancer	83
	ERBB2	Glioblastoma, somatic, 137800	Cancer	83
	ERBB2	Adenocarcinoma of lung, somatic, 211980	Cancer	83
	PK3CA	Nonsmall cell lung cancer, somatic, 211980	Cancer	83
	PK3CA	Nevus, epidermal, 162900	Cancer	83
	BCR	Oral cancers (chewing tobacco related)	Cancer	83
	ITSN1	Oral cancers (chewing tobacco related)	Cancer	83
	CBLB	Lymphocyte counts	Cancer	83
	DAG1	Lymphocyte counts	Cancer	83
	FYB	Lymphocyte counts	Cancer	83
	IRS1	Lymphocyte counts	Cancer	83
	WASL	Lymphocyte counts	Cancer	83
	CAV3	Cardiomyopathy, familial hypertrophic, 192600	Cardiovascular	83
	CAV3	Long QT syndrome-9, 611818	Cardiovascular	83
	IRS1	Coronary artery disease, susceptibility to	Cardiovascular	83
	BTK	Atrial fibrillation	Cardiovascular	83
	CAV3	HDL particle features	Cardiovascular	83
69	CD3E	Response to statin treatment (atorvastatin), change in cholesterol levels	Cardiovascular	83
	FYN	Response to statin treatment (atorvastatin), change in cholesterol levels	Cardiovascular	83
	SYNJ2	Response to statin treatment (atorvastatin), change in cholesterol levels	Cardiovascular	83
	CD3E	Heart Failure	Cardiovascular	83
	CSF1R	Bicuspid aortic valve	Cardiovascular	83

	SYNJ2	Tunica Media	Cardiovascular	83
	SYNJ2	Heart Failure	Cardiovascular	83
	NTRK2	Left ventricular mass	Cardiovascular	83
	FYB	Blood pressure (response to angiotensin II receptor blocker)	Cardiovascular	83
	FYN	Blood pressure, CVD RF and other traits (body mass index (BMI), waist:hip ratio, renin activity in plasma, aldosterone concentration in plasma, BNP levels in plasma, alcohol consumption)	Cardiovascular	83
	FYN	Systolic blood pressure	Cardiovascular	83
	GAB1	Potassium levels	Cardiovascular	83
	LAMA2	Blood pressure, CVD RF and other traits (body mass index (BMI), waist:hip ratio, renin activity in plasma, aldosterone concentration in plasma, BNP levels in plasma, alcohol consumption)	Cardiovascular	83
	RET	Hypertension	Cardiovascular	83
	SOS1	Blood pressure, CVD RF and other traits (body mass index (BMI), waist:hip ratio, renin activity in plasma, aldosterone concentration in plasma, BNP levels in plasma, alcohol consumption)	Cardiovascular	83
	LAMA2	Chemotherapeutic response (cytarabine, 5`deoxyfluorouridine, carboplatin, cisplatin), in blood cell lines	Cardiovascular	83
	SOS1	Noonan syndrome 4, 610733	Cardiovascular	83
	PTN11	Noonan syndrome 1, 163950	Cardiovascular	83
	IRS1	Sudden cardiac arrest	Cardiovascular	83
	SF3B4	White blood cell count	Cardiovascular	83
	CBL	Platelet counts	Cardiovascular	83
	FYN	Coronary heart disease	Cardiovascular	83
	LAMA2	Coronary Artery Disease	Cardiovascular	83
	NTRK2	Coronary heart disease	Cardiovascular	83
	NTRK3	Coronary heart disease	Cardiovascular	83
	BLNK	Coronary heart disease	Cardiovascular	83
	GAB2	Menarche (age at onset)	Cardiovascular	83
	GAB1	Response to statin therapy	Cardiovascular	83
	GRAP2	Response to statin therapy	Cardiovascular	83
	PTN11	Leopard syndrome, 151100	Cardiovascular	83
	LAMA2	Muscular dystrophy, congenital merosin-deficient, 607855	Cardiovascular	83
	LAMA2	Muscular dystrophy, congenital, due to partial LAMA2 deficiency, 607855	Cardiovascular	83
	NTRK3	Gaucher disease severity	Cardiovascular	83
	CBLB	Lymphocyte counts	Cardiovascular	83
	DAG1	Lymphocyte counts	Cardiovascular	83
	FYB	Lymphocyte counts	Cardiovascular	83
	IRS1	Lymphocyte counts	Cardiovascular	83
	WASL	Lymphocyte counts	Cardiovascular	83
70	IRS1	Diabetes mellitus, noninsulin-dependent, 125853	Endocrine	83
	ERBB3	Polycystic ovary syndrome	Endocrine	83
	ERBB3	Type 1 diabetes	Endocrine	83
	ERBB3	Type 1 diabetes autoantibodies	Endocrine	83
	FYN	Insulin-related traits	Endocrine	83
	HCK	Metabolite levels	Endocrine	83
	LAMA2	Type 2 diabetes	Endocrine	83
	LAMA5	Type 2 diabetes	Endocrine	83

	LCP2	Type 2 diabetes	Endocrine	83
	NTF3	Type 2 diabetes	Endocrine	83
	NTRK2	Insulin-related traits	Endocrine	83
	NTRK3	Type 2 diabetes	Endocrine	83
	ITSN1	Insulin resistance	Endocrine	83
	ITSN1	Type 2 diabetes	Endocrine	83
	SOS1	Type 2 diabetes	Endocrine	83
	SOS1	Insulin resistance	Endocrine	83
	WIPF1	Insulin-related traits	Endocrine	83
	SKAP2	Type 2 diabetes	Endocrine	83
	SKAP2	Type 1 diabetes	Endocrine	83
	ITSN2	Type 2 diabetes	Endocrine	83
	SOS1	Noonan syndrome 4, 610733	Endocrine	83
	RET	Pheochromocytoma, 171300	Endocrine	83
	RET	Medullary thyroid carcinoma, 155240	Endocrine	83
	RET	Multiple endocrine neoplasia IIA, 171400	Endocrine	83
	RET	Multiple endocrine neoplasia IIB, 162300	Endocrine	83
	BTK	XLA and isolated growth hormone deficiency, 307200	Endocrine	83
	GAB2	Menarche (age at onset)	Endocrine	83
	CSF1R	Adverse response to chemotherapy (neutropenia/leucopenia) (doxorubicin)	Hematological	83
	LAMA2	Adverse response to chemotherapy (neutropenia/leucopenia) (doxorubicin)	Hematological	83
	NTRK3	Adverse response to chemotherapy (neutropenia/leucopenia) (epirubicin)	Hematological	83
	GAB2	Self-reported allergy	Hematological	83
	CSF1R	Atopy	Hematological	83
	PTN11	Leukemia, juvenile myelomonocytic, 607785	Hematological	83
	CSF1R	Myeloid malignancy, predisposition to	Hematological	83
	BCR	Leukemia, acute lymphocytic	Hematological	83
71	BCR	Leukemia, chronic myeloid, 608232	Hematological	83
	KIT	Leukemia, acute myeloid, 601626	Hematological	83
	SH21A	Lymphoproliferative syndrome, X-linked, 308240	Hematological	83
	WASP	Wiskott-Aldrich syndrome, 301000	Hematological	83
	NTRK2	Left ventricular mass	Hematological	83
	FYB	Blood pressure (response to angiotensin II receptor blocker)	Hematological	83
	FYN	Blood pressure, CVD RF and other traits (body mass index (BMI), waist:hip ratio, renin activity in plasma, aldosterone concentration in plasma, BNP levels in plasma, alcohol consumption)	Hematological	83
	FYN	Systolic blood pressure	Hematological	83
	GAB1	Potassium levels	Hematological	83
	LAMA2	Blood pressure, CVD RF and other traits (body mass index (BMI), waist:hip ratio, renin activity in plasma, aldosterone concentration in plasma, BNP levels in plasma, alcohol consumption)	Hematological	83
	RET	Hypertension	Hematological	83
	SOS1	Blood pressure, CVD RF and other traits (body mass index (BMI), waist:hip ratio, renin activity in plasma, aldosterone concentration in plasma, BNP levels in plasma, alcohol consumption)	Hematological	83
	BTK	Agammaglobulinemia, type 1, X-linked	Hematological	83
	EPOR	Erythrocytosis, familial, 133100	Hematological	83

	WASP	Neutropenia, severe congenital, X-linked, 300299	Hematological	83
	WASP	Thrombocytopenia, X-linked, 313900	Hematological	83
	WASP	Thrombocytopenia, X-linked, intermittent, 313900	Hematological	83
	BTK	XLA and isolated growth hormone deficiency, 307200	Hematological	83
	SF3B4	White blood cell count	Hematological	83
	CBL	Platelet counts	Hematological	83
72	DAG1	Crohn`s disease	Gastrointestinal	83
	RET	Hirschsprung`s disease	Gastrointestinal	83
	SKAP2	Crohn`s disease	Gastrointestinal	83
	CBLB	Celiac disease	Gastrointestinal	83
	LAMA2	Celiac disease	Gastrointestinal	83
	CBLB	Gallstones	Gastrointestinal	83
	NTRK2	Gallstones	Gastrointestinal	83
	ERBB2	Tooth agenesis (mandibular third molar)	Gastrointestinal	83
	LAMA2	Chemotherapeutic response (cytarabine, 5`deoxyfluorouridine, carboplatin, cisplatin), in blood cell lines	Gastrointestinal	83
	ERBB2	Gastric cancer, somatic, 137215	Gastrointestinal	83
	KIT	Gastrointestinal stromal tumor, somatic, 606764	Gastrointestinal	83
	PK3CA	Gastric cancer, somatic, 137215	Gastrointestinal	83
	RET	Medullary thyroid carcinoma, 155240	Gastrointestinal	83
	RET	Multiple endocrine neoplasia IIA, 171400	Gastrointestinal	83
	RET	Multiple endocrine neoplasia IIB, 162300	Gastrointestinal	83
	NTRK2	Chronic Hepatitis C infection	Gastrointestinal	83
	IRS1	Sudden cardiac arrest	Gastrointestinal	83
	SF3B4	White blood cell count	Gastrointestinal	83
	CBL	Platelet counts	Gastrointestinal	83
	FYN	Coronary heart disease	Gastrointestinal	83
	LAMA2	Coronary Artery Disease	Gastrointestinal	83
	NTRK2	Coronary heart disease	Gastrointestinal	83
	NTRK3	Coronary heart disease	Gastrointestinal	83
	BLNK	Coronary heart disease	Gastrointestinal	83
	GAB1	Response to statin therapy	Gastrointestinal	83
	GRAP2	Response to statin therapy	Gastrointestinal	83
	RET	Hirschsprung disease, 142623	Gastrointestinal	83
73	ITSN1	HIV-1 viral setpoint	Immunological	83
	GRAP2	HIV-1 progression	Immunological	83
	BCR	Immune response to smallpox vaccine (IL-6)	Immunological	83
	CD3E	Immunodeficiency due to defect in CD3-epsilon	Immunological	83
	BLNK	Hypoglobulinemia and absent B cells	Immunological	83
	GAB2	Self-reported allergy	Immunological	83
	CSF1R	Atopy	Immunological	83
	BCR	Leukemia, acute lymphocytic	Immunological	83
	BCR	Leukemia, chronic myeloid, 608232	Immunological	83
	KIT	Leukemia, acute myeloid, 601626	Immunological	83
	SH21A	Lymphoproliferative syndrome, X-linked, 308240	Immunological	83

	WASP	Wiskott-Aldrich syndrome, 301000	Immunological	83
	KIT	Mastocytosis with associated hematologic disorder	Immunological	83
	CBLB	Celiac disease	Immunological	83
	LAMA2	Celiac disease	Immunological	83
	NTRK2	Chronic Hepatitis C infection	Immunological	83
	CBL	Platelet counts	Immunological	83
	GRB2	Systemic lupus erythematosus and Systemic sclerosis	Immunological	83
	NTRK3	Odorant perception	Immunological	83
	NTF3	Myasthenia gravis	Immunological	83
	PLCG1	Taste perception	Immunological	83
	CBLB	Lymphocyte counts	Immunological	83
	DAG1	Lymphocyte counts	Immunological	83
	FYB	Lymphocyte counts	Immunological	83
	IRS1	Lymphocyte counts	Immunological	83
	WASL	Lymphocyte counts	Immunological	83
	ITK	Alopecia areata	Immunological	83
	ITSN2	Male-pattern baldness	Immunological	83
	BTK	Urinary metabolites	Renal	83
	CBL	Urinary metabolites	Renal	83
	ERBB2	Urinary metabolites	Renal	83
	FYN	Urinary metabolites	Renal	83
	LAMA5	Urinary metabolites	Renal	83
	NTRK2	Urinary metabolites	Renal	83
	NTRK3	Urinary metabolites	Renal	83
	SOS1	Urinary metabolites	Renal	83
	SKAP2	Urinary metabolites	Renal	83
	DOK5	Urinary metabolites	Renal	83
74	SOS1	Noonan syndrome 4, 610733	Renal	83
	CBLB	Gallstones	Renal	83
	NTRK2	Gallstones	Renal	83
	NTRK3	Amyotrophic Lateral Sclerosis	Renal	83
	SYNJ2	Amyotrophic Lateral Sclerosis	Renal	83
	FRS2	Amyotrophic Lateral Sclerosis	Renal	83
	RET	Renal agenesis, 191830	Renal	83
	PODO	Nephrotic syndrome, steroid-resistant, autosomal recessive, 600995	Renal	83
	NPHN	Nephrosis-1, congenital, Finnish type, 256300	Renal	83
	GRB2	Systemic lupus erythematosus and Systemic sclerosis	Renal	83
	PTN11	Noonan syndrome 1, 163950	Renal	83
	DAG1	Bipolar disorder, schizoaffective	Psychiatric	83
	NTRK3	Bipolar disorder	Psychiatric	83
	GRAP2	Bipolar disorder	Psychiatric	83
	GRAP2	Bipolar disorder and schizophrenia	Psychiatric	83
	BCR	Major depressive disorder	Psychiatric	83
75	LAMA2	Response to citalopram treatment	Psychiatric	83

NTF3	Major depressive disorder	Psychiatric	83
NTRK2	Suicide attempts in bipolar disorder	Psychiatric	83
NTRK3	Suicide attempts in bipolar disorder	Psychiatric	83
NTRK3	Brain derived neurotrophic factor levels, in serum	Psychiatric	83
PLCG1	Suicidal ideation	Psychiatric	83
WIPF1	Brain derived neurotrophic factor levels, in serum	Psychiatric	83
DOK5	Functional MRI	Psychiatric	83
NTRK3	Tourette syndrome	Psychiatric	83
NTRK3	Schizophrenia	Psychiatric	83
ITSN2	Response to antipsychotic treatment in schizophrenia (working memory)	Psychiatric	83
NTRK2	Nicotine smoking	Psychiatric	83
IRS1	Sudden cardiac arrest	Psychiatric	83
SF3B4	White blood cell count	Psychiatric	83
CBL	Platelet counts	Psychiatric	83
FYN	Coronary heart disease	Psychiatric	83
LAMA2	Coronary Artery Disease	Psychiatric	83
NTRK2	Coronary heart disease	Psychiatric	83
NTRK3	Coronary heart disease	Psychiatric	83
BLNK	Coronary heart disease	Psychiatric	83
CBLB	Alzheimer`s disease	Psychiatric	83
CSF1R	Alzheimer`s disease (late onset)	Psychiatric	83
NTRK2	Alzheimer`s disease (late onset)	Psychiatric	83
RET	Alzheimer`s disease (late onset)	Psychiatric	83
GAB2	Alzheimer`s disease	Psychiatric	83
GAB2	Alzheimer`s disease (late onset)	Psychiatric	83
ITSN2	Alzheimer`s disease	Psychiatric	83
DOK5	Alzheimer`s disease	Psychiatric	83
NTRK3	Amyotrophic Lateral Sclerosis	Psychiatric	83
SYNJ2	Amyotrophic Lateral Sclerosis	Psychiatric	83
FRS2	Amyotrophic Lateral Sclerosis	Psychiatric	83
GAB2	Menarche (age at onset)	Psychiatric	83
NTRK3	Autism spectrum disorder, attention deficit-hyperactivity disorder, bipolar disorder, major depressive disorder, and schizophrenia (combined)	Psychiatric	83
FYB	Alcohol and nictotine co-dependence	Psychiatric	83
ITK	Alcohol dependence	Psychiatric	83
LAMA2	Alcohol dependence	Psychiatric	83
LAMA2	Substance dependence	Psychiatric	83
NTF3	Alcoholism (heaviness of drinking)	Psychiatric	83
NTRK2	Alcohol consumption	Psychiatric	83
NTRK2	Alcohol dependence	Psychiatric	83
NTRK3	Alcohol dependence	Psychiatric	83
NTRK2	Heroin addiction	Psychiatric	83
SYNJ2	Alcohol dependence	Psychiatric	83
NTRK3	Creutzfeldt-Jakob disease (variant)	Psychiatric	83
GAB1	Response to statin therapy	Psychiatric	83

	GRAP2	Response to statin therapy	Psychiatric	83
	BTK	Parkinson`s disease	Psychiatric	83
	FYN	Parkinson`s disease	Psychiatric	83
	NTF3	Parkinson`s disease	Psychiatric	83
	HIP1R	Parkinson`s disease	Psychiatric	83
	ITSN2	Parkinson`s disease	Psychiatric	83
	BCR	Cognitive test performance	Psychiatric	83
	CBL	Personality dimensions	Psychiatric	83
	FYB	Cognitive impairment induced by topiramate	Psychiatric	83
	ITK	Personality dimensions	Psychiatric	83
	LAMA2	Cognitive decline	Psychiatric	83
	NTF3	Cognitive performance	Psychiatric	83
	NTF3	Cognitive impairment induced by topiramate	Psychiatric	83
	NTRK3	Cognitive test performance	Psychiatric	83
	RET	Cognitive test performance	Psychiatric	83
	WASL	Information processing speed	Psychiatric	83
	FRS2	Personality dimensions	Psychiatric	83
	DOK5	Cognitive decline	Psychiatric	83
	BCR	Narcolepsy	Psychiatric	83
	NTRK2	Volumetric brain MRI	Psychiatric	83
	NTRK3	Volumetric brain MRI	Psychiatric	83
	AATF	Neuroticism	Psychiatric	83
	DOK5	Intelligence (childhood)	Psychiatric	83
	ERBB2	Glioblastoma, somatic, 137800	Neurological	83
	NTRK3	Creutzfeldt-Jakob disease (variant)	Neurological	83
	NTRK3	Schizophrenia	Neurological	83
	ITSN2	Response to antipsychotic treatment in schizophrenia (working memory)	Neurological	83
	CBLB	Alzheimer`s disease	Neurological	83
	CSF1R	Alzheimer`s disease (late onset)	Neurological	83
	NTRK2	Alzheimer`s disease (late onset)	Neurological	83
	RET	Alzheimer`s disease (late onset)	Neurological	83
	GAB2	Alzheimer`s disease	Neurological	83
	GAB2	Alzheimer`s disease (late onset)	Neurological	83
	ITSN2	Alzheimer`s disease	Neurological	83
	DOK5	Alzheimer`s disease	Neurological	83
	NTRK3	Amyotrophic Lateral Sclerosis	Neurological	83
	SYNJ2	Amyotrophic Lateral Sclerosis	Neurological	83
	FRS2	Amyotrophic Lateral Sclerosis	Neurological	83
	GAB2	Menarche (age at onset)	Neurological	83
	NTRK3	Autism spectrum disorder, attention deficit-hyperactivity disorder, bipolar disorder, major depressive disorder, and schizophrenia (combined)	Neurological	83
	NTRK3	Statin-induced myopathy	Neurological	83
	GAB1	Response to statin therapy	Neurological	83
	GRAP2	Response to statin therapy	Neurological	83
76	CAV3	Rippling muscle disease, 606072	Neurological	83

TAU	Tauopathy and respiratory failure	Neurological	83
1433E	Miller-Dieker lissencephaly, 247200	Neurological	83
PTN11	Leopard syndrome, 151100	Neurological	83
TAU	Supranuclear palsy, progressive atypical, 260540	Neurological	83
TAU	Supranuclear palsy, progressive, 601104	Neurological	83
RET	Hirschsprung disease, 142623	Neurological	83
TAU	Dementia, frontotemporal, with or without parkinsonism, 600274	Neurological	83
TAU	Pick disease, 172700	Neurological	83
TAU	Parkinson disease, 168600	Neurological	83
DYN2	Charcot-Marie-Tooth disease, dominant intermediate B, 606482	Neurological	83
CAV3	Muscular dystrophy, limb-girdle, type IC, 607801	Neurological	83
CAV3	Myopathy, distal, with decreased caveolin 3	Neurological	83
LAMA2	Muscular dystrophy, congenital merosin-deficient, 607855	Neurological	83
LAMA2	Muscular dystrophy, congenital, due to partial LAMA2 deficiency, 607855	Neurological	83
RET	Central hypoventilation syndrome, congenital, 209880	Neurological	83
CBLB	Multiple sclerosis	Neurological	83
ERBB3	Multiple sclerosis	Neurological	83
LAMA2	Partial epilepsies	Neurological	83
SKAP2	Multiple sclerosis	Neurological	83
WASL	Migraine	Neurological	83
BTK	Parkinson`s disease	Neurological	83
FYN	Parkinson`s disease	Neurological	83
NTF3	Parkinson`s disease	Neurological	83
HIP1R	Parkinson`s disease	Neurological	83
ITSN2	Parkinson`s disease	Neurological	83
NTRK3	Odorant perception	Neurological	83
BCR	Cognitive test performance	Neurological	83
CBL	Personality dimensions	Neurological	83
FYB	Cognitive impairment induced by topiramate	Neurological	83
ITK	Personality dimensions	Neurological	83
LAMA2	Cognitive decline	Neurological	83
NTF3	Cognitive performance	Neurological	83
NTF3	Cognitive impairment induced by topiramate	Neurological	83
NTRK3	Cognitive test performance	Neurological	83
RET	Cognitive test performance	Neurological	83
WASL	Information processing speed	Neurological	83
FRS2	Personality dimensions	Neurological	83
DOK5	Cognitive decline	Neurological	83
NTRK3	Gaucher disease severity	Neurological	83
NTF3	Myasthenia gravis	Neurological	83
PLCG1	Taste perception	Neurological	83
BCR	Narcolepsy	Neurological	83
NTRK2	Volumetric brain MRI	Neurological	83
NTRK3	Volumetric brain MRI	Neurological	83

	AATF	Neuroticism	Neurological	83
	DOK5	Intelligence (childhood)	Neurological	83
	DYN2	Myopathy, centronuclear, 160150	Neurological	83
	BCR	Lung function (forced expiratory volume in 1 second to forced vital capacity ratio)	Respiratory	83
	BCR	Acute lung injury	Respiratory	83
	CD3E	Lung function (forced expiratory volume in 1 second to forced vital capacity ratio)	Respiratory	83
	CD3E	Lung function (forced expiratory flow between 25% and 75% of forced vital capacity)	Respiratory	83
	ERBB2	Asthma	Respiratory	83
	ERBB3	Asthma	Respiratory	83
	GAB1	Asthma	Respiratory	83
	NTRK2	Lung function (forced vital capacity)	Respiratory	83
	NTRK3	Lung function (forced expiratory volume in 1 second)	Respiratory	83
	NTRK3	Lung function (forced vital capacity)	Respiratory	83
	ITSN1	Lung function (forced vital capacity)	Respiratory	83
	SKAP2	Lung function (forced vital capacity)	Respiratory	83
	CBLB	Lymphocyte counts	Respiratory	83
	DAG1	Lymphocyte counts	Respiratory	83
	FYB	Lymphocyte counts	Respiratory	83
	IRS1	Lymphocyte counts	Respiratory	83
	WASL	Lymphocyte counts	Respiratory	83
	RET	Smoking behavior	Respiratory	83
	ERBB3	Lethal congenital contractural syndrome 2, 607598	Respiratory	83
	CSF1R	Response to TNF antagonist treatment	Respiratory	83
	ITK	Rheumatoid arthritis	Respiratory	83
	NTRK2	Rheumatoid arthritis	Respiratory	83
	BCR	Response to platinum-based chemotherapy in small-cell lung cancer	Respiratory	83
	GRB2	Response to taxane treatment (placlitaxel)	Respiratory	83
	ITSN1	Response to taxane treatment (placlitaxel)	Respiratory	83
	GRB2	Systemic lupus erythematosus and Systemic sclerosis	Respiratory	83
	RET	Central hypoventilation syndrome, congenital, 209880	Respiratory	83
77	NTRK3	Gaucher disease severity	Respiratory	83
	ARHG6	Mental retardation, X-linked nonspecific, type 46, 300436	Neurological	85
	PAK1	Parkinson`s disease	Neurological	85
	PAK3	Mental retardation, X-linked 30, 300558	Neurological	85
78	PARD3	Parkinson`s disease (motor and cognition)	Neurological	85
	PAK1	Pure-tone audiometry	Neurological	85
	WASF1	Cognitive decline	Neurological	85
	PARD3	Cognitive test performance	Neurological	85
	DAB1	Methotrexate clearance (acute lymphoblastic leukemia)	Cancer	88
	DAB1	Prostate cancer	Cancer	88
	DAB1	Mammographic density	Cancer	88
	SNX17	Breast size	Cancer	88
	APBB3	Prostate cancer	Cancer	88

79	LRP1	Endometrial cancer	Cancer	88
	APBA2	Response to platinum-based chemotherapy in small cell and non-small cell lung cancers	Cancer	88
	DAB1	Non-small cell lung cancer	Cancer	88
	DAB1	Response to taxane treatment (placlitaxel)	Cancer	88
	VLDLR	Lung adenocarcinoma	Cancer	88
	LRP8	Response to taxane treatment (placlitaxel)	Cancer	88
	LDLR	Multiple cancers (lung cancer, gastric cancer, and squamous cell carcinoma)	Cancer	88
	DAB1	Chemotherapeutic response (cytarabine, 5`deoxyfluorouridine, carboplatin, cisplatin), in blood cell lines	Cancer	88
	DAB1	Acute lymphoblastic leukemia (childhood)	Cancer	88
	APBA2	Response to mTOR inhibitor (rapamycin)	Cancer	88
	DAB1	Response to mTOR inhibitor (rapamycin)	Cancer	88
80	LRP8	Myocardial infarction, susceptibility to, 608446	Cardiovascular	88
	DAB1	Response to statin treatment (atorvastatin), change in cholesterol levels	Cardiovascular	88
	DAB1	QT interval	Cardiovascular	88
	DAB1	Antineutrophil cytoplasmic antibody-associated vasculitis	Cardiovascular	88
	LDLR	Carotid intima media thickness	Cardiovascular	88
	LDLR	Cardiovascular disease risk factors	Cardiovascular	88
	LDLR	Abdominal aortic aneurysm	Cardiovascular	88
	LDLR	LDL lipoproteins	Cardiovascular	88
	LDLR	Cardiovascular disease	Cardiovascular	88
	LRP1	Abdominal aortic aneurysm	Cardiovascular	88
	DAB1	Blood pressure, CVD RF and other traits	Cardiovascular	88
	DAB1	Blood Pressure	Cardiovascular	88
	DAB1	Body Fat Distribution	Cardiovascular	88
	DAB1	Eosinophil counts	Cardiovascular	88
	DAB1	Chemotherapeutic response (cytarabine, 5`deoxyfluorouridine, carboplatin, cisplatin), in blood cell lines	Cardiovascular	88
	DAB1	Glycosylated haemoglobin levels	Cardiovascular	88
	LDLR	Multiple traits (coronary heart disease, T2D, LDL cholesterol, HDL cholesterol)	Cardiovascular	88
	LDLR	Lipoprotein-associated phospholipase A2 activity and mass	Cardiovascular	88
	DAB1	Coronary Artery Disease	Cardiovascular	88
	LDLR	Coronary heart disease	Cardiovascular	88
	LDLR	Coronary Artery Disease	Cardiovascular	88
	LDLR	Lipid levels	Cardiovascular	88
	LDLR	Lipid traits	Cardiovascular	88
	LDLR	Triglycerides	Cardiovascular	88
	SNX17	Triglycerides	Cardiovascular	88
	LDLR	Cholesterol	Cardiovascular	88
	LDLR	Cholesterol, total	Cardiovascular	88
	VLDLR	Cholesterol, total	Cardiovascular	88
	LDLR	Response to statin therapy	Cardiovascular	88
	SNX17	Response to statin therapy	Cardiovascular	88
	JIP1	Diabetes mellitus, noninsulin-dependent, 125853	Endocrine	88
	APBA2	Type 2 diabetes	Endocrine	88

81	DAB1	Type 2 diabetes and other traits	Endocrine	88
	DAB1	Type 2 diabetes	Endocrine	88
	LDLR	Metabolite levels	Endocrine	88
	LDLR	Type 2 diabetes	Endocrine	88
	LRP8	Type 2 diabetes	Endocrine	88
	SNX17	Metabolite levels	Endocrine	88
	DAB1	Glycosylated haemoglobin levels	Endocrine	88
	LDLR	Hypercholesterolemia, familial, 143890	Endocrine	88
82	DAB1	Bipolar disorder and schizophrenia	Psychiatric	88
	LRP8	Bipolar disorder and schizophrenia	Psychiatric	88
	DAB1	Suicide attempts in bipolar disorder	Psychiatric	88
	DAB1	Major depressive disorder	Psychiatric	88
	LDLR	Multiple traits (coronary heart disease, T2D, LDL cholesterol, HDL cholesterol)	Psychiatric	88
	DAB1	Schizophrenia	Psychiatric	88
	LRP8	Schizophrenia	Psychiatric	88
	LDLR	Lipoprotein-associated phospholipase A2 activity and mass	Psychiatric	88
	DAB1	Coronary Artery Disease	Psychiatric	88
	LDLR	Coronary heart disease	Psychiatric	88
	LDLR	Coronary Artery Disease	Psychiatric	88
	DAB1	Alzheimer`s disease	Psychiatric	88
	DAB1	Alzheimer`s disease (late onset)	Psychiatric	88
	DAB1	Bulimia nervosa	Psychiatric	88
	LRP1	Brain structure	Psychiatric	88
	DAB1	Amyotrophic Lateral Sclerosis	Psychiatric	88
	LDLR	Triglycerides	Psychiatric	88
	SNX17	Triglycerides	Psychiatric	88
	LDLR	Cholesterol	Psychiatric	88
	LDLR	Cholesterol, total	Psychiatric	88
	VLDLR	Cholesterol, total	Psychiatric	88
	LDLR	Response to statin therapy	Psychiatric	88
	SNX17	Response to statin therapy	Psychiatric	88
	DAB1	Parkinson`s disease	Psychiatric	88
	DAB1	Cognitive performance	Psychiatric	88
	DAB1	Cognitive impairment induced by topiramate	Psychiatric	88
	APBA2	Temperament	Psychiatric	88
	DAB1	Neuroticism	Psychiatric	88
	DAB1	Intelligence	Psychiatric	88
	A4	Alzheimer disease-1, APP-related	Psychiatric	88
	APBA2	Alcohol consumption	Psychiatric	88
	DAB1	Alcohol dependence	Psychiatric	88
	LRP8	Alcohol dependence	Psychiatric	88
	A4	Amyloidosis, cerebroarterial, Iowa type, 605714	Neurological	88
	VLDLR	Cerebellar hypoplasia and mental retardation with or without quadrupedal locomotion, 224050	Neurological	88
	DAB1	Epilepsy (remission after treatment)	Neurological	88

83	LRP1	Migraine	Neurological	88
	LRP1	Migraine - clinic-based	Neurological	88
	LRP1	Migraine without aura	Neurological	88
	LRP1	Migraine with aura	Neurological	88
	VLDLR	Epilepsy	Neurological	88
	DAB1	Parkinson`s disease	Neurological	88
	DAB1	Cognitive performance	Neurological	88
	DAB1	Cognitive impairment induced by topiramate	Neurological	88
	APBA2	Response to mTOR inhibitor (rapamycin)	Neurological	88
	DAB1	Response to mTOR inhibitor (rapamycin)	Neurological	88
	DAB1	Taste perception	Neurological	88
	APBA2	Temperament	Neurological	88
	DAB1	Neuroticism	Neurological	88
	DAB1	Intelligence	Neurological	88
	A4	Alzheimer disease-1, APP-related	Neurological	88
	A4	Amyloidosis, cerebroarterial, Dutch type	Neurological	88
	LRP8	Body mass index (interaction)	Neurological	88
	DAB1	Hearing function	Neurological	88
	DAB1	Schizophrenia	Neurological	88
	LRP8	Schizophrenia	Neurological	88
	DAB1	Alzheimer`s disease	Neurological	88
	DAB1	Alzheimer`s disease (late onset)	Neurological	88
	DAB1	Bulimia nervosa	Neurological	88
	LRP1	Brain structure	Neurological	88
	DAB1	Amyotrophic Lateral Sclerosis	Neurological	88
	LDLR	Response to statin therapy	Neurological	88
	SNX17	Response to statin therapy	Neurological	88
84	SDC2	Coronary heart disease	Gastrointestinal	89
	LIN7A	Celiac disease	Gastrointestinal	89
	LIN7C	Pancreatic cancer	Gastrointestinal	89
85	APBA1	Schizophrenia(age at onset)	Neurological/Mental	89
	APBA1	Alzheimer`s disease (late onset)	Neurological/Mental	89
	SDC2	Cognitive performance	Neurological/Mental	89
	CSKP	Mental retardation and microcephaly with pontine and cerebellar hypoplasia, 300749 (3)	Neurological/Mental	89
	ERBB4	Chemotherapeutic response (cytarabine, 5`deoxyfluorouridine, carboplatin, cisplatin), in blood cell lines	Cardiovascular	90
	ACTN2	Cardiomyopathy, dilated, 1AA, 612158	Cardiovascular	90
	ADRB1	Resting heart rate, 607276	Cardiovascular	90
	ADRB1	congestive heart failure, susceptibility to	Cardiovascular	90
	IRK2	Long QT syndrome-7, 170390	Cardiovascular	90
	IRK2	Short QT syndrome-3, 609622	Cardiovascular	90
	LDB3	Cardiomyopathy, dilated, with left ventricular noncompaction	Cardiovascular	90
	DLG3	Fibrinogen	Cardiovascular	90
	ERBB4	Fibrinogen	Cardiovascular	90

86	ERBB4	Response to statin treatment (atorvastatin), change in cholesterol levels	Cardiovascular	90
	ADRB1	Birth weight	Cardiovascular	90
	DLG2	stroke (ischemic)	Cardiovascular	90
	DLG2	Myocardial Infarction	Cardiovascular	90
	DLG2	Stroke	Cardiovascular	90
	DLG2	Heart Failure	Cardiovascular	90
	ERBB4	Left ventricular hypertrophy	Cardiovascular	90
	NLGN1	Cardiovascular disease	Cardiovascular	90
	NLGN1	Myocardial Infarction	Cardiovascular	90
	DLG1	Blood pressure, CVD RF and other traits	Cardiovascular	90
	DLG2	Blood pressure, CVD RF and other traits	Cardiovascular	90
	GRIK2	Blood Pressure	Cardiovascular	90
	GRIK2	Blood pressure, CVD RF and other traits	Cardiovascular	90
	NLGN1	Blood pressure, CVD RF and other traits	Cardiovascular	90
	NLGN1	Blood pressure (response to angiotensin II receptor blocker)	Cardiovascular	90
	NLGN1	Thiazide-induced adverse metabolic effects in hypertensive patients	Cardiovascular	90
	LDB3	Cardiomyopathy, dilated	Cardiovascular	90
	DLG2	Glucose levels	Cardiovascular	90
	ERBB4	Tuberculosis	Cardiovascular	90
	GRIK2	Ankle-brachial index	Cardiovascular	90
	ERBB4	Pericardial fat	Cardiovascular	90
	ERBB4	Serum dimethylarginine levels (symmetric)	Cardiovascular	90
	DLG2	Phospholipid levels (plasma)	Cardiovascular	90
	DLG2	Coronary heart disease	Cardiovascular	90
	DLG2	HDL cholesterol	Cardiovascular	90
	ERBB4	HDL cholesterol	Cardiovascular	90
	IL16	Coronary heart disease	Cardiovascular	90
	DLG4	Adiponectin levels	Cardiovascular	90
	GRIK2	Triglycerides	Cardiovascular	90
	GRIK2	Polyunsaturated fatty acid levels, in plasma	Cardiovascular	90
	GRIK2	Prion diseases	Cardiovascular	90
	IL16	Lymphocyte counts	Cardiovascular	90
87	ACTN2	Type 2 diabetes	Endocrine	90
	CDK5	Insulin resistance	Endocrine	90
	DLG2	Type 2 diabetes	Endocrine	90
	DLG2	Type 2 diabetes and 6 quantitative traits	Endocrine	90
	DLG4	Metabolite levels	Endocrine	90
	ERBB4	Type 2 diabetes	Endocrine	90
	ERBB4	Type 1 diabetes nephropathy	Endocrine	90
	GRIK2	Type 2 diabetes	Endocrine	90
	GRIK2	Insulin resistance	Endocrine	90
	GRIK2	Type 1 diabetes	Endocrine	90
	NLGN1	Type 2 diabetes	Endocrine	90
	NLGN1	Metabolite levels	Endocrine	90

	DLG2	Glucose levels	Endocrine	90
	DLG2	Urinary albumin excretion	Endocrine	90
	MAP1A	Tumor biomarkers	Endocrine	90
	ADRB1	Birth weight	Endocrine	90
	DLG2	Kidney function and endocine traits	Endocrine	90
	DLG4	Adiponectin levels	Endocrine	90
	GRIK2	Diabetic nephropathy	Endocrine	90
	GRIK2	Polyunsaturated fatty acid levels, in plasma	Endocrine	90
88	DVL3	Major depressive disorder	Psychiatric	91
	SMAD3	Coronary Artery Disease	Psychiatric	91
	SMAD3	Coronary heart disease	Psychiatric	91
	SMAD3	Alzheimer`s disease (late onset)	Psychiatric	91
	SMAD7	Tourette syndrome	Psychiatric	91
89	SMAD1	Duodenal ulcer	Gastrointestinal	91
	SMAD3	Coronary Artery Disease	Gastrointestinal	91
	SMAD3	Crohn`s disease	Gastrointestinal	91
	SMAD3	Inflammatory bowel disease	Gastrointestinal	91
	SMAD3	Coronary heart disease	Gastrointestinal	91
	SMAD4	Juvenile polyposis/hereditary hemorrhagic telangiectasia syndrome, 175050	Gastrointestinal	91
	SMAD4	Pancreatic cancer	Gastrointestinal	91
	SMAD4	Polyposis, juvenile intestinal, 174900	Gastrointestinal	91
	SMAD6	Chemotherapeutic response (cytarabine, 5`deoxyfluorouridine, carboplatin, cisplatin), in blood cell lines	Gastrointestinal	91
90	SMAD7	Colorectal cancer, susceptiblity to, 3, 612229	Cancer	91
	SMAD3	Cisplatin and carboplatin cytotoxicity, in blood cell lines	Cancer	91
	SMAD6	Prostate cancer	Cancer	91
	SMAD7	Colorectal cancer	Cancer	91
	SMAD7	Cisplatin and carboplatin cytotoxicity, in blood cell lines	Cancer	91
	SMAD3	Response to taxane treatment (placlitaxel)	Cancer	91
	SMAD7	Nasopharyngeal carcinoma	Cancer	91
	SMAD6	Chemotherapeutic response (cytarabine, 5`deoxyfluorouridine, carboplatin, cisplatin), in blood cell lines	Cancer	91
	SMAD4	Juvenile polyposis/hereditary hemorrhagic telangiectasia syndrome, 175050	Cancer	91
	SMAD4	Polyposis, juvenile intestinal, 174900	Cancer	91
	SMAD4	Pancreatic cancer	Cancer	91
91	KLF6	Prostate cancer, somatic, 176807	Cancer	92
	E2F1	Melanoma	Cancer	92
	NFYC	Cervical cancer	Cancer	92
	TFDP1	Esophageal cancer (squamous cell)	Cancer	92
	TFDP2	Lung cancer	Cancer	92
	KLF6	Gastric cancer, somatic, 137215	Cancer	92
	CREB1	Histiocytoma, angiomatoid fibrous, somatic, 612160	Cancer	94
	CBP	Rubenstein-Taybi syndrome, 180849	Cancer	94
	EP300	Colorectal cancer, 114500	Cancer	94
	EP300	Rubinstein-Taybi syndrome, 180849	Cancer	94

92	GATA1	Leukemia, megakaryoblastic, of Down syndrome, 190685	Cancer	94
	GATA1	Leukemia, megakaryoblastic, with or without Down syndrome, 190685	Cancer	94
	VHL	Hemangioblastoma, cerebellar, somatic	Cancer	94
	VHL	Pheochromocytoma, 171300	Cancer	94
	VHL	Renal cell carcinoma, somatic, 144700	Cancer	94
	VHL	von Hippel-Lindau syndrome, 193300	Cancer	94
	HDAC4	Response to gemcitabine or arabinosylcytosin in blood cell lines	Cancer	94
	NCOA3	Response to treatment for acute lymphoblastic leukemia	Cancer	94
	HDAC4	Methotrexate clearance (acute lymphoblastic leukemia)	Cancer	94
	HDAC4	Melanoma	Cancer	94
	HDAC4	Cisplatin and carboplatin cytotoxicity, in blood cell lines	Cancer	94
93	EP300	Hemoglobin	Hematological	94
	HDAC4	Hemoglobin concentration	Hematological	94
	EP300	Self-reported allergy	Hematological	94
	HIF1A	Blood Pressure	Hematological	94
	HDAC4	Blood pressure, CVD RF and other traits	Hematological	94
	GATA1	Dyserythropoietic anemia with thrombocytopenia, 300367	Hematological	94
	GATA1	Macrothrombocytopenia, 300367	Hematological	94
	VHL	Polycythemia, benign familial, 263400	Hematological	94
	HDAC4	IgE levels	Hematological	94
94	ANDR	Androgen insensitivity, 300068	Endocrine	96
	ANDR	Hypospadias, perineal, 300633	Endocrine	96
	ESR1	Estrogen resistance	Endocrine	96
	ESR1	Type 2 diabetes	Endocrine	96
	NCOA2	Type 2 diabetes	Endocrine	96
	NCOA2	Metabolite levels (MHPG)	Endocrine	96
95	ANDR	Prostate cancer	Cancer	96
	ANDR	Prostate cancer, susceptibility to, 176807	Cancer	96
	ESR1	Breast cancer	Cancer	96
	ESR1	Response to taxane treatment (placlitaxel)	Cancer	96
	NCOA2	Non-small cell lung cancer	Cancer	96
	ANDR	Breast cancer, male, with Reifenstein syndrome	Cancer	96
96	CTBP2	Personality dimensions	Psychiatric	97
	CTBP2	Alzheimer`s disease	Psychiatric	97
	CTBP2	Alcohol and nictotine co-dependence	Psychiatric	97
	CTBP2	Alcohol dependence	Psychiatric	97
	CTBP2	Depression (quantitative trait)	Psychiatric	97
	CTBP2	White matter integrity	Psychiatric	97
	EZH1	Personality dimensions	Psychiatric	97
	EZH2	Alzheimer`s disease	Psychiatric	97
	RBBP4	Coronary heart disease	Psychiatric	97
	EED	Cholesterol	Psychiatric	97
	GRB14	Major depressive disorder (broad)	Psychiatric	98
	FGF1	Nicotine smoking	Psychiatric	98

97	GRB14	Nicotine smoking	Psychiatric	98
	FGFR1	Coronary heart disease	Psychiatric	98
	FGFR2	ldl cholesterol	Psychiatric	98
	FGF1	Amyotrophic Lateral Sclerosis	Psychiatric	98
	FRS2	Amyotrophic Lateral Sclerosis	Psychiatric	98
	FRS2	Personality dimensions	Psychiatric	98
98	FGFR2	Craniosynostosis, nonspecific	Bone	98
	FGFR2	Jackson-Weiss syndrome, 123150	Bone	98
	FGFR1	Jackson-Weiss syndrome, 123150	Bone	98
	FGFR2	Gout	Bone	98
	FGF2	Body mass index	Bone	98
	FGF1	Rheumatoid arthritis	Bone	98
	FGFR2	Beare-Stevenson cutis gyrata syndrome, 123790	Bone	98
	FGFR2	Craniofacial-skeletal-dermatologic dysplasia	Bone	98
	FGFR2	Saethre-Chotzen syndrome, 101400	Bone	98
	FGFR2	Apert syndrome, 101200	Bone	98
	FGFR2	Crouzon syndrome, 123500	Bone	98
	FGFR1	Pfeiffer syndrome, 101600	Bone	98
	FGFR2	Pfeiffer syndrome, 101600	Bone	98
99	IGF1R	Response to taxane treatment (placlitaxel)	Cancer	101
	ARHGC	Leukemia, acute myeloid, 601626	Cancer	101
	GRB10	Acute lymphoblastic leukemia (childhood)	Cancer	101
	IGF1R	Acute lymphoblastic leukemia (childhood)	Cancer	101
	GRIA3	Bipolar disorder (mania)	Psychiatric	102
	GRM3	Bipolar disorder	Psychiatric	102
	GRM7	Bipolar disorder, schizoaffective	Psychiatric	102
	SDC2	Bipolar disorder and schizophrenia	Psychiatric	102
	GRIA1	Depression (quantitative trait)	Psychiatric	102
	GRIA3	Suicidal ideation with citalopram treatment	Psychiatric	102
	GRIK1	Suicide attempts in bipolar disorder	Psychiatric	102
	GRM7	Panic disorder	Psychiatric	102
	GRM7	Major depressive disorder (broad)	Psychiatric	102
	GRM7	Major depressive disorder	Psychiatric	102
	GRM7	Suicide attempts in bipolar disorder	Psychiatric	102
	SDC2	Response to lithium treatment in bipolar disorder	Psychiatric	102
	GRIP1	Suicidal ideation	Psychiatric	102
	GRM7	Tourette syndrome	Psychiatric	102
	GRIK1	Schizophrenia (cytomegalovirus infection interaction)	Psychiatric	102
	GRM7	Schizophrenia	Psychiatric	102
	GRIA3	Nicotine dependence	Psychiatric	102
	GRM7	Nicotine smoking	Psychiatric	102
	GRIA1	Sudden cardiac arrest	Psychiatric	102
	GRIP1	Platelet counts	Psychiatric	102
	GRIA1	Coronary Artery Disease	Psychiatric	102

100	GRM7	Coronary heart disease	Psychiatric	102
	SDC2	Coronary heart disease	Psychiatric	102
	GRIA1	Alzheimer`s disease	Psychiatric	102
	GRIA4	Alzheimer`s disease (late onset)	Psychiatric	102
	GRM7	Alzheimer`s disease	Psychiatric	102
	GRIP1	Alzheimer`s disease	Psychiatric	102
	GRIA3	Amyotrophic Lateral Sclerosis	Psychiatric	102
	GRM3	Amyotrophic Lateral Sclerosis	Psychiatric	102
	GRM7	Amyotrophic Lateral Sclerosis	Psychiatric	102
	GRIP1	Amyotrophic Lateral Sclerosis	Psychiatric	102
	GRIK1	Autism spectrum disorder, attention deficit-hyperactivity disorder, bipolar disorder, major depressive disorder, and schizophrenia (combined)	Psychiatric	102
	GRIA3	Mental retardation, X-linked 94, 300699	Psychiatric	102
	GRIA3	Parkinson`s disease	Psychiatric	102
	GRIA4	Parkinson`s disease	Psychiatric	102
	GRM7	Parkinson`s disease	Psychiatric	102
	NSF	Parkinson`s disease	Psychiatric	102
	GRIA1	Cognitive impairment induced by topiramate	Psychiatric	102
	GRIA1	Cognitive decline	Psychiatric	102
	GRIA2	Cognitive impairment induced by topiramate	Psychiatric	102
	GRIA4	Cognitive impairment induced by topiramate	Psychiatric	102
	GRM7	Personality dimensions	Psychiatric	102
	GRM7	Cognitive impairment induced by topiramate	Psychiatric	102
	GRM7	Cognitive performance	Psychiatric	102
	SDC2	Cognitive performance	Psychiatric	102
	GRIP1	Cognitive impairment induced by topiramate	Psychiatric	102
	GRM3	Intelligence	Psychiatric	102
	SC6A3	Major affective disorder, 125480	Psychiatric	102
	SC6A3	Nicotine dependence, protection against, 188890	Psychiatric	102
	GRIA1	Alcohol dependence	Psychiatric	102
	GRIA1	Alcoholism (heaviness of drinking)	Psychiatric	102
	GRIA4	Alcohol dependence	Psychiatric	102
	GRM3	Alcohol and nictotine co-dependence	Psychiatric	102
	GRM3	Alcohol dependence	Psychiatric	102
	GRM7	Alcohol dependence	Psychiatric	102
	GRIP1	Substance dependence	Psychiatric	102
	GRIA4	Epilepsy	Neurological	102
	GRIK1	Epilepsy	Neurological	102
	GRIK1	Multiple sclerosis	Neurological	102
	GRM7	Migraine	Neurological	102
	GRM7	Brain size	Neurological	102
	GRM7	Multiple sclerosis	Neurological	102
	GRIA3	Parkinson`s disease	Neurological	102
	GRIA4	Parkinson`s disease	Neurological	102
	GRM7	Parkinson`s disease	Neurological	102

101	NSF	Parkinson`s disease	Neurological	102
	GRIA1	Cognitive impairment induced by topiramate	Neurological	102
	GRIA1	Cognitive decline	Neurological	102
	GRIA2	Cognitive impairment induced by topiramate	Neurological	102
	GRIA4	Cognitive impairment induced by topiramate	Neurological	102
	GRM7	Personality dimensions	Neurological	102
	GRM7	Cognitive impairment induced by topiramate	Neurological	102
	GRM7	Cognitive performance	Neurological	102
	SDC2	Cognitive performance	Neurological	102
	GRIP1	Cognitive impairment induced by topiramate	Neurological	102
	GRM3	Intelligence	Neurological	102
	GRIK1	Body mass index	Neurological	102
	GRM7	Body mass index	Neurological	102
	GRIK1	Mathematical ability	Neurological	102
	GRIK1	Schizophrenia (cytomegalovirus infection interaction)	Neurological	102
	GRM7	Schizophrenia	Neurological	102
	GRIA1	Alzheimer`s disease	Neurological	102
	GRIA4	Alzheimer`s disease (late onset)	Neurological	102
	GRM7	Alzheimer`s disease	Neurological	102
	GRIP1	Alzheimer`s disease	Neurological	102
	GRIA3	Amyotrophic Lateral Sclerosis	Neurological	102
	GRM3	Amyotrophic Lateral Sclerosis	Neurological	102
	GRM7	Amyotrophic Lateral Sclerosis	Neurological	102
	GRIP1	Amyotrophic Lateral Sclerosis	Neurological	102
	GRIK1	Autism spectrum disorder, attention deficit-hyperactivity disorder, bipolar disorder, major depressive disorder, and schizophrenia (combined)	Neurological	102
	GRIA4	Response to statin therapy	Neurological	102
	GRM3	Response to statin therapy	Neurological	102
	GRIA3	Mental retardation, X-linked 94, 300699	Neurological	102
102	GRIK1	Response to gemcitabine or arabinosylcytosin in blood cell lines	Cancer	102
	GRIA1	Asparaginase hypersensitivity in acute lymphoblastic leukemia	Cancer	102
	GRIK1	Breast cancer	Cancer	102
	GRIK1	Paclitaxel sensitivity in NCI60 cancer cell lines	Cancer	102
	GRM7	Prostate cancer	Cancer	102
	GRM7	Breast cancer	Cancer	102
	GRM7	Melanoma	Cancer	102
	GRM7	Cisplatin and carboplatin cytotoxicity, in blood cell lines	Cancer	102
	NSF	Prostate cancer	Cancer	102
	NSF	Ovarian cancer in BRCA1 mutation carriers	Cancer	102
	GRIK1	Leukocyte Counts	Cancer	102
	GRIA3	Response to taxane treatment (placlitaxel)	Cancer	102
	GRIA4	Response to taxane treatment (placlitaxel)	Cancer	102
	GRIK1	Nasopharyngeal carcinoma	Cancer	102
	GRM7	Response to taxane treatment (placlitaxel)	Cancer	102
	GRIK1	Hepatocellular carcinoma	Cancer	102

	GRIA4	Lymphocyte counts	Cancer	102
103	GRIA4	Matrix metalloproteinase levels	Cardiovascular	102
	GRM3	Response to statin treatment (atorvastatin), change in cholesterol levels	Cardiovascular	102
	GRM7	Aortic root size	Cardiovascular	102
	GRIP1	Birth weight	Cardiovascular	102
	GRIA1	Stroke	Cardiovascular	102
	GRIK1	Cardiovascular disease adverse events in renal patients treated with calcineurin inhibitors	Cardiovascular	102
	SDC2	Stroke	Cardiovascular	102
	SDC2	Lipoproteins	Cardiovascular	102
	GRIP1	Myocardial Infarction	Cardiovascular	102
	GRM7	Blood pressure, CVD RF and other traits	Cardiovascular	102
	SDC2	Blood pressure, CVD RF and other traits	Cardiovascular	102
	GRIP1	Blood pressure, CVD RF and other traits	Cardiovascular	102
	GRIK1	Leukocyte Counts	Cardiovascular	102
	GRIA4	Glycosylated haemoglobin levels	Cardiovascular	102
	GRIA4	Serum albumin level	Cardiovascular	102
	GRIA1	Sudden cardiac arrest	Cardiovascular	102
	GRIP1	Platelet counts	Cardiovascular	102
	GRIA1	Coronary Artery Disease	Cardiovascular	102
	GRM7	Coronary heart disease	Cardiovascular	102
	SDC2	Coronary heart disease	Cardiovascular	102
	GRIA4	Response to statin therapy	Cardiovascular	102
	GRM3	Response to statin therapy	Cardiovascular	102
	GRIA4	Lymphocyte counts	Cardiovascular	102
104	GNA12	Breast cancer and prostate cancer	Cancer	103
	SIM1	Pancreatic cancer	Cancer	103
	SIM1	Oral cancers (chewing tobacco related)	Cancer	103
	GNA12	Lymphocyte counts	Cancer	103
	ARNT	Leukemia, acute myeloblastic	Cancer	103
	AIP	Pituitary adenoma, ACTH-secreting, 219090	Cancer	103
	AIP	Pituitary adenoma, growth hormone-secreting, 102200	Cancer	103
	AIP	Pituitary adenoma, prolactin-secreting, 600634	Cancer	103
105	CDC37	Soluble ICAM-1	Psychiatric	103
	FKBP4	Suicide attempts in bipolar disorder	Psychiatric	103
	SIM2	Suicide attempts in bipolar disorder	Psychiatric	103
	GNA12	Alzheimer`s disease (late onset)	Psychiatric	103
	SIM2	Brain structure	Psychiatric	103
	GNA12	Parkinson`s disease	Psychiatric	103
	CDC37	Parkinson`s disease (familial)	Psychiatric	103
106	ANXA6	Systemic lupus erythematosus	Immunological	106
	RASA1	HIV-1 control	Immunological	106
	ANXA6	Taste perception	Immunological	106
	ANXA6	Lymphocyte counts	Immunological	106
	RGL1	Lymphocyte counts	Immunological	106

	RASK	Leukemia, acute myelogenous	Immunological	106
107	SHOC2	Urinary metabolites	Renal	106
	RGL1	Urinary metabolites	Renal	106
	RASK	Noonan syndrome 3, 609942	Renal	106
	RASH	Bladder cancer, somatic, 109800	Renal	106
	RASK	Bladder cancer, 109800	Renal	106
108	RASA1	Capillary malformation-arteriovenous malformation, 608354	Cardiovascular	106
	RASA1	Parkes Weber syndrome, 608355	Cardiovascular	106
	ANXA6	Lymphocyte counts	Cardiovascular	106
	RGL1	Lymphocyte counts	Cardiovascular	106
	RASK	Noonan syndrome 3, 609942	Cardiovascular	106
109	RASK	Noonan syndrome 3, 609942	Dermatological	106
	RASH	Costello syndrome, 218040	Dermatological	106
	RASK	Costello syndrome, 218040	Dermatological	106
	RASA1	Parkes Weber syndrome, 608355	Dermatological	106
	ANXA6	Systemic lupus erythematosus	Dermatological	106
110	EGFR	Bipolar disorder	Psychiatric	110
	JAK2	Depression (quantitative trait)	Psychiatric	110
	INSR	Schizophrenia	Psychiatric	110
	IRS1	Sudden cardiac arrest	Psychiatric	110
	SHC1	White blood cell count	Psychiatric	110
	EGFR	HDL cholesterol	Psychiatric	110
	PDE4A	Coronary heart disease	Psychiatric	110
	EGF	Alzheimer`s disease	Psychiatric	110
	EGFR	Alzheimer`s disease (late onset)	Psychiatric	110
	EGFR	Alzheimer`s disease	Psychiatric	110
	GHR	Alzheimer`s disease	Psychiatric	110
	JAK2	Alzheimer`s disease (late onset)	Psychiatric	110
	INSR	Amyotrophic Lateral Sclerosis	Psychiatric	110
	EGF	Menopause (age at onset)	Psychiatric	110
	EGFR	Alcoholism (heaviness of drinking)	Psychiatric	110
	INSR	Alcohol dependence	Psychiatric	110
	PDE4A	Soluble ICAM-1	Psychiatric	110
	GHR	Information processing speed	Psychiatric	110
	SH2B2	Information processing speed	Psychiatric	110
	SH2B1	Smoking behavior	Psychiatric	110
	PTN12	Colon cancer	Cancer	110
	STA5B	Leukemia, acute promyelocytic, STAT5B/RARA type	Cancer	110
	EGFR	Prostate cancer	Cancer	110
	EGFR	Cervical cancer	Cancer	110
	LYN	Prostate cancer	Cancer	110
	JAK2	Myeloproliferative neoplasms	Cancer	110
	EGFR	Chemotherapeutic response (cytarabine, 5`deoxyfluorouridine, carboplatin, cisplatin), in blood cell lines	Cancer	110
	LYN	Chemotherapeutic response (cytarabine, 5`deoxyfluorouridine, carboplatin,	Cancer	110

111		cisplatin), in blood cell lines		
	JAK2	Leukemia, acute myelogenous, 601626	Cancer	110
	JAK2	Polycythemia vera, 263300	Cancer	110
	JAK2	Thrombocythemia, essential, 187950	Cancer	110
	JAK2	Myelofibrosis, idiopathic, 254450	Cancer	110
	JAK2	Myeloproliferative disorder with erythrocytosis	Cancer	110
	EGFR	Adenocarcinoma of lung, response to tyrosine kinase inhibitor in, 211980	Cancer	110
	EGFR	Nonsmall cell lung cancer, response to tyrosine kinase inhibitor in, 211980	Cancer	110
	EGFR	Nonsmall cell lung cancer, susceptibility to, 211980	Cancer	110
	EGF	Menopause (age at onset)	Cancer	110
	IRS1	Lymphocyte counts	Cancer	110
112	IRS1	Coronary artery disease, susceptibility to	Cardiovascular	110
	EGFR	HDL particle features	Cardiovascular	110
	INSR	Aortic root size	Cardiovascular	110
	IRS2	Response to statin treatment (atorvastatin), change in cholesterol levels	Cardiovascular	110
	EGF	Cardiovascular disease	Cardiovascular	110
	EGF	Blood pressure, CVD RF and other traits	Cardiovascular	110
	GHR	Blood pressure, CVD RF and other traits	Cardiovascular	110
	IRS2	Body Fat Distribution	Cardiovascular	110
	EGFR	Chemotherapeutic response (cytarabine, 5`deoxyfluorouridine, carboplatin, cisplatin), in blood cell lines	Cardiovascular	110
	LYN	Chemotherapeutic response (cytarabine, 5`deoxyfluorouridine, carboplatin, cisplatin), in blood cell lines	Cardiovascular	110
	INSR	Thyroid hormone levels	Cardiovascular	110
	JAK2	Budd-Chiari syndrome, 600880	Cardiovascular	110
	IRS1	Sudden cardiac arrest	Cardiovascular	110
	SHC1	White blood cell count	Cardiovascular	110
	EGFR	HDL cholesterol	Cardiovascular	110
	PDE4A	Coronary heart disease	Cardiovascular	110
	INSR	Triglycerides	Cardiovascular	110
	IRS1	Lymphocyte counts	Cardiovascular	110
113	INSR	Diabetes mellitus, insulin-resistant, with acanthosis nigricans, 610549	Endocrine	110
	IRS1	Diabetes mellitus, noninsulin-dependent, 125853	Endocrine	110
	STA5B	Growth hormone insensitivity with immunodeficiency, 245590	Endocrine	110
	IRS2	Diabetes mellitus, noninsulin-dependent, 125853	Endocrine	110
	INSR	Polycystic ovary syndrome	Endocrine	110
	INSR	Type 2 diabetes	Endocrine	110
	PDE4A	Type 1 diabetes	Endocrine	110
	INSR	Hypothyroidism	Endocrine	110
	INSR	Thyroid hormone levels	Endocrine	110
	INSR	Leprechaunism, 246200	Endocrine	110
	INSR	Rabson-Mendenhall syndrome, 262190	Endocrine	110
	GHR	Increased responsiveness to growth hormone	Endocrine	110
	GHR	Laron dwarfism, 262500	Endocrine	110
	GHR	Short stature, autosomal dominant, with normal serum growth hormone binding protein	Endocrine	110

	GHR	Short stature, idiopathic	Endocrine	110
	EGFR	Diabetic retinopathy	Endocrine	110
	INSR	Diabetic retinopathy	Endocrine	110
	INSR	Hyperinsulinemic hypoglycemia, familial, 5, 609968	Endocrine	110
114	PTN1	Abdominal body fat distribution, modifier of, 609830	Metabolic	110
	PTN1	Insulin resistance, susceptibility to	Metabolic	110
	INSR	Hyperinsulinemic hypoglycemia, familial, 5, 609968	Metabolic	110
	GHR	Iron status biomarkers	Metabolic	110
	INSR	Obesity (extreme)	Metabolic	110
	LYN	Weight	Metabolic	110
	IRS2	Weight	Metabolic	110
	SH2B1	Obesity (extreme)	Metabolic	110
	SH2B1	Weight	Metabolic	110
	SH2B1	Obesity	Metabolic	110
	INSR	Triglycerides	Metabolic	110
115	LYN	Parkinson`s disease (familial)	Neurological	110
	GHR	Information processing speed	Neurological	110
	SH2B2	Information processing speed	Neurological	110
	EGFR	Glioma	Neurological	110
	EGF	Progressive supranuclear palsy	Neurological	110
	SH2B1	Body mass index	Neurological	110
	INSR	Schizophrenia	Neurological	110
	EGF	Alzheimer`s disease	Neurological	110
	EGFR	Alzheimer`s disease (late onset)	Neurological	110
	EGFR	Alzheimer`s disease	Neurological	110
	GHR	Alzheimer`s disease	Neurological	110
	JAK2	Alzheimer`s disease (late onset)	Neurological	110
	INSR	Amyotrophic Lateral Sclerosis	Neurological	110
	EGF	Menopause (age at onset)	Neurological	110
116	ACTN1	Suicide attempts in bipolar disorder	Psychiatric	111
	DACH1	White matter integrity	Psychiatric	111
	KLC2	Schizophrenia(treatment response to risperidone)	Psychiatric	111
	CBLC	Lipoprotein-associated phospholipase A2 activity and mass	Psychiatric	111
	ACTN1	Coronary heart disease	Psychiatric	111
	TOP1	Coronary heart disease	Psychiatric	111
	ACTN1	Brain structure	Psychiatric	111
	ATF3	Alzheimer`s disease (late onset)	Psychiatric	111
	CBLC	Alzheimer`s disease	Psychiatric	111
	ITCH	Alzheimer`s disease (late onset)	Psychiatric	111
	DAXX	Cholesterol	Psychiatric	111
	TOP1	Cholesterol, total	Psychiatric	111
	SAE1	Multiple sclerosis	Neurological	111
	TOP1	Taste perception	Neurological	111
	IKZF3	Myasthenia gravis	Neurological	111

117	PML	Progranulin levels	Neurological	111
	ITCH	Narcolepsy	Neurological	111
	DACH1	White matter integrity	Neurological	111
	KLC2	Schizophrenia(treatment response to risperidone)	Neurological	111
	ACTN1	Brain structure	Neurological	111
	ATF3	Alzheimer`s disease (late onset)	Neurological	111
	CBLC	Alzheimer`s disease	Neurological	111
	ITCH	Alzheimer`s disease (late onset)	Neurological	111
	MK10	Epileptic encephalopathy, Lennox-Gastaut type, 606369	Neurological	111
118	ATF3	Adverse response to chemotherapy (neutropenia/leucopenia) (cyclophosphamide)	Hematological	111
	ATF3	Adverse response to chemotherapy (neutropenia/leucopenia) (all anthracycline-based drugs)	Hematological	111
	DACH1	Adverse response to chemotherapy (neutropenia/leucopenia) (all anthracycline-based drugs)	Hematological	111
	ITCH	Protein C levels	Hematological	111
	IKZF3	Self-reported allergy	Hematological	111
	ACTN1	Blood pressure, CVD RF and other traits	Hematological	111
	DTX1	Blood pressure, CVD RF and other traits	Hematological	111
	PML	Blood Pressure	Hematological	111
	PML	Lymphocyte counts	Hematological	111
119	TOP1	DNA topoisomerase I, camptothecin-resistant	Metabolic	111
	TOP2A	DNA topoisomerase II, resistance to inhibition of, by amsacrine	Metabolic	111
	SUMO3	Waist Circumference	Metabolic	111
	IKZF3	Lipid traits	Metabolic	111
	DAXX	Cholesterol	Metabolic	111
	TOP1	Cholesterol, total	Metabolic	111
120	DACH1	Lung function	Respiratory	111
	PML	Pulmonary function	Respiratory	111
	IKZF3	Asthma (childhood onset)	Respiratory	111
	IKZF3	Asthma	Respiratory	111
	PML	Lymphocyte counts	Respiratory	111
	ACTN1	Smoking behavior	Respiratory	111
	IKZF3	Systemic lupus erythematosus and Systemic sclerosis	Respiratory	111
	DAXX	Rheumatoid arthritis	Respiratory	111
121	FEN1	Response to statin therapy	Neurological	112
	UBE2A	Mental retardation, X-linked syndromic	Neurological	112
	ATR	Seckel syndrome 1, 210600	Neurological	112
	MSH3	Chronic fatigue syndrome	Neurological	112
	PCNA	Personality dimensions	Neurological	112
	UBE2A	Brain connectivity	Neurological	112
	RAD18	Personality dimensions	Neurological	112
	ATR	Body mass index	Neurological	112
	MSH6	Mismatch repair cancer syndrome, 276300	Neurological	112
	MSH2	Mismatch repair cancer syndrome, 276300	Neurological	112
	MSH6	Endometrial cancer, familial, 608089	Cancer	112

122	MSH6	Ovarian cancer, endometrioid type, 604370	Cancer	112
	MSH3	Endometrial carcinoma	Cancer	112
	MSH3	Mammographic density	Cancer	112
	FEN1	Lung cancer	Cancer	112
	POLH	Xeroderma pigmentosum, variant type, 278750	Cancer	112
	MSH6	Colorectal cancer, hereditary nonpolyposis, type 5	Cancer	112
	MSH2	Colorectal cancer, hereditary nonpolyposis, type 1, 120435	Cancer	112
	MSH6	Mismatch repair cancer syndrome, 276300	Cancer	112
	MSH2	Mismatch repair cancer syndrome, 276300	Cancer	112
	MSH2	Muir-Torre syndrome, 158320	Cancer	112
123	NFKB1	Schizophrenia (treatment refractory)	Neurological	113
	BCL3	Alzheimer`s disease	Neurological	113
	BCL3	Alzheimer`s disease (late onset)	Neurological	113
	RELB	Alzheimer`s disease (late onset)	Neurological	113
	PIAS3	Contrast sensitivity	Neurological	113
	GFI1	Multiple sclerosis	Neurological	113
124	LIS1	Lissencephaly-1, 607432	Neurological	115
	LIS1	Subcortical laminar heterotopia	Neurological	115
	PGK1	Phosphoglycerate kinase 1 deficiency, 300653	Neurological	115
	UBA1	Spinal muscular atrophy, X-linked 2, infantile, 301830	Neurological	115
	DISC1	Neuranatomic and neurocognitive phenotypes	Neurological	115
	DISC1	Alzheimer`s disease	Neurological	115
	DISC1	Alzheimer`s disease (late onset)	Neurological	115
	DISC1	Schizophrenia	Neurological	115
	DISC1	Suicide, with and without major depression	Neurological	115
	DISC1	Smoking cessation	Neurological	115
125	PRKN2	Parkinson disease, juvenile, type 2, 600116	Neurological	116
	PRKN2	Leprosy, susceptibility to, 607572	Neurological	116
	RBP2	Schizophrenia(age at onset)	Neurological	116
	SNCAP	Parkinson disease, 168600	Neurological	116
126	ADRB3	Atrial fibrillation	Cardiovascular	120
	DCC	Aortic root size	Cardiovascular	120
	DCC	Myocardial Infarction	Cardiovascular	120
	DOCK1	Stroke	Cardiovascular	120
	DOCK1	Hypertension (essential hypertension)	Cardiovascular	120
	DOCK1	Blood pressure, CVD RF and other traits	Cardiovascular	120
	DCC	Bone mass and geometry	Cardiovascular	120
	NEO1	Body Fat Distribution	Cardiovascular	120
	VINC	Cardiomyopathy, dilated, 1W, 611407	Cardiovascular	120
	DCC	Platelet counts	Cardiovascular	120
	DCC	Coronary heart disease	Cardiovascular	120
	DOCK1	Coronary Artery Disease	Cardiovascular	120
	DOCK1	Coronary heart disease	Cardiovascular	120
	DOCK1	Response to statin therapy	Cardiovascular	120

	DCC	Behcet`s disease	Cardiovascular	120
	SRC	Lymphocyte counts	Cardiovascular	120
127	DOCK3	Bipolar disorder	Psychiatric	120
	DCC	Suicide attempts in bipolar disorder	Psychiatric	120
	DOCK1	Suicide attempts in bipolar disorder	Psychiatric	120
	DCC	Schizophrenia	Psychiatric	120
	DOCK1	White matter integrity	Psychiatric	120
	DOCK3	Schizophrenia	Psychiatric	120
	DCC	Platelet counts	Psychiatric	120
	DCC	Coronary heart disease	Psychiatric	120
	DOCK1	Coronary Artery Disease	Psychiatric	120
	DOCK1	Coronary heart disease	Psychiatric	120
	DCC	Alzheimer`s disease	Psychiatric	120
	DCC	Brain structure	Psychiatric	120
	DOCK1	Alzheimer`s disease	Psychiatric	120
	BCAR1	Alzheimer`s disease (late onset)	Psychiatric	120
	DCC	Amyotrophic Lateral Sclerosis	Psychiatric	120
	NEO1	Amyotrophic Lateral Sclerosis	Psychiatric	120
	DOCK1	Response to statin therapy	Psychiatric	120
	DCC	Personality dimensions	Psychiatric	120
	DCC	Cognitive impairment induced by topiramate	Psychiatric	120
	DOCK1	Cognitive test performance	Psychiatric	120
	NEO1	Cognitive impairment induced by topiramate	Psychiatric	120
	BCAR1	Cognitive performance	Psychiatric	120
	DCC	Alcoholism (heaviness of drinking)	Psychiatric	120
	DCC	Alcoholism (alcohol use disorder factor score)	Psychiatric	120
	DOCK1	Alcohol dependence	Psychiatric	120
	DOCK3	Alcohol dependence	Psychiatric	120
128	ADRB3	Obesity, susceptibility to, 601665	Metabolic	120
	DOCK1	Apolipoprotein Levels	Metabolic	120
	DCC	Obesity-related traits	Metabolic	120
	DOCK1	Iron levels	Metabolic	120
	DOCK1	Magnesium levels	Metabolic	120
	DOCK1	Weight	Metabolic	120
	DCC	Bone mass and geometry	Metabolic	120
	NEO1	Body Fat Distribution	Metabolic	120
129	DCC	Body mass index (interaction)	Neurological	120
	DOCK1	Body mass index	Neurological	120
	SRC	Colon cancer, advanced (3)	Neurological	120
	VINC	Cardiomyopathy, dilated, 1W, 611407	Neurological	120
	DCC	Schizophrenia	Neurological	120
	DOCK1	White matter integrity	Neurological	120
	DOCK3	Schizophrenia	Neurological	120
	DCC	Alzheimer`s disease	Neurological	120

	DCC	Brain structure	Neurological	120
	DOCK1	Alzheimer`s disease	Neurological	120
	BCAR1	Alzheimer`s disease (late onset)	Neurological	120
	DCC	Amyotrophic Lateral Sclerosis	Neurological	120
	NEO1	Amyotrophic Lateral Sclerosis	Neurological	120
	DOCK1	Response to statin therapy	Neurological	120
	NPHP1	Joubert syndrome 4, 609583	Neurological	120
	DCC	Personality dimensions	Neurological	120
	DCC	Cognitive impairment induced by topiramate	Neurological	120
	DOCK1	Cognitive test performance	Neurological	120
	NEO1	Cognitive impairment induced by topiramate	Neurological	120
	BCAR1	Cognitive performance	Neurological	120
	DCC	Response to mTOR inhibitor (everolimus)	Neurological	120
	DOCK1	Response to mTOR inhibitor (everolimus)	Neurological	120
	DCC	Behcet`s disease	Neurological	120
130	ATR	Red blood cell traits	Hematological	121
	BRCA2	Fanconi anemia, complementation group D1, 605724	Hematological	121
	BLM	Bloom syndrome, 210900	Hematological	121
	ATR	Seckel syndrome 1, 210600	Hematological	121
	RAD51	Lymphocyte counts	Hematological	121
131	PLD1	Response to gemcitabine or arabinosylcytosin in blood cell lines	Cancer	122
	NSF	Prostate cancer	Cancer	122
	NSF	Ovarian cancer in BRCA1 mutation carriers	Cancer	122
	EHD3	Testosterone levels	Cancer	122
	EHD3	Estradiol levels	Cancer	122
	SYT1	Response to taxane treatment (placlitaxel)	Cancer	122
	STX7	Lymphocyte counts	Cancer	122
132	NSF	Red blood cell traits	Hematological	122
	STX4	Acenocoumarol maintenance dosage	Hematological	122
	STX1B	Red blood cell traits	Hematological	122
	STX1B	Acenocoumarol maintenance dosage	Hematological	122
	TRIM9	Blood pressure, CVD RF and other traits	Hematological	122
	EHD3	Platelet counts	Hematological	122
	EHD3	Mean platelet volume	Hematological	122
	STX7	Lymphocyte counts	Hematological	122
133	PLD1	Response to statin treatment (atorvastatin), change in cholesterol levels	Cardiovascular	122
	SYT1	Aortic root size	Cardiovascular	122
	PLD1	Tunica Media	Cardiovascular	122
	VAMP3	Stroke	Cardiovascular	122
	TRIM9	Blood pressure, CVD RF and other traits	Cardiovascular	122
	STX4	Warfarin maintenance dose	Cardiovascular	122
	STX4	Warfarin responsiveness (phenprocoumon)	Cardiovascular	122
	STX1B	Warfarin responsiveness (phenprocoumon)	Cardiovascular	122
	EHD3	Platelet counts	Cardiovascular	122

	TRIM9	Coronary Artery Disease	Cardiovascular	122
	TRIM9	Coronary heart disease	Cardiovascular	122
	EHD3	Mean platelet volume	Cardiovascular	122
	STX7	Lymphocyte counts	Cardiovascular	122
134	PLD1	Metabolite levels	Endocrine	122
	PLD1	Type 2 diabetes	Endocrine	122
	SYT1	Insulin-related traits	Endocrine	122
	SYT1	Type 2 diabetes	Endocrine	122
	SYT1	Type 2 diabetes and 6 quantitative traits	Endocrine	122
	STX1B	Type 2 diabetes	Endocrine	122
	TRIM9	Type 2 diabetes	Endocrine	122
135	PLD1	Bipolar disorder and schizophrenia	Psychiatric	122
	PLD1	Bipolar disorder	Psychiatric	122
	TRIM9	Bipolar disorder	Psychiatric	122
	SYT1	Depression (quantitative trait)	Psychiatric	122
	CPLX1	Major depressive disorder	Psychiatric	122
	TRIM9	Nicotine smoking	Psychiatric	122
	STX4	Warfarin maintenance dose	Psychiatric	122
	STX4	Warfarin responsiveness (phenprocoumon)	Psychiatric	122
	STX1B	Warfarin responsiveness (phenprocoumon)	Psychiatric	122
	EHD3	Platelet counts	Psychiatric	122
	TRIM9	Coronary Artery Disease	Psychiatric	122
	TRIM9	Coronary heart disease	Psychiatric	122
	EHD3	Mean platelet volume	Psychiatric	122
	SYT1	Electroencephalogram traits, in brain	Psychiatric	122
	STX7	Alcohol dependence	Psychiatric	122
	SYT1	Creutzfeldt-Jakob disease (variant)	Psychiatric	122
	NSF	Parkinson`s disease	Psychiatric	122
	TRIM9	Psychosis (atypical)	Psychiatric	122
	TRIM9	Narcolepsy	Psychiatric	122
136	TBK1	Major depressive disorder (broad)	Psychiatric	123
	MAVS	Depression (quantitative trait)	Psychiatric	123
	TANK	Tourette syndrome	Psychiatric	123
	PELI2	Schizophrenia	Psychiatric	123
	IRAK1	Alzheimer`s disease	Psychiatric	123
	TRAF3	Alzheimer`s disease	Psychiatric	123
	TRAF1	Multiple sclerosis	Psychiatric	123
	TANK	Parkinson`s disease	Psychiatric	123
	TRAF3	Brain imaging in schizophrenia (interaction)	Psychiatric	123
	NCOR2	Suicide attempts in bipolar disorder	Psychiatric	127
	NCOR2	Cocaine dependence	Psychiatric	127
	HDAC5	Cocaine dependence	Psychiatric	127
	PPARG	Lipoprotein-associated phospholipase A2 activity change in response to statin therapy	Psychiatric	127
	PPARG	Plasminogen activator inhibitor type 1 levels (PAI-1)	Psychiatric	127

137	PPARG	Coronary heart disease	Psychiatric	127
	PPARG	Alzheimer`s disease	Psychiatric	127
	NCOR2	Alzheimer`s disease (late onset)	Psychiatric	127
	NCOR2	Cognitive performance	Psychiatric	127
	PPARG	Memory performance	Psychiatric	127
	NCOR2	Progranulin levels	Psychiatric	127
	PPARG	Alcohol dependence	Psychiatric	127
138	PRIO	Gerstmann-Straussler disease, 137440	Neurological	131
	PRIO	Huntington disease-like 1, 603218	Neurological	131
	PRIO	Prion disease with protracted course, 606688	Neurological	131
	PRIO	Creutzfeldt-Jakob disease, 123400	Neurological	131
	GNA12	Parkinson`s disease	Neurological	131
	CDC37	Parkinson`s disease (familial)	Neurological	131
	SIM1	Intracranial aneurysm	Neurological	131
	PRIO	Insomnia, fatal familial, 600072	Neurological	131
139	SIM1	Pancreatic cancer	Endocrine	131
	AIP	Pituitary adenoma, ACTH-secreting, 219090	Endocrine	131
	AIP	Pituitary adenoma, growth hormone-secreting, 102200	Endocrine	131
	AIP	Pituitary adenoma, prolactin-secreting, 600634	Endocrine	131
	SIM1	Obesity, severe, 601665	Endocrine	131
	PPID	Metabolite levels	Endocrine	131
	SIM1	Metabolite levels	Endocrine	131

D_M denotes index of disease modules, and T_M denotes topological modules.

Supplementary table 4: Summary of Pathway enrichment analysis of topological modules.

T_Modules	Gene counts	Hit counts	%	Top 1 term	Benjamini
Module 3	8	6	75	Membrane Trafficking	1.99E-10
Module 4	6	3	50	Angiogenesis	0.05
Module 6	6	4	66.7	ALK in cardiac myocytes	0.001993089
Module 7	4	4	100	Nucleotide excision repair	1.21E-06
Module 8	5	4	80	3' -UTR-mediated translational regulation	1.25E-04
Module 12	6	3	50	Hedgehog signaling pathway	3.57E-04
Module 14	4	3	75	Base excision repair	2.76E-04
Module 15	3	3	100	Basal transcription factors	9.21E-05
Module 16	24	18	75	Gene Expression	1.16E-17
Module 17	6	6	100	Adipocytokine signaling pathway	1.71E-09
Module 18	7	3	42.9	Map Kinase Inactivation of SMRT Corepressor	0.002227212
Module 19	9	7	77.8	ECM-receptor interaction	6.94E-09
Module 20	6	3	50	ECM-receptor interaction	0.001347711
Module 22	12	10	90.9	Metabolism of non-coding RNA	9.06E-21
Module 23	17	17	100	DNA Replication	1.09E-25
Module 24	4	3	75	Synaptic Transmission	5.61E-04
Module 26	5	5	100	Toll-like receptor signaling pathway	7.34E-07
Module 27	45	14	31.1	Cell cycle	1.23E-13

Module 29	5	5	100	Pathogenic Escherichia coli infection	4.26E-08
Module 30	4	4	100	Spliceosome	2.97E-05
Module 31	25	16	64	Transcription	4.71E-20
Module 34	18	17	94.4	MAPK signaling pathway	1.04E-19
Module 35	18	17	94.4	Proteasome	8.17E-33
Module 36	25	16	64	Proteasome	6.13E-29
Module 39	27	7	25.9	Mechanism of Protein Import into the Nucleus	1.42235E-10
Module 40	27	4	14.8	Regulation of eIF2	0.000463134
Module 41	5	4	80	Ribosome	9.68E-06
Module 42	22	11	50	Ubiquitin mediated proteolysis	4.26E-13
Module 45	5	5	100	MAPK signaling pathway	7.44E-05
Module 46	5	4	80	Ubiquitin mediated proteolysis	1.15E-04
Module 47	12	11	91.7	Tight junction	1.10E-14
Module 50	6	4	66.7	Synaptic_vesicle_trafficking	7.02333E-05
Module 51	11	10	90.9	3' -UTR-mediated translational regulation	4.75E-14
Module 52	5	5	100	Apoptosis	1.36E-06
Module 54	7	5	71.4	Metabolism of proteins	6.58E-04
Module 55	5	4	80	Jak-STAT signaling pathway	1.67E-04
Module 57	4	4	100	Cell adhesion molecules (CAMs)	0
Module 58	5	4	80	Axon guidance	1.88E-04
Module 60	8	7	87.5	Regulation of autophagy	6.78E-14
Module 62	5	5	100	Spliceosome	3.60E-07
Module 63	3	3	100	Adherens junction	4.53E-04
Module 64	6	4	66.7	Gene Expression	0.004330516
Module 65	3	3	100	Signaling by EGFR	5.62E-04
Module 66	5	3	60	DNA Repair	0
Module 67	7	7	100	RNA degradation	4.55E-12
Module 68	8	5	62.5	Mismatch repair	9.55E-10
Module 69	5	4	80	RNA polymerase	5.98E-07
Module 71	8	3	37.5	Integrin signalling pathway	0.01745058
Module 72	10	6	60	Membrane Trafficking	1.64E-08
Module 74	30	9	30	MAPK signaling pathway	7.91E-05
Module 75	19	8	42.1	Endocytosis	7.25E-07
Module 76	23	9	39.1	Alzheimer disease-presenilin pathway	1.00E-07
Module 79	13	9	69.2	Cyclins and Cell Cycle Regulation	7.18E-13
Module 80	23	10	43.5	Apoptosis	1.99E-10
Module 82	13	3	23.1	TGF-beta signaling pathway	0.000289415
Module 83	88	18	20.5	T cell receptor signaling pathway	9.24E-13
Module 85	23	9	39.1	Regulation of actin cytoskeleton	2.85E-06
Module 86	4	3	75	Cytosolic DNA-sensing pathway	0.007502646
Module 87	9	8	88.9	MAPK signaling pathway	1.63E-08
Module 88	19	4	21.1	MAPK signaling pathway	0.017005785
Module 90	34	7	20.6	Ionotropic glutamate receptor pathway	3.586E-6
Module 91	10	6	60	TGF-beta signaling pathway	9.84E-07

Module 92	11	4	36.4	Overview of telomerase RNA component gene hTerc Transcriptional Regulation	3.10079E-05
Module 94	11	4	36.4	Renal cell carcinoma	0.00015837
Module 97	7	5	71.4	The PRC2 Complex Sets Long-term Gene Silencing Through Modification of Histone Tails	4.04115E-09
Module 98	6	6	100	Angiogenesis	5.43E-06
Module 100	13	12	92.3	Chemokine signaling pathway	9.99E-16
Module 102	15	8	53.3	Synaptic Transmission	1.17E-10
Module 103	12	4	33.3	Ahr Signal Transduction Pathway	1.21446E-06
Module 105	4	3	75	Huntington's disease	0.007289998
Module 106	12	7	58.3	Pathways in cancer	7.00E-06
Module 110	21	7	33.3	Growth Hormone Signaling Pathway	7.59898E-08
Module 111	40	9	23.1	MAPK signaling pathway	0.000323456
Module 112	18	8	44.4	Mismatch repair	1.33393E-12
Module 113	11	5	45.5	Adipocytokine signaling pathway	1.87E-05
Module 114	6	5	83.3	Notch signaling pathway	1.27E-07
Module 115	10	3	30	Ether lipid metabolism	0.00541589
Module 116	6	4	66.7	Parkinson's disease	3.12E-05
Module 118	7	5	71.4	Oocyte meiosis	5.10E-06
Module 119	5	3	60	MAPK signaling pathway	0.039148472
Module 120	18	10	55.6	Integrin signalling pathway	5.53E-07
Module 121	8	6	75	Homologous recombination	1.66E-10
Module 122	26	14	53.9	SNARE interactions in vesicular transport	9.94E-26
Module 124	11	6	54.5	Ubiquitin mediated proteolysis	3.98E-06
Module 123	21	7	33.3	Small cell lung cancer	2.96E-06
Module 129	7	7	100	Endocytosis	4.15E-09
Module 131	16	4	25	Antigen processing and presentation	0.003315967
Module 135	5	5	100	Endocytosis	1.66E-06

T_Modules denotes index of Topological modules.
